# Long term impact of prophylactic antibiotic use before incision versus after cord clamping on children born by caesarean section: longitudinal study of UK electronic health records

**DOI:** 10.1136/bmj-2021-069704

**Published:** 2022-05-18

**Authors:** Dana Šumilo, Krishnarajah Nirantharakumar, Brian H Willis, Gavin M Rudge, James Martin, Krishna Gokhale, Rasiah Thayakaran, Nicola J Adderley, Joht Singh Chandan, Kelvin Okoth, Isobel M Harris, Ruth Hewston, Magdalena Skrybant, Jonathan J Deeks, Peter Brocklehurst

**Affiliations:** 1 Institute of Applied Health Research, University of Birmingham, Birmingham, UK; 2 Populations, Evidence and Technologies, Division of Health Sciences, Warwick Medical School, University of Warwick, Coventry, UK; 3 Midlands Health Data Research UK, University of Birmingham, Birmingham, UK; 4 West Midlands, UK; 5 NIHR Birmingham Biomedical Research Centre, University Hospitals Birmingham NHS Foundation Trust and University of Birmingham, Birmingham, UK

## Abstract

**Objective:**

To investigate the impact on child health up to age 5 years of a policy to use antibiotic prophylaxis for caesarean section before incision compared with after cord clamping.

**Design:**

Observational controlled interrupted time series study.

**Setting:**

UK primary and secondary care.

**Participants:**

515 945 children born in 2006-18 with linked maternal records and registered with general practices contributing to two UK primary care databases (The Health Improvement Network and Clinical Practice Research Datalink), and 7 147 884 children with linked maternal records in the Hospital Episode Statistics database covering England, of which 3 945 351 were linked to hospitals that reported the year of policy change to administer prophylactic antibiotics for caesarean section before incision rather than after cord clamping.

**Intervention:**

Fetal exposure to antibiotics shortly before birth (using pre-incision antibiotic policy as proxy) compared with no exposure.

**Main outcome measures:**

The primary outcomes were incidence rate ratios of asthma and eczema in children born by caesarean section when pre-incision prophylactic antibiotics were recommended compared with those born when antibiotics were administered post-cord clamping, adjusted for temporal changes in the incidence rates in children born vaginally.

**Results:**

Prophylactic antibiotics administered before incision for caesarean section compared with after cord clamping were not associated with a significantly higher risk of asthma (incidence rate ratio 0.91, 95% confidence interval 0.78 to 1.05) or eczema (0.98, 0.94 to 1.03), including asthma and eczema resulting in hospital admission (1.05, 0.99 to 1.11 and 0.96, 0.71 to 1.29, respectively), up to age 5 years.

**Conclusions:**

This study found no evidence of an association between pre-incision prophylactic antibiotic use and risk of asthma and eczema in early childhood in children born by caesarean section.

## Introduction

More than a quarter of deliveries in Europe and even higher proportions in North and Latin America are by caesarean section.^1^ Compared with vaginal delivery, caesarean section carries a substantially increased risk of maternal postpartum infections.^2^ This risk can be reduced by routine provision of prophylactic antibiotics.^3^ Evidence from randomised controlled trials shows that antibiotics are more effective at reducing the risk of maternal infectious morbidity after delivery if given before incision rather than after cord clamping.^4^ Since 2011, the National Institute for Health and Care Excellence has recommended prophylactic antibiotics before incision in mothers undergoing caesarean section.^5^ ^6^

Antibiotics given pre-incision cross the placenta and therefore babies are exposed to them around the time of birth^7^; and at a time when the human gut becomes colonised by microbes.^8^ Type of delivery is known to affect the composition of the gut microbiota in the neonatal period and during infancy.^9^ A growing body of evidence suggests that microbiota of the infant gut play a key role in the development of the immune system, which includes regulation of response to different antigens and inflammation.^10^ ^11^ ^12^ ^13^ Intrapartum antibiotics alter the intestinal microbiota of infants,^14^ although the effect on gut microbiota is less evident in children born by caesarean section.^15^ ^16^ Disruptions to the gut microbiota are associated with susceptibility to asthma, eczema, allergies, and other immune related diseases in childhood.^13^ ^17^ ^18^

We investigated the effect of a change in prophylactic antibiotic policy from after cord clamping to before incision on the incidence of allergic and other related health conditions in children born by caesarean section. To validate the methods used we also explored if the effects of reduced maternal infections during the postpartum period observed in randomised controlled trials could be replicated using routine healthcare data from the United Kingdom.

## Methods

### Study design

We performed a controlled interrupted time series study using anonymised linked healthcare records of mothers and their babies born between 2006 and 2018 in the UK covering a period before and after the 2011 national policy change on timing of antibiotic prophylaxis for caesarean section. The study protocol is published elsewhere.^19^

We compared the incidence rates of disease and other outcomes of interest in children born by caesarean section when pre-incision prophylactic antibiotics were recommended compared with those born when antibiotics were administered post-cord clamping. To account for temporal changes in the incidence rates from other factors, including the recording and diagnosis of outcomes of interest, we chose a control group of children born vaginally. These children would not have been routinely exposed to prophylactic antibiotics at delivery, but they would have been subject to the same temporal changes in clinical care and diagnostic thresholds as children born by caesarean section.

These changes over time could not be accounted for by a simple adjustment of confounders. This is because of uncertainty about all the drivers of all these changes, whether covariates exist without substantial missing data that could accurately describe these changes, and the functional form of the relationships between these covariates and outcomes of interest.

### Participants

We included the healthcare records of mother-baby pairs in the study if the child’s birth year was between 2006 and 2018, the child’s healthcare record could be linked to that of the mother, and delivery type (caesarean section or vaginal delivery) could be identified from the records. To ensure independence of observations, we randomly chose one of the children from twin and multiple births for inclusion. Children who were born between 2006 and 2014 were followed-up until 5 years of age, whereas children born between 2015 and 2018 were followed-up to a maximum of four years owing to data availability.

### Primary and secondary healthcare data sources and linkage

To investigate health outcomes recorded in primary care, we used two primary care databases, The Health Improvement Network (THIN) and the Clinical Practice Research Datalink (CPRD GOLD), which contain anonymised patient healthcare records from general practices and together cover more than 10% of the UK population.^20^ The databases include information on patient characteristics, consultations, diagnoses, and drug prescriptions.^21^ ^22^ Patients’ demographic characteristics and disease prevalence in these databases are broadly representative of the UK population.^23^ ^24^ We adapted previously published algorithms for linking mother and baby data^25^ ^26^ and for identification and exclusion of duplicate patient records from general practices contributing to both THIN and CPRD datasets.^20^ ^27^

To investigate more severe outcomes of interest, we used a separate anonymised Hospital Episode Statistics (HES) dataset, which captures information on hospital admissions in all National Health Service hospitals in England.^28^ For the purposes of this study, we used data for births for financial years 2005/06 to 2016/17 (from 1 April to 31 March each year), with the follow-up data for subsequent hospital admissions including data up to 2018/19. In addition to clinical diagnoses, procedures, and demographic and geographical information in HES, delivery records for mothers and birth records for babies contain additional information related to delivery, including delivery type, gestational age, and birth weight. We adapted a previously validated algorithm to link mother and baby data in HES.^29^ The supplementary appendix provides further details on primary and secondary healthcare data sources and data linkage.

### Exposure

Routine healthcare records do not contain information on the use of prophylactic antibiotics for caesarean section, including the timing of their use. More than 98% of women in the UK are given antibiotic prophylaxis for caesarean section.^30^ ^31^ We used the year of delivery to estimate the probability of babies being exposed to pre-incision antibiotics.

The type of delivery is well recorded in HES maternal records.^32^ Codes for delivery type in the Office of Population Censuses and Surveys Classification of Surgical Operations and Procedures fourth revision (OPCS-4) are missing for only 2% of births in HES data.^33^ When HES data were available we therefore used OPCS-4 codes to determine the type of delivery, and ICD-10 (international classification of diseases and related health problems, 10th revision) codes if OPCS-4 codes for delivery type were missing.

During the study period, 20.3% of general practices (162/797) in THIN and 55.3% of general practices (410/741) in CPRD contained linked anonymised data recorded in secondary care. For mother-baby pairs in both datasets with no secondary care data linkage we used Read codes (clinical codes used for recording information in primary care datasets) to determine the type of delivery. Although recording of delivery type in primary care records is not complete, it was accurate (when recorded, for 98% of births it matched the delivery type recorded in HES linked data).

For outcomes recorded in primary care, for each mother giving birth by caesarean section in the primary analysis we used the national uptake rates of pre-incision antibiotics policy in the year of birth to obtain a probability of receiving antibiotics pre-incision (see supplementary fig S1). We estimated the uptake of pre-incision antibiotics policy over the study period from a survey of all hospitals undertaking caesarean section in the UK. The survey was developed using a secure, web-based software platform REDCap,^34^ ^35^ and sent to clinical directors for maternity care asking whether their hospital’s policy stated that prophylactic antibiotics should be given before incision or after cord clamping, and, if antibiotics were given before incision, in which year the pre-incision antibiotics policy was implemented by the hospital. The clinical directors were also asked which antibiotics were currently used for prophylaxis at the time of caesarean section and if the same antibiotics were used before the policy change. The supplementary appendix provides further information about the survey.

For outcomes recorded in secondary care, we assumed that all mothers whose babies were born after the year when the local policy in each hospital was implemented were exposed to pre-incision antibiotics. We matched the national survey data to births in the HES dataset using the NHS organisation code for each hospital.

### Outcomes

We defined the outcomes for this study based on previous literature, through public and patient involvement via discussion groups and wider engagement with the public and with clinicians from an online survey.

The primary outcomes in this study were incidence of asthma and eczema. Both conditions have been frequently associated with antibiotic exposure and gut microbiota in infants.^13^ ^17^ ^18^ ^36^ ^37^ Asthma and eczema are commonly diagnosed in early childhood,^38^ ^39^ and recording of these conditions has been validated in electronic health records.^40^ ^41^ ^42^ Existing code lists were therefore used as a basis for defining asthma and eczema in this study. In line with the validation paper for eczema diagnosis in primary care reflecting the chronicity of this health condition, at least two prescriptions of eczema related treatment on separate dates were also required.^40^ In the HES dataset, asthma and eczema were defined if they were coded as the primary diagnoses for hospital admission.

We used the following process to create the overall code list for this study. For primary care data, we used Read clinical codes to define the outcomes, employing a robust code selection process consisting of compiling keywords and synonyms for each variable, using these to identify the main chapter and stem codes in the Read code dictionary, searching published articles and online Read code repositories, and refining the lists through consultation with the study team, including general practice and specialists in the discipline.

For data recorded in HES, we used ICD-10 and OPCS-4 codes to define the variables, with the code selection process consisting of screening published studies, checking the resources for clinical coders and technical documents for coding definitions used by the Office for National Statistics and the NHS,^43^ examining the use of codes in English admitted patient care data, and seeking consensus from the study team on the final list of codes to be used. The code lists are available at https://github.com/NIHR-Pre-incision-Antibiotics/ClinicalCodes.

Secondary outcomes included other allergic and allergy related health conditions, autoimmune health conditions, infections, other immune related diseases, neurodevelopmental conditions, less specific child health measures, and healthcare utilisation. Many of these outcomes in children were classed as exploratory based on weak evidence—for example, owing to a lack of longitudinal studies between intestinal microbiota and the outcome of interest. Secondary outcomes also included maternal infections in the postpartum period within six weeks of delivery.

Some of the outcomes are better captured or are only recorded in primary care or hospital data, therefore for these outcomes we restricted our analysis to these datasets. Supplementary table S1 shows the full list of outcomes that were analysed in each dataset. In HES analyses, outcomes were defined based on the main primary diagnosis for hospital admission, except for maternal outcomes, neonatal sepsis, and necrotising enterocolitis for which we included any mention of diagnosis in hospital admission episodes, as these conditions related to delivery often develop during the hospital stay.

### Covariates

To assess the validity of our model using children born vaginally as a control group, we explored if differential changes occurred in different maternal and child characteristics in relation to delivery type (caesarean section and vaginal delivery) over time. From the literature we identified the main covariates associated with the primary outcomes in children and maternal infections in the postnatal period. Maternal characteristics included age at delivery, ethnicity, parity, body mass index (BMI) before pregnancy, smoking status, area-based deprivation, allergy related conditions, and pregnancy and labour complications (antibiotic prescribing during pregnancy, premature rupture of membranes, manual placental removal or retained products of conception, and postpartum haemorrhage). Child characteristics included sex, gestational age, ethnicity, birth weight, breastfeeding status, other allergy related conditions and respiratory tract infections, and antibiotic prescribing in early childhood. Information on covariates, when available, was examined in both primary care (THIN and CPRD) and HES datasets. Recording of breastfeeding in routine healthcare data was incomplete; we therefore investigated trends in breastfeeding using data from the Care Quality Commission’s maternity surveys available from the UK Data Service and the National Perinatal Epidemiology Unit’s maternity surveys.^44^ ^45^ ^46^ No noticeable changes by delivery type over time were identified (see supplementary figs S4-24).

### Power estimates

We estimated that we would have more than 90% power to detect incidence rate ratios of 1.18 and 1.12 for asthma outcomes recorded in primary care (THIN and CPRD) and HES datasets, respectively, with a maximum of 15% underestimation from misclassification. The study protocol published elsewhere provides further details of the power calculation.^19^

### Statistical analyses

Primary and secondary outcomes are expressed as number of events per 1000 person years (calculated using number of children with the outcome of interest and length of time at risk). For each outcome, we calculated the length of time at risk as the time from birth to the earliest of the child’s fifth birthday, the date the child was lost to follow-up, the study end date (31 December 2018), or the date the outcome was diagnosed in the child. For maternal outcomes, we summarise the number and percentage of women with each outcome, by delivery type.

#### Analyses for outcomes recorded in primary care

We fitted a Poisson regression model to THIN and CPRD data, with an offset for exposure time, and included covariates for year of birth, age (year of life), interaction between year of birth and age, delivery type (caesarean section or vaginal delivery), and time antibiotic was administered (pre-incision or post-cord clamping). To allow for differential rates of diagnosis over time and age, we included covariates for year of birth and age, and their interaction. The children each had one observation for each year of life in which they were included in the study. For all caesarean section births, since the exact timing of antibiotics was not known, we used national survey data to estimate the probability of each child being exposed to pre-incision antibiotics. As the policy change was only applicable for births by caesarean section, we classified all births by vaginal delivery as being before the policy change (ie, unexposed to pre-incision antibiotics). Models provide estimates of incident rate ratios and corresponding 95% confidence intervals between the rate of outcomes in babies exposed to pre-incision antibiotics and the rate of outcomes in babies in the unexposed group (antibiotics administered post-cord clamping). For rare outcomes in our study population (fewer than 200 participants with a diagnosis over the study period), we present summary statistics only.

#### Analyses for outcomes recorded in HES

We fitted a Poisson regression model to HES data, with an offset for exposure time, and included covariates for year of birth, delivery type (caesarean section or vaginal delivery), and time antibiotic was administered (pre-incision or post-cord clamping). We classified the timing of antibiotics in relation to the policy change (before or after)—with the year of change identified from the national survey—which allowed the year of policy change to be different for each hospital. Only hospitals with a known year of change were included in the main analysis (about 55% of births). During the year of policy change, we excluded births from the analysis for each hospital. As the policy change was only applicable for births by caesarean section, we classified all births by vaginal delivery as occurring before the policy change (ie, unexposed to pre-incision antibiotics). We present incidence rate ratios with corresponding 95% confidence intervals comparing the rate of outcomes in births after the year of policy change (assumed to be exposed to pre-incision antibiotics) with the rate before the year of policy change (assumed to be unexposed to pre-incision antibiotics). For rare outcomes in our study population (fewer than 200 participants with a diagnosis over the study period), we present summary statistics only. For continuous outcomes, we fitted a linear regression model, estimating the difference in means between babies exposed to pre-incision antibiotics and those unexposed to pre-incision antibiotics.

#### Sensitivity and exploratory subgroup analyses

We conducted a series of different sensitivity analyses to assess the robustness of the results. In THIN and CPRD dataset, this included alternative definitions of the outcomes, alternative definition of antibiotic timing, data recording quality, and random effect for general practice. In HES, sensitivity analyses included an alternative definition of antibiotic timing, random effect for hospital, and exploratory discordant sibling analysis. For the primary outcomes, we additionally undertook prespecified exploratory subgroup analysis by type of caesarean section (elective or emergency) using an interaction term between antibiotic timing and caesarean section type. A prespecified exploratory subgroup analysis of the primary outcome by antibiotic type (co-amoxiclav alone, cefuroxime alone, and cefuroxime combined with metronidazole) was performed in HES data only. The supplementary appendix provides further details on the sensitivity analyses and subgroup analyses.

All statistical analysis was completed using Stata MP version 16.^47^

### Patient and public involvement

Patients and members of the public were involved from inception of this research. Two lay parent contributors were full members of the study team, and an independent patient and public involvement (PPI) contributor was a member of the project’s steering group. In addition, we held two wider PPI sessions with both mothers and mothers to be from a variety of backgrounds, and a consultation with members of the Royal College of Obstetricians and Gynaecologists Women’s Voices Involvement Panel and the British Intrapartum Care Society that included lay members through an online survey to refine the outcome list for this study. PPI helped us to confirm the importance of the research question and baby’s health as a main priority when deciding on birth options, and the importance of considering a wide range of health outcomes and their severity. Our PPI members are involved in the co-production of the lay summary and the dissemination plans for this study.

## Results

In total, 307 741 and 409 907 mother-baby pairs met the study inclusion criteria in THIN and CPRD GOLD databases, respectively, with type of delivery (caesarean section or vaginal delivery) recorded in either linked secondary care records or in primary care (see supplementary fig S2). After removal of duplicates, the final THIN and CPRD dataset included 515 945 mother-baby pairs, of which 57.2% (295 079/515 945) were from primary care datasets with linked secondary care data. More than a quarter (28.1%, 144 861/515 945 and 26.0%, 76 819/295 079) of all births were by caesarean section in the full THIN and CPRD dataset and the THIN and CPRD dataset with linked secondary care data used in sensitivity analysis, respectively.

In the HES dataset derived from hospital admissions for England, 7 222 967 mother-baby pairs met the study inclusion criteria and were successfully matched (see supplementary fig S3). After random inclusion of one baby from each multiple birth, the full dataset included 7 147 884 mother-baby pairs, of which 25.4% (1 813 799/7 147 884) involved caesarean section. The dataset used in the main analysis with linked information on hospital level prophylactic antibiotic policies included 3 945 351 mother-baby pairs, of which 25.4% (1 001 598/3 945 351) also involved caesarean section.

Table 1 summarises the numbers of births by delivery mode over the study period in each of the datasets.

**Table 1:** Numbers of births with known delivery type by year in THIN and CPRD dataset and HES dataset

Year	THIN-CPRD dataset*					HES dataset for England†			
	Full THIN-CPRD dataset		THIN-CPRD with secondary care linked data‡			Full HES dataset§		HES dataset with linked information on hospital prophylactic antibiotic policies	
	Caesarean section	Vaginal delivery	Caesarean section	Vaginal delivery		Caesarean section	Vaginal delivery	Caesarean section	Vaginal delivery
2005	-	-	-	-		98 209	313 235	47 996	152 839
2006	12 326	34 115	7352	21 721		135 839	422 260	67 733	210 919
2007	12 868	35 094	7815	22 637		138 606	421 346	69 263	214 954
2008	13 232	36 365	7950	23 089		146 365	447 045	71 086	220 393
2009	13 229	36 264	7733	22 933		150 427	455 737	75 608	232 258
2010	13 504	36 932	8081	23 627		157 367	474 019	84 371	255 288
2011	13 538	36 389	7877	23 065		157 196	475 408	89 557	270 032
2012	13 772	35 785	7739	21 961		159 710	471 507	94 338	279 465
2013	12 823	32 089	6808	19 005		154 794	449 047	91 822	265 662
2014	11 783	28 360	5876	15 552		156 090	439 405	93 579	263 055
2015	9946	22 945	4362	11 256		159 297	435 153	95 433	260 729
2016	8407	18 272	2973	7753		161 659	429 282	97 564	257 789
2017	7184	14 798	2223	5580		38 240	100 641	23 248	60 370
2018	2249	3676	30	81		-	-	-	-
Total	144 861	371 084	76 819	218 260		1 813 799	5 334 085	1 001 598	2 943 753

THIN=The Health Improvement Network; CPRD=Clinical Practice Research Datalink; HES=Hospital Episode Statistics.

* Overall number of general practices contributing to THIN and CPRD GOLD, and number of practices with secondary care linked data decreased over the study period, resulting in decreasing numbers of births in the THIN and CPRD dataset in later years. The low numbers in 2018 are due to only THIN primary care dataset contributing births until the end of the year, with secondary care linked data including births until March and CPRD data including births in January only.

† HES data were provided by NHS Digital for financial years 2005/06 to 2016/17 (from 1 April to 31 March for each year). Births for 2005 therefore include those from April to December, and births for 2017 include those from January to March during those years.

‡ This dataset was used in sensitivity analysis to evaluate the impact of data recording quality, restricting the dataset to mother-baby pairs with linked secondary care data as the most accurate source for delivery mode.

§ This dataset was used in sensitivity analysis of the timing of policy change for prophylactic antibiotics, not limiting it to the hospitals that had reported the year when the pre-incision antibiotic policy was implemented and included the overall probability of pre-incision antibiotic use in each year estimated from the national survey on the timing of antibiotic prophylaxis using responses from hospitals in England.

### Outcomes recorded in primary care

Table 2 summarises the numbers of children with a first diagnosis of the primary and secondary outcomes recorded in the primary care THIN and CPRD dataset, the follow-up time in person years, and the overall rate per 1000 person years. We observed a 9% decrease in asthma diagnosis (incidence rate ratio 0.91, 95% confidence interval 0.78 to 1.05) and a 2% decrease in eczema diagnosis (0.98, 0.94 to 1.03) in children exposed to pre-incision prophylactic antibiotics compared with children not exposed. These differences were not statistically significant.

**Table 2:** Number of children with different health outcomes of interest recorded in primary care (THIN and CPRD) dataset, overall rate per 1000 person years, and relative risk associated with pre-incision versus post-cord clamping prophylactic antibiotics

Outcomes	Total No (follow-up time in person years)	Overall rate per 1000 person years (95% CI)*	Pre-incision *v* post-cord clamping antibiotics† (based on national policy uptake %)	
			Incidence rate ratio (95% CI)	P value
**Primary outcomes**				
Asthma	16 540 (1 670 173)	9.90 (9.75 to 10.06)	0.91 (0.78 to 1.05)	0.18
Eczema	102 888 (1 430 708)	71.91 (71.48 to 72.35)	0.98 (0.94 to 1.03)	0.46
**Other allergic and allergy related conditions**				
Food allergy or intolerance	8609 (1 680 729)	5.12 (5.01 to 5.23)	1.02 (0.89 to 1.17)	0.76
Allergic rhinitis and conjunctivitis	10 511 (1 680 973)	6.25 (6.13 to 6.37)	0.98 (0.83 to 1.16)	0.81
>1 allergy related disease	20 146 (1 634 317)	12.33 (12.16 to 12.50)	0.99 (0.88 to 1.12)	0.85
Penicillin allergy‡	1020 (1 698 282)	0.60 (0.56 to 0.64)	0.68 (0.42 to 1.08)	0.10
Anaphylaxis‡	253 (1 700 221)	0.15 (0.13 to 0.17)	1.07 (0.43 to 2.63)	0.89
High risk of anaphylactic reaction‡	2365 (1 696 513)	1.39 (1.34 to 1.45)	0.81 (0.60 to 1.09)	0.17
**Autoimmune diseases**				
Type 1 diabetes‡	251 (1 700 377)	0.15 (0.13 to 0.17)	0.57 (0.20 to 1.67)	0.30
Coeliac disease‡	285 (1 700 259)	0.17 (0.15 to 0.19)	0.54 (0.21 to 1.39)	0.20
Juvenile idiopathic arthritis‡§	115 (1 700 555)	0.07 (0.06 to 0.08)	-	-
Scleroderma or systemic sclerosis‡§	≤5 (1 700 760)	0.00	-	-
Inflammatory myopathies‡§	16 (1 700 750)	0.01 (0.01 to 0.02)	-	-
Systemic lupus erythematosus‡§	≤5 (1 700 754)	0.00	-	-
Autoimmune (idiopathic) thrombocytopenic purpura‡§	164 (1 700 430)	0.10 (0.08 to 0.11)	-	-
Juvenile pernicious (megaloblastic) anaemia‡§	9 (1 700 741)	0.01 (0.00 to 0.01)	-	-
Childhood vitiligo‡	206 (1 700 390)	0.12 (0.11 to 0.14)	1.13 (0.36 to 3.52)	0.84
**Infections and inflammation**				
Wheeze	49 079 (1 576 600)	31.13 (30.85 to 31.41)	1.00 (0.93 to 1.06)	0.87
Upper respiratory tract infections‡	314 783 (823 002)	382.48 (381.15 to 383.82)	0.99 (0.97 to 1.02)	0.47
Lower respiratory tract infections (excluding bronchiolitis)‡	81 784 (1 495 270)	54.70 (54.32 to 55.07)	1.04 (0.99 to 1.10)	0.14
Bronchiolitis‡	33 133 (1 604 809)	20.65 (20.42 to 20.87)	1.05 (0.98 to 1.12)	0.20
Gastroenteritis‡	56 403 (1 550 710)	36.37 (36.07 to 36.67)	1.03 (0.97 to 1.10)	0.37
Inflammatory bowel disease§	10 (1 700 743)	0.01 (0.00 to 0.01)	-	-
Urinary tract infection‡	24 416 (1 655 549)	14.75 (14.56 to 14.93)	1.00 (0.90 to 1.11)	0.99
Antibiotic prescribing in primary care‡	336 893 (783 562)	429.95 (428.50 to 431.41)	1.03 (1.00 to 1.06)	0.02
**Other immune system related conditions**				
Leukaemia‡§	116 (1 700 571)	0.07 (0.06 to 0.08)	-	-
**Neurodevelopmental conditions**				
Cerebral palsy	375 (1 699 874)	0.22 (0.20 to 0.24)	1.65 (0.79 to 3.45)	0.18
Autism spectrum disorder‡	2021 (1 698 425)	1.19 (1.14 to 1.24)	1.14 (0.77 to 1.67)	0.52
Attention deficit hyperactivity disorder‡	436 (1 700 183)	0.26 (0.23 to 0.28)	2.53 (1.05 to 6.12)	0.04
**Less specific measures of child health**				
Colic‡	20 949 (1 633 464)	12.82 (12.65 to 13.00)	1.06 (0.96 to 1.16)	0.26
Failure to thrive‡	4018 (1 688 880)	2.38 (2.31 to 2.45)	1.08 (0.87 to 1.34)	0.47
**Healthcare utilisation in children**				
Consultations recorded in primary care (in first 12 months)‡	6 688 462 (513 518)	13 024.80 (13 014.92 to 13 034.66)	1.02 (1.01 to 1.02)	<0.001

THIN=The Health Improvement Network; CPRD=Clinical Practice Research Datalink; CI=confidence interval.

* Rates are not directly comparable to rates in overall 0-4 year old population as they differ by age, and the maximum age that the children born in 2015, 2016, 2017 and 2018 were followed up to was 4, 3, 2 years and 1 year, respectively.

† Adjusted for child’s age, year of delivery, and delivery type. The model incorporates a probability that each mother received pre-incision antibiotics based on national policy uptake rates in the year of delivery.

‡ Exploratory outcomes.

§ Too few cases (<200) in THIN and CPRD dataset to fit the model.

We also found no evidence of a significant difference in the risk of the other allergic and allergy related conditions, autoimmune health conditions, infections, and less specific child health measures (ie, colic and failure to thrive). Some of the health conditions, including many autoimmune and neurodevelopmental conditions, were rare in children younger than 5 years and therefore a high level of uncertainty surrounds the risk estimates.

A small 3% relative increase (1.03, 1.00 to 1.06) was observed in the likelihood of children being prescribed antibiotics in primary care, which was statistically significant, with a high rate of antibiotic prescribing in early childhood (table 2). We also observed a small 2% increase (1.02, 1.01 to 1.02) in the total number of healthcare consultations for any reason recorded in primary care in the first 12 months after birth.

For maternal outcomes, the pre-incision antibiotic policy was associated with a 30% reduced risk of the composite outcome of maternal infectious morbidity (0.70, 0.63 to 0.77), principally related to a reduction in the risk of wound infections after caesarean section (table 3).

**Table 3:** Number of deliveries with different maternal infectious outcomes recorded in primary care (THIN and CPRD) dataset in six week postpartum period, percentage by delivery type, and relative risk associated with pre-incision versus post-cord clamping prophylactic antibiotics

Outcomes	No (%) of outcomes by delivery type*			Pre-incision *v* post-cord clamping antibiotics (based on national policy uptake %)			
	Caesarean section (n=144 861)	Vaginal delivery (n=371 084)		Incidence rate ratio (95% CI)†	P value	Incidence rate ratio (95% CI)‡	P value
Composite infectious morbidity§	12 466 (8.61)	5303 (1.43)		0.67 (0.56 to 0.79)	<0.001	0.70 (0.63 to 0.77)	<0.001
Endometritis or endomyometritis	617 (0.43)	1269 (0.34)		0.63 (0.26 to 1.53)	0.31	0.98 (0.72 to 1.33)	0.88
Wound infection	11 717 (8.09)	3800 (1.02)		0.64 (0.53 to 0.77)	<0.001	0.62 (0.55 to 0.69)	<0.001
Urinary tract infection, cystitis, pyelonephritis	2551 (1.76)	4935 (1.33)		0.64 (0.43 to 0.97)	0.04	1.01 (0.87 to 1.17)	0.93
Maternal sepsis	212 (0.15)	254 (0.07)		11.55 (3.66 to 34.41)	<0.001	1.40 (0.85 to 2.31)	0.19
Pelvic abscess¶	≤5 (0.00)	7 (0.00)		-	-	-	-
Antibiotics prescription during six weeks post partum	39 000 (26.92)	59 138 (15.94)		0.94 (0.85 to 1.03)	0.17	0.89 (0.85 to 0.92)	<0.001

THIN=The Health Improvement Network; CPRD=Clinical Practice Research Datalink; CI=confidence interval.

* Numbers and percentages describe overall frequency of outcomes in caesarean section and vaginal delivery groups and do not include a comparison of pre-incision versus post-cord clamping prophylactic antibiotics.

† Adjusted for year of delivery.

‡ Adjusted for year of delivery and delivery type.

§ Composite infectious morbidity includes wound infection, endometritis or endomyometritis, maternal sepsis, and pelvic abscess.

¶ Too few cases (<200) in THIN and CPRD dataset to fit model.

Sensitivity analyses for asthma produced fairly similar results, with incidence rate ratios generally slightly lower than in the primary analysis (see supplementary table S2). Sensitivity analyses for eczema also produced similar results to the primary analysis (see supplementary table S3). The effect sizes for outcomes with unexpected statistically significant results were lower in sensitivity analyses (see supplementary table S4), including for attention deficit hyperactivity disorder, showing a high degree of imprecision for the risk estimate of this outcome and suggesting that these results could be spurious.

Exploratory subgroup analyses of primary outcomes by emergency and elective caesarean section also did not suggest a statistically significant differential effect of prophylactic antibiotic timing (see supplementary table S5).

### Hospital admissions

Table 4 summarises the numbers of children with a health outcome of interest resulting in hospital admission recorded in the HES dataset, total number of years of follow-up, and overall rate per 1000 person years. We observed a 5% increase in first hospital admissions for asthma (incidence rate ratio 1.05, 95% confidence interval 0.99 to 1.11) and a 4% decrease in admissions for eczema (0.96, 0.71 to 1.29) in children exposed to pre-incision prophylactic antibiotics compared with children not exposed. These differences were not statistically significant.

**Table 4:** Number of children with first hospital admissions by outcome of interest recorded in HES database, overall rate per 1000 person years, and relative risk associated with pre-incision versus post-cord clamping prophylactic antibiotics

Outcomes	Total No (follow-up time in person years)	Overall rate per 1000 person years (95% CI)*	Pre-incision *v* post-clamping antibiotics† (based on year of policy change in each hospital)	
			Incidence rate ratio (95% CI)	P value
**Primary outcomes**				
Asthma	30 274 (18 137 690)	1.67 (1.65 to 1.69)	1.05 (0.99 to 1.11)	0.12
Eczema	1154 (18 137 690)	0.06 (0.06 to 0.07)	0.96 (0.71 to 1.29)	0.77
**Other allergic and allergy related conditions**				
Anaphylaxis‡	1665 (18 137 690)	0.09 (0.09 to 0.10)	1.01 (0.82 to 1.25)	0.91
**Autoimmune diseases**				
Type 1 diabetes‡	2445 (18 137 690)	0.13 (0.13 to 0.14)	0.99 (0.82 to 1.20)	0.94
Coeliac disease‡§	178 (18 137 690)	0.01 (0.00 to 0.01)	-	-
Juvenile idiopathic arthritis‡	297 (18 137 690)	0.02 (0.01 to 0.02)	0.97 (0.54 to 1.74)	0.92
Scleroderma or systemic sclerosis‡§	≤5 (18 137 690)	0.00	-	-
Inflammatory myopathies‡§	26 (18 137 690)	0.00	-	-
Systemic lupus erythematosus‡§	≤5 (18 137 690)	0.00	-	-
Autoimmune (idiopathic) thrombocytopenic purpura‡	1506 (18 137 690)	0.08 (0.08 to 0.09)	1.11 (0.88 to 1.39)	0.37
Juvenile pernicious (megaloblastic) anaemia‡§	≤5 (18 137 690)	0.00	-	-
**Infections and inflammation**				
Early onset neonatal sepsis (<3 days of birth)¶	3336 (3 945 351)	0.85 (0.82 to 0.87)	0.75 (0.65 to 0.87)	<0.001
Late onset neonatal sepsis (4-28 days after birth)¶	7226 (3 945 351)	1.83 (1.79 to 1.87)	0.88 (0.80 to 0.97)	0.008
Other sepsis (>29 days after birth)‡	15 182 (17 835 240)	0.85 (0.84 to 0.86)	0.98 (0.91 to 1.04)	0.48
Lower respiratory tract infections (excluding bronchiolitis)‡	128 399 (18 137 690)	7.08 (7.04 to 7.12)	0.99 (0.96 to 1.01)	0.19
Bronchiolitis‡	153 411 (18 137 690)	8.46 (8.42 to 8.50)	1.01 (0.98 to 1.03)	0.64
Gastroenteritis‡	145 293 (18 137 690)	8.01 (7.97 to 8.05)	1.02 (1.00 to 1.05)	<0.001
Inflammatory bowel disease§	87 (18 137 690)	0.00	-	-
Urinary tract infection‡	41 676 (18 137 690)	2.30 (2.28 to 2.32)	1.00 (0.95 to 1.04)	0.86
**Other immune system related conditions**				
Necrotising enterocolitis¶	1178 (3 945 351)	0.30 (0.28 to 0.32)	1.16 (0.95 to 1.42)	0.16
Leukaemia‡	1094 (18 137 690)	0.06 (0.06 to 0.06)	0.97 (0.74 to 1.29)	0.86
**Healthcare utilisation in children**				
Any hospital admission‡	1 459 776 (18 137 690)	80.48 (80.35 to 80.61)	1.01 (1.01 to 1.02)	<0.001

HES=Hospital Episode Statistics; CI=confidence interval.

* Rates are not directly comparable to rates in overall 0-4 year old population, as they differ by age, and maximum age that children born in 2015, 2016, and 2017 were followed up to was 4, 3 and 2 years, respectively.

^†^ Adjusted for year of delivery and mode of birth. Model only includes data from hospitals for which year of antibiotic prescribing policy change is known.

‡ Exploratory outcomes.

§ Too few cases (<200 in HES dataset) to fit model.

¶ As these conditions occurred in the neonatal period, rates were calculated per 1000 births.

Also, we found no evidence of a statistically significant increase in risk of any of the other allergic, autoimmune, or immune system related conditions. We observed a small 2% increase in the risk of hospital admissions for gastroenteritis (1.02, 1.00 to 1.05), and a small 1% relative increase in the risk of a hospital admission for any reason (1.01, 1.01 to 1.02), which may not be clinically significant. Results also suggested a 25% relative reduction in risk of early onset neonatal sepsis (0.75, 0.65 to 0.87) and 12% reduction for late onset neonatal sepsis (0.88, 0.80 to 0.97) favouring pre-incision antibiotics.

For maternal outcomes recorded in HES, the pre-incision antibiotic policy was associated with a 16% reduced risk of maternal infectious morbidity (0.84, 0.81 to 0.87), principally related to the reduction in the risk of wound infections after caesarean section (table 5). The duration of hospital stay was also slightly shorter. We observed that the overall incidence of maternal sepsis recorded in secondary care had increased over time irrespective of delivery type during the study period; however, for caesarean section the relative risk of sepsis associated with the pre-incision antibiotics policy increased by 15% (1.15, 1.05 to 1.26).

**Table 5:** Number of deliveries with different maternal infectious outcomes recorded in HES database in six week postpartum period, percentage by delivery type, and relative risk associated with pre-incision versus post-cord clamping prophylactic antibiotics

Outcomes	No (%) of outcomes by delivery mode*			Pre-incision *v* post-clamping antibiotics (based on year of policy change in each hospital)			
	Caesarean section (n=1 001 598)	Vaginal delivery (n=2 943 753)		Incidence rate ratio (95% CI)†	P value	Incidence rate ratio (95% CI)‡	P value
Composite infectious morbidity§	18 934 (1.89)	9112 (0.31)		0.98 (0.94 to 1.02)	0.31	0.84 (0.81 to 0.87)	<0.001
Endometritis or endomyometritis¶	41 (0.00)	40 (0.00)		-	-	-	-
Wound infection	16 185 (1.62)	6701 (0.23)		0.94 (0.90 to 0.98)	0.006	0.84 (0.81 to 0.87)	<0.001
Urinary tract infection, cystitis, pyelonephritis	3148 (0.31)	5526 (0.19)		1.14 (1.03 to 1.26)	0.01	1.04 (0.96 to 1.14)	0.32
Sepsis	2878 (0.29)	2389 (0.08)		1.22 (1.09 to 1.36)	<0.001	1.15 (1.05 to 1.26)	0.003
Maternal deaths¶**	≤5 (0.00)	≤5 (0.00)		-	-	-	-
Median (IQR) length of stay (days)	3 (2 to 4)	1 (1 to 2)		−0.004 (−0.02 to 0.01)††	0.66	−0.23 (0.24 to −0.21)††	<0.001

HES=Hospital Episode Statistics; CI=confidence interval; IQR=interquartile range.

* Values describe overall frequency of outcomes in delivery groups and do not include a comparison of pre-incision versus post-cord clamping antibiotics.

† Adjusted for year of delivery.

‡ Adjusted for year of delivery and delivery type.

§ Composite of wound infection, endometritis or endomyometritis, or maternal sepsis.

¶ Too few cases (<200) in HES dataset to fit model.

** HES dataset did not contain information on cause of death. Deaths reported here include those that also involved a diagnosis of any of the health conditions in the composite infectious morbidity outcome in the maternal record during the postpartum period.

†† Mean difference.

Results of sensitivity analyses were in line with the results of the main analysis (see supplementary tables S6-8), although uncertainty was higher around the point estimates for early onset neonatal sepsis.

The results of exploratory subgroup analyses by emergency and elective caesarean section were similar to the main results and did not suggest a statistically significant differential effect on the primary outcomes (see supplementary table S9). We found no evidence to suggest a differential effect of different antibiotic regimens on risk of asthma related hospital admissions and necrotising enterocolitis. Hospital admissions for eczema were rare and more evidence would be required to confirm the observed associations between co-amoxiclav and risk of hospital admissions, as the degree of uncertainty around the estimates was high (see supplementary table S10).

## Discussion

In this large population based study, we did not find evidence for an association between a change in policy to recommend prophylactic antibiotics before incision for caesarean section and risk of asthma, eczema, and other allergic and allergy related conditions. We also did not find convincing evidence for an increased risk of other conditions, including autoimmune diseases, immune system related conditions, infections, and neurodevelopmental conditions.

### Strengths and limitations of this study

Evidence on the impact of a policy to use pre-incision antibiotics on child health is limited. We used two large population-wide mother-baby linked healthcare datasets, allowing us to examine the impact of the pre-incision antibiotic policy on a broad range of health conditions in childhood, and their severity. Our study used a strong design and considered children born vaginally as a control group to adjust for temporal changes, including changes in diagnoses and recording in routine healthcare records over the study period. The study design also allowed us to explore any differences in risk associated with a particular antibiotic regimen and the effect of the timing of antibiotic prophylaxis in emergency and elective caesarean section. Robustness of the findings was tested in a series of sensitivity analyses, exploring the impact of timing of the policy change for prophylactic antibiotics, data recording quality, outcome definition, and independence of observations.

Our study has several limitations. Timing of antibiotic prophylaxis is not available in routine healthcare records, and therefore we could not ascertain exposure to pre-incision antibiotics at an individual level. In our main analyses, we relied on the results from the survey of clinical directors for maternity care to describe the uptake of the pre-incision antibiotic policy across the UK over time. This could have introduced recall bias as the clinical directors’ recollection of the year of the local policy change may not have been accurate. Also, not all women undergoing caesarean section would have received pre-incision antibiotics after the policy change, although according to the results of our survey, in hospitals that had conducted audits, most (70-100% of women) did receive antibiotics before incision. We undertook sensitivity analysis to assess the robustness of the results depending on the definition of timing of the prophylactic antibiotic policy change, and the results were consistent with the main analysis. Routine healthcare records also do not contain information on other antibiotics administered during delivery, but as they are offered to a relatively smaller proportion of women (eg, to prevent group B streptococcal infection in babies), they usually are not broad spectrum antibiotics and would not be expected to have the same impact on gut microbiota as prophylactic antibiotics recommended for caesarean section. Some of the health conditions are more difficult to diagnose in early childhood and are based on symptoms. For asthma, for example, objective tests are recommended in children from 5 years of age.^48^ Validated case definitions for routine healthcare records, when such exist, are also mostly derived from populations including older children or adults.^41^ ^49^ Finally, it is known that type of delivery is associated with prevalence of health outcomes, and so our control group of vaginally delivered children differs from the caesarean section group both before and after exposure to antibiotics. Our use of this vaginally delivered control group was to allow for any temporal trends to be correctly modelled, as not including a control group may have led to unidentifiable time or exposure effects. Nevertheless, this use of a control group could add additional confounding to our analysis. Reassuringly we did not observe noticeable differential changes over time in the study covariates by delivery type, although breastfeeding data were only available to be assessed by delivery type over time at a national level.

Many health conditions of interest in this study were exploratory because of weak existing evidence for an association between early exposure to antibiotics, gut microbiota, and these health outcomes. Some diseases were rare in our study population, precluding calculation of meaningful risk estimates. However, some conditions, such as gastroenteritis, are common, and healthcare utilisation is high in early childhood, and we were therefore able to detect small (1-3%) relative differences for these common events, which are unlikely to be clinically significant and may potentially be explained by residual confounding not fully accounted for by using the vaginal delivery comparator group.

Our finding of a 30% relative reduction in maternal infectious morbidity recorded in primary care is comparable to the estimates of a 28% and 43% reduction in the systematic reviews of randomised controlled trials comparing the effectiveness of pre-incision and post-cord clamping prophylactic antibiotics.^4^ ^50^ Replication of this randomised controlled trial evidence for maternal outcomes in the routine data adds credibility to our findings for childhood outcomes. In our study, this decrease was mainly as a result of a reduction in wound infections. Large uncertainty surrounded the estimates for endometritis and a relative increase of 15% for maternal sepsis recorded in secondary care, which is likely to be an artefact as any major surgical procedure raises the suspicion of sepsis leading to an increased rate of diagnosis. This was an observational study using routine healthcare records, and the incidence of recorded maternal sepsis had increased for both caesarean section and vaginal delivery, which could result from an increase in diagnosis, prevention, and treatment of sepsis over the study period. Evidence from randomised controlled trials suggests that prophylactic antibiotics can reduce serious maternal infectious complications by 69%.^3^

### Comparison with other studies

Evidence from systematic reviews of randomised controlled trials on the impact of preoperative antibiotics focusing on maternal infectious morbidity, suggests that administering antibiotics before incision may also be associated with a 23-24% reduction in neonatal sepsis; however, these studies were not sufficiently powered to detect significant differences in this outcome.^4^ ^50^ Although we observed a statistically significant reduction of 25% and 12% in early and late neonatal sepsis, respectively, these findings should be interpreted with caution as sensitivity analyses suggested large uncertainty around these estimates. The incidence of neonatal sepsis recorded in routine healthcare records had increased over time in babies, irrespective of delivery type. The observed relative reduction associated with pre-incision antibiotics could be an artefact, as our study is likely to have only captured culture confirmed sepsis, as culture results may be negative if babies were exposed to antibiotics immediately before birth even if they developed signs of neonatal sepsis.

Given that we explored more than 50 different outcomes across both primary and secondary care datasets, and undertook multiple tests, some of the results we observed may have been due to chance. For example, evidence on the association between antibiotics in early childhood and attention deficit hyperactivity disorder is conflicting, with studies suggesting that any observed associations may be fully or partially explained by familial confounding.^51^ ^52^ ^53^ The numbers of children with a diagnosis of attention deficit hyperactivity disorder were low in this study, as this condition is more commonly diagnosed in older children.^12^ Therefore, incidence varied widely between the study years, and there was large uncertainty around the estimates.

In our exploratory subgroup analysis we did not find evidence for a statistically significantly increased risk with any of the antibiotics used (cefuroxime, metronidazole and co-amoxiclav) for the primary outcomes (asthma and eczema) and for necrotising enterocolitis; however, the degree of uncertainty around these estimates was high and we only examined the overall association and did not assess the risk in any specific subgroups of babies who have been shown to be at higher risk of necrotising enterocolitis.^54^ ^55^

### Conclusions

Our study suggests that a policy to administer prophylactic antibiotics before incision for caesarean section is not associated with risk of developing asthma, eczema, and other allergy related conditions in early childhood. The findings therefore provide supportive evidence for the discussion about timing of prophylactic antibiotics with women delivering by caesarean section^56^ and the recommendation to use antibiotics before incision to reduce maternal infections in the postpartum period. The results of this study might not be generalisable to settings that use different prophylactic antibiotic regimens. The impact of antibiotic exposure around the time of birth is also not generalisable to babies born vaginally as they are less likely to be colonised by opportunistic pathogenic microorganisms than babies born by caesarean section.^9^ Further studies to confirm the findings in other populations and older children are warranted.

**What is already known on this topic**

Prophylactic antibiotics administered before caesarean section have been shown to be more effective at reducing the overall risk of maternal postpartum infections than antibiotics used after cord clamping
Babies are exposed to antibiotics through the placenta when prophylactic antibiotics are administered preoperatively
Intrapartum antibiotics alter the development of the infant’s gut microbiota, but no previous studies have assessed the long term impact on child health of pre-incision antibiotics for caesarean section

**What this study adds**

The introduction of a policy to administer prophylactic antibiotics before incision for caesarean section was not associated with an increase in risk of asthma, eczema, and other allergy and immune related diseases in early childhood

## Data Availability

Data for this study were derived from Clinical Practice Research Datalink GOLD and The Health Improvement Network primary care and secondary care linked databases obtained under license from the UK Medicines and Healthcare Products Regulatory Agency and IQVIA World Publications. A separate secondary care database was derived from Hospital Episode Statistics data obtained under licence from the Health and Social Care Information Centre (NHS Digital). Data for similar cohorts can be requested from the UK Medicines and Healthcare Products Regulatory Agency, IQVIA World Publications, and NHS Digital subject to protocol approval and license agreements.

## Web extra

Extra material supplied by authors

## Notes

Contributors: DS, PB, JJD, KN, BHW, MS, and RH conceived and designed the study. GMR, KG, KN, RT, and NJA acquired, linked, and cleaned the data. MS and RH provided patient and public involvement advice. JM and JJD were responsible for statistical analysis. BHW, PB, KN, JSC, and KO provided clinical input, including defining the study outcomes. IMH contributed to the literature review. All authors interpreted the results, prepared the manuscript, and approved the final version. DS is the guarantor. The corresponding author attests that all listed authors meet authorship criteria and that no others meeting the criteria have been omitted.

The lead author (DS) affirms that the manuscript is an honest, accurate, and transparent account of the study being reported; that no important aspects of the study have been omitted; any discrepancies from the study as planned (and registered) have been explained.

Dissemination to participants and related patient and public communities: It is not possible to disseminate results to study participants directly as we used anonymised healthcare records in this study. We will disseminate the study results through our website (www.birmingham.ac.uk/research/applied-health/research/health-informatics/long-term-impact-of-pre-incision-antibiotics-on-babies-born-by-caesarean-section.aspx) and use ARC West Midlands networks, which include patient organisations, as a platform for wider dissemination.

Provenance and peer review: Not commissioned; externally peer reviewed.
