# Supplementary material for: Long term impact of prophylactic antibiotic use before incision versus after cord clamping on children born by caesarean section: longitudinal study of UK electronic health records
Source: BMJ. 2022 May 18;377:e069704. doi: 10.1136/bmj-2021-069704 (PMC9112858; doi:10.1136/bmj-2021-069704)
Supplement: Supplementary file 1 — Supplementary material: additional information, tables, and figures [file sumd069704.ww.pdf]

# Supplementary material

## Table of Contents

**Supplementary material** ... 1

Further details on healthcare data sources and linkage ... 2

Further details on statistical analyses ... 3

Statistical analyses for outcomes recorded in primary care ... 3

Statistical analyses for outcomes recorded in HES ... 4

Further information on the national survey of hospital prophylactic antibiotic administration policies ... 6

Figure S1. National cumulative uptake of pre-incision antibiotic policy for caesarean section over time ... 6

Figure S2. Flow diagram of study population selection in THIN-CPRD dataset ... 7

Figure S3. Flow diagram of study population selection in HES dataset ... 8

Table S1. The list of secondary outcomes and datasets analysed for each outcome ... 8

Results of sensitivity analyses in THIN-CPRD ... 10

Results of sensitivity analyses in HES ... 13

Results of exploratory subgroup analyses ... 14

Covariate graphs (Figures S4-24) ... 15

Maternal and household characteristics ... 15

Child characteristics ... 22

Rates of study outcomes over time by mode of delivery (Tables S11-12 and Figures S25-74) ... 25

Child outcomes in THIN-CPRD dataset ... 26

Maternal outcomes in THIN-CPRD dataset ... 38

Child outcomes in HES dataset ... 40

Maternal outcomes in HES dataset ... 46

## Further details on healthcare data sources and linkage

Mother and baby records in the CPRD GOLD dataset provided to us by the CPRD Centre of the Medicines and Healthcare products Regulatory Agency (MHRA) were already linked.[1] Linkage of anonymised records is possible due to unique family identification numbers based on residence, child's birth year and month, delivery, pregnancy related and postnatal information.[2,3] We adapted a similar strategy for linking mother and baby records in THIN. For each delivery during the study period in the mother's healthcare record, we checked if there was a baby and a mother with the same family identification number and if the delivery's year and month matched the baby's year and month of birth. In both THIN and CPRD, children had to be registered with the mother's general practice within the first year of life to be included in the study dataset.

Some general practices contribute data to both THIN and CPRD GOLD databases. To identify patient records from these practices, we adapted previously published algorithms using practice-related, patient registration, demographic and medical information to ensure that duplicate records were not included in our final THIN-CPRD dataset.[4,5]

As there is no common identifier to link maternal and baby birth records in HES, we adapted a previously validated algorithm for linking maternal and baby data in this dataset.[6] We first deterministically linked maternal and baby records if they matched on the code for the general practice, maternal age, birthweight, gestation, birth order and baby's sex. We allowed for missingness in up to two of these five variables but disallowed the match if any of the five variables, which were not empty, did not agree. The remaining maternal delivery records were linked probabilistically by comparing them to birth records on 21 variables. Each variable was given a positive match weight, if there was a match between the mother's and baby's record, and a negative weight if there was a mismatch. We applied previously published weights and matching threshold to decide which matches were probable.[6]

---

[1] Williams R. CPRD Mother Baby Link Documentation. Medicines & Healthcare products Regulatory Agency; 2017.

[2] Cea-Soriano L, Garcia Rodriguez LA, Fernandez Cantero O, Hernandez-Diaz S. Challenges of using primary care electronic medical records in the UK to study medications in pregnancy. Pharmacoepidemiol Drug Saf. 2013;22(9):977-85.

[3] Charlton R, Snowball J, Sammon C, de Vries C. The Clinical Practice Research Datalink for drug safety in pregnancy research: an overview. Therapie. 2014;69(1):83-9.

[4] Petersen I, McCrea R, Sammon C, Osborn D, Evans S, Cowen P, et al. Risks and benefits of psychotropic medication in pregnancy: cohort studies based on UK electronic primary care health records. Health Technol Assess. 2016;20(23).

[5] Cai B, Xu W, Bortnichak E, Watson DJ. An algorithm to identify medical practices common to both the General Practice Research Database and The Health Improvement Network database. Pharmacoepidemiol Drug Saf. 2012;21(7):770-4.

[6] Harron K, Gilbert R, Cromwell D, van der Meulen J. Linking Data for Mothers and Babies in De-Identified Electronic Health Data. PLoS One. 2016;11(10):e0164667.

## Further details on statistical analyses

### Statistical analyses for outcomes recorded in primary care

In the THIN-CPRD dataset analysis, the primary outcomes and dichotomous secondary outcomes in children are summarised using the number of children with a record of the outcome of interest and the length of time at risk, which was estimated as the time from birth to the earliest of: the child's fifth birthday, the date when the child was diagnosed with the outcome of interest for the first time, the date the child was lost to follow-up (for example, due to moving from the general practice), or the study end date (31 December 2018). The rate of each outcome was calculated as the ratio of the number of events and exposure time in person years, multiplied by 1,000. Secondary outcomes that were counts are summarised using the total number of events and the person years at risk. Maternal outcomes are summarised as the percentage of total births stratified by delivery mode.

A Poisson regression model was fitted to the data with an offset for the follow-up time. The model using THIN-CPRD data included terms for the year of birth, age (year of life), their interaction, delivery mode (to allow for differential risk of the outcome in those who had CS compared to VD), and antibiotic timing (to allow for differential risk in those receiving pre-incision rather than post-cord clamping prophylactic antibiotics). Whereas in a simple before-after time series design this variable would be a zero-one variable, we used the probability of antibiotic use before skin incision in each year assessed through our national survey on the timing of antibiotic prophylaxis for CS, adjusting for misclassification. We present the incident rate ratios (IRRs) and the 95% confidence intervals as the rate of outcomes in those who would have been exposed to pre-incision prophylactic antibiotics to the rate of outcomes in those in the unexposed group (receiving antibiotic prophylaxis after cord clamping). For outcomes that were very rare in our study population (with fewer than 200 individuals diagnosed over the whole study period), we present summary statistics only.

#### *Sensitivity and subgroup analyses*

We conducted a series of different sensitivity analyses to assess the robustness of the results: sensitivity to outcome definition, definition of timing of the prophylactic antibiotic policy change, data recording quality and random effect. Sensitivity analyses were done for the two primary outcomes and secondary outcomes where results suggested a statistically significant association with the timing of antibiotic prophylaxis and the association was unexpected.

In the sensitivity analysis to outcome definition, we investigated the impact of prophylactic antibiotic timing on severity of asthma and eczema based on prescribing information indicative of moderate or severe asthma and eczema. We also explored several alternative definitions of asthma and eczema, including asthma diagnosed in children between the third and the fifth year of life, asthma definition based on Read codes in the Quality and

Outcomes Framework,[7] eczema diagnosis based on Read codes in children between the first and the fifth birthday and at least two related prescriptions within 90 days before or 365 days after the eczema Read code record, and eczema diagnosis based on Read codes only.

In the sensitivity analysis for the definition of timing of the prophylactic antibiotic policy change, we compared births (between 2006 and 2010) before the NICE guidance recommendation to administer antibiotic prophylaxis for CS prior to skin incision to the births in the years when over half of all hospitals had introduced the pre-incision antibiotic policy (between 2013 and 2018). Children delivered by CS were classified as either not having received pre-incision antibiotics (between 2006 and 2010) or exposed to pre-incision antibiotics (between 2013 and 2018).

In the sensitivity analysis evaluating the impact of data recording quality, THIN-CPRD database was restricted to mother-baby pairs with linked secondary care data as the most accurate source for mode of birth.

In the sensitivity analysis examining non-independence of observations, we fitted a mixed-effects Poisson model including a random effect for general practice.

Finally, for the primary outcomes we also undertook exploratory subgroup analysis by the type of CS (elective and emergency). For this analysis, we report the IRR for prophylactic antibiotic timing in the elective CS group, emergency CS group and the p-value for the interaction between them indicating the strength of evidence of a differential effect of antibiotic prophylaxis timing based on CS type.

### Statistical analyses for outcomes recorded in HES

In the HES dataset analysis, most primary and dichotomous secondary outcomes are summarised using the number of children admitted to hospital at least once and the length of time at risk, which was estimated as the time from birth to the earliest of: the child's fifth birthday or the study end date (31 March 2019). The rate of each outcome was calculated as the ratio of the number of events and exposure time in person years, multiplied by 1,000. For secondary outcomes with a very short exposure time (neonatal sepsis and necrotising enterocolitis), we present the outcome rate per 1,000 births. Maternal outcomes are summarised the same way as in the THIN-CPRD dataset, apart from hospital length of stay, which was not normally distributed and therefore the median and the inter-quartile range (IQR) is presented.

Like in the THIN-CPRD analysis, a Poisson regression model was fitted to the HES data for all dichotomous outcomes, with an offset for exposure time. It included terms for the year of birth, mode of birth (VD or CS), and prophylactic antibiotic timing, which was categorised as before policy change, year of policy change, and after policy change, for each birth based on the year of policy change in each hospital that responded to the national survey on antibiotic prophylaxis for CS. The comparison between births after the year of the policy

[7] NHS Digital. Quality and Outcomes Framework [Available from: http://content.digital.nhs.uk/qof].

change (assumed to have been exposed to pre-incision antibiotics) and births before the year of the policy change (assumed having no exposure to pre-incision antibiotics), gives an estimate of the effect of the policy change in prophylactic antibiotic timing. For continuous outcomes (maternal length of hospital stay), we fitted a linear regression model and present the difference in mean length of stay between deliveries when pre-incision antibiotic policy was in place and deliveries during post-cord clamping policy.

### *Sensitivity and subgroup analyses*

We conducted several sensitivity analyses: sensitivity to definition of timing of the prophylactic antibiotic policy change, random effect, and exploratory discordant sibling analysis.

In sensitivity to definition of timing of antibiotic policy change, we used the full mother-baby linked HES dataset, not limiting it to the births that could be linked to the hospitals who had reported the year when they implemented pre-incision antibiotic policy, and included the overall probability of pre-incision antibiotic use in each year estimated from our national survey on the timing of antibiotic prophylaxis using responses from hospitals in England.

In the sensitivity analysis examining non-independence of observations, we fitted a mixed-effects Poisson model including a random effect for hospital.

We also conducted an exploratory discordant sibling analysis attempting to control further for family-related genetic and environmental factors. For this analysis, we used a subset of data which included mothers who gave birth at least twice during the study period using the same delivery mode (CS or VD), including having one birth before and one birth after the year of the hospital prophylactic antibiotic policy change.

In addition to the exploratory subgroup analysis by CS type, for outcomes recorded in HES we also performed subgroup analysis by the most common antibiotic regimen type used (co-amoxiclav alone, cefuroxime alone, cefuroxime and metronidazole). Antibiotic regimen analysis was restricted to data from those hospitals who reported the use of the same antibiotics before and after the change in the timing of antibiotic prophylaxis. A Poisson model was fitted separately for each antibiotic regimen. This exploratory analysis was undertaken for the primary outcomes and necrotising enterocolitis due to previously reported increased risk of this disease in newborns of mothers who had received co-amoxiclav.[8]

---

[8] Kenyon SL, Taylor DJ, Tarnow-Mordi W, Group OC. Broad-spectrum antibiotics for preterm, prelabour rupture of fetal membranes: the ORACLE I randomised trial. ORACLE Collaborative Group. Lancet. 2001;357(9261):979-88.

## Further information on the national survey of hospital prophylactic antibiotic administration policies

The survey on prophylactic antibiotic administration policies during CS was designed using REDCap in 2017 and a survey link was sent to all clinical directors for maternity units performing caesarean sections in the UK. Non-responders were followed up using e-mail, telephone and at professional conferences, and were also offered an option to complete the survey in MS Word.

An overall survey response rate of 75.7% (143/189) was achieved with hospitals responding whether they had implemented the pre-incision antibiotic policy in their hospital. A small proportion, 4.2% (6/143) of the hospitals that responded to the survey had not implemented the pre-incision antibiotics policy. Of the hospitals that had implemented the policy, 86.9% (119/137) indicated in which year they had implemented the pre-incision antibiotic policy. Of these respondents, 9.2% (11/119) did not specify the exact year of the policy change, but had entered a free text comment saying, for example, before which year the policy was changed, such as "prior to 2010". This year was used in the analysis as the year of the policy change. Few hospitals (6) stated that they had implemented the policy before 2010. In the survey, we also asked if the hospital had undertaken a clinical audit regarding the use of prophylactic antibiotics at the time of CS within the last few years and if so, what proportion of women were given prophylactic antibiotics before skin incision. In the seven hospitals who had audited the compliance with their pre-incision antibiotic policy, the proportion of CS deliveries where antibiotics were administered before skin incision varied between 70 to 100%.

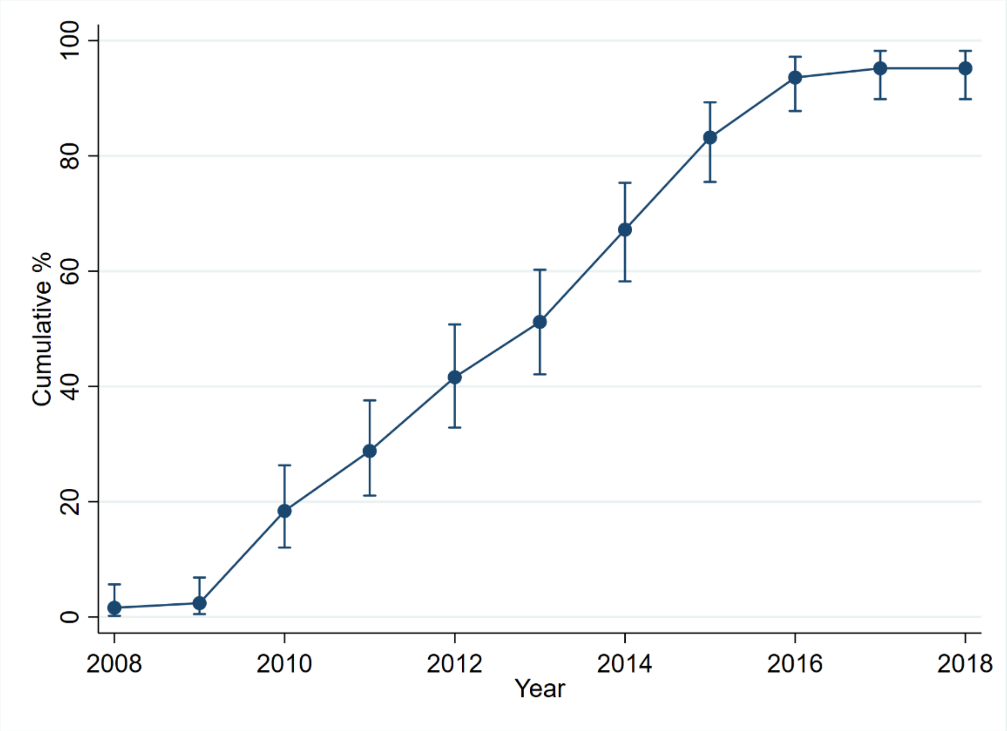


Figure S1. National cumulative uptake (with 95% CIs) of pre-incision antibiotic policy for caesarean section over time (based on responses from 66.1%, 125/189 hospitals in the UK)

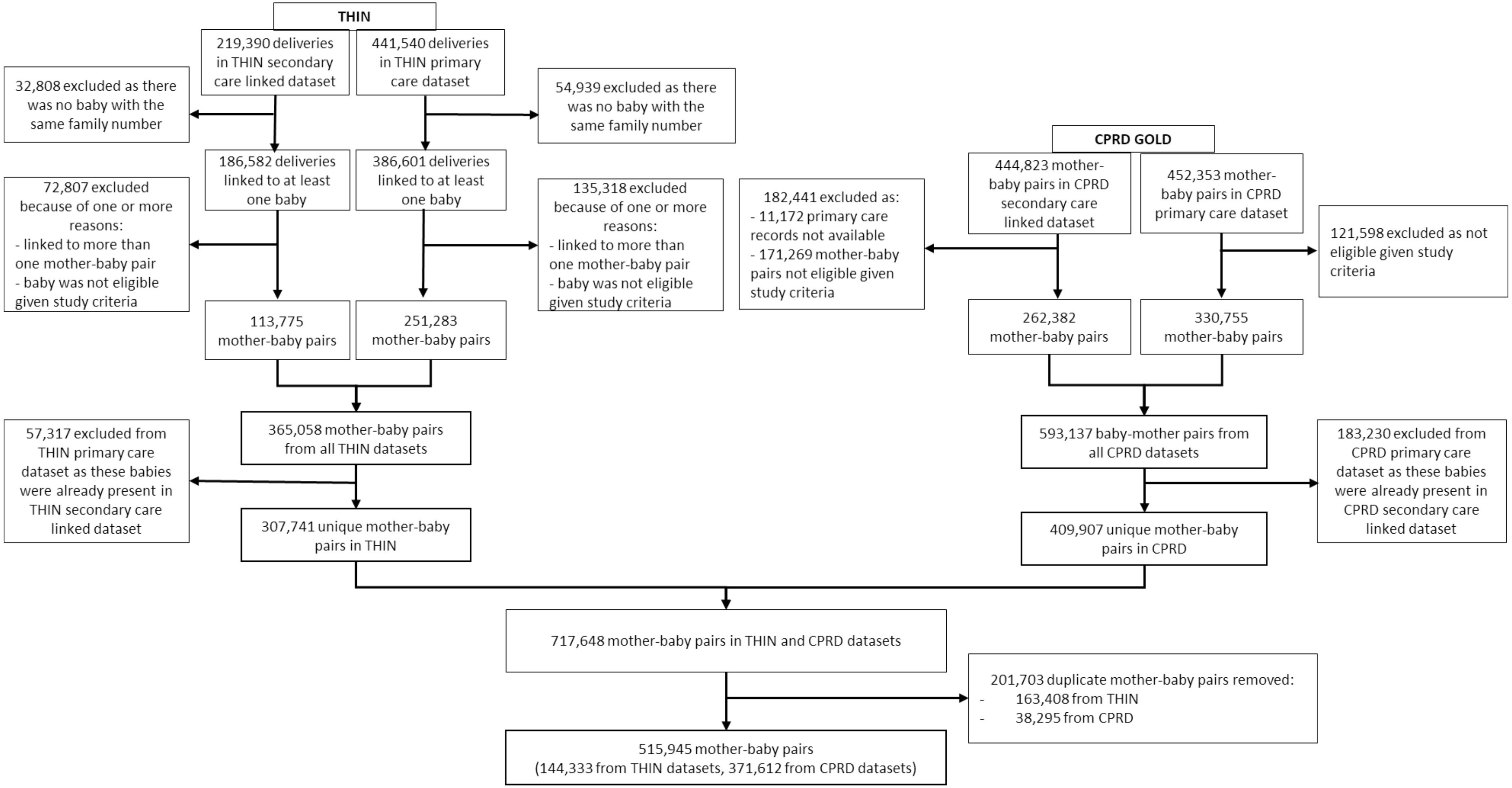


Figure S2. Flow diagram of study population selection in THIN-CPRD dataset

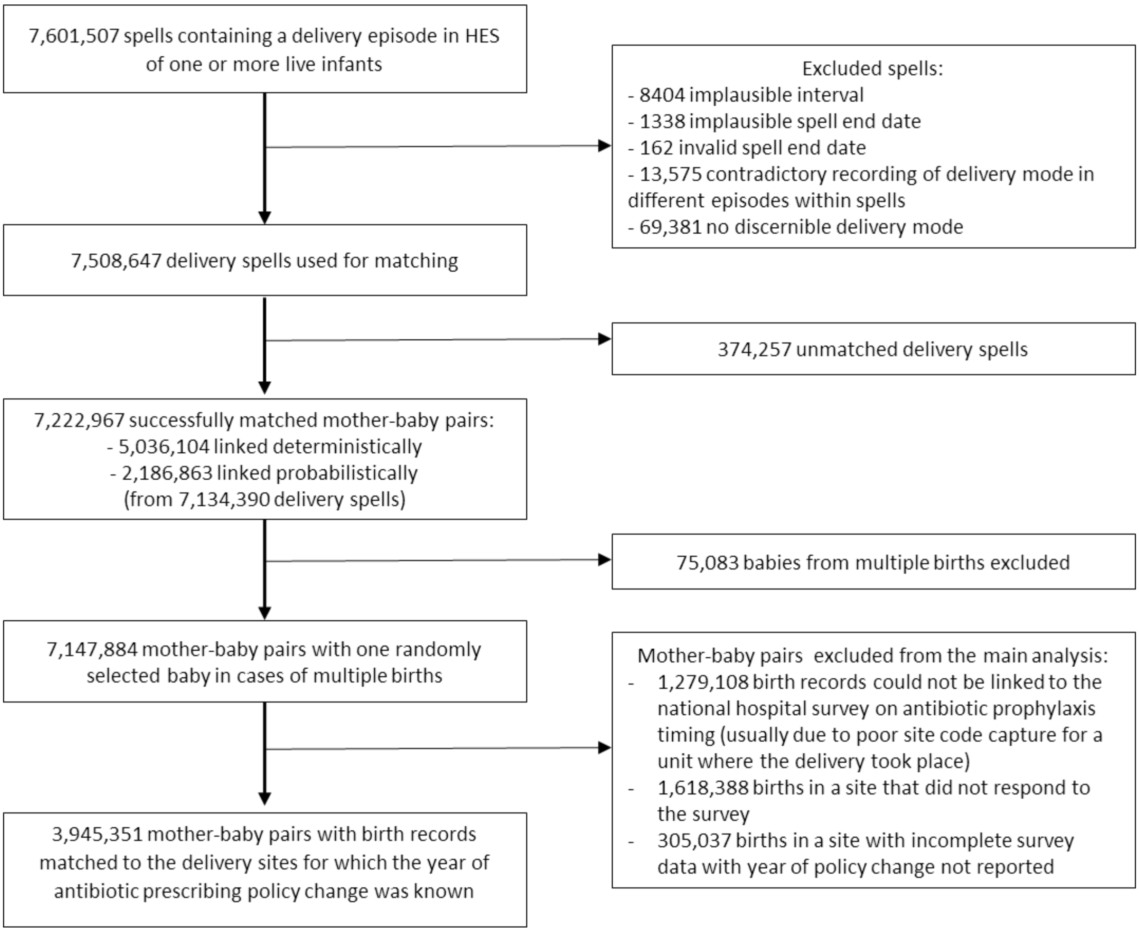


Figure S3. Flow diagram of study population selection in HES dataset

Table S1. The list of secondary outcomes and datasets analysed for each outcome

| **Outcome** | **Datasets analysed** | |
|---|---|---|
| | **THIN-CPRD primary care dataset** | **HES dataset** |
| **Health conditions and symptoms in children** | | |
| Other allergic and allergy-related conditions: | | |
| - Food allergy/intolerance | Yes | |
| - Allergic rhinitis and conjunctivitis | Yes | |
| - 2 or more allergy related diseases (asthma, eczema, food allergy/intolerance, allergic rhinitis and conjunctivitis) | Yes | |
| - Penicillin allergy* | Yes | |
| - Anaphylaxis* | Yes | Yes |
| - High risk of anaphylactic reaction (prescribing of automatic injection devices containing adrenaline)* | Yes | |
| *Autoimmune diseases:* | | |
| - Type 1 diabetes* | Yes | Yes |
| - Coeliac disease* | Yes | Yes |
| - Juvenile idiopathic arthritis* | Yes | Yes |
| - Scleroderma/systemic sclerosis* | Yes | Yes |

| | | |
|---|---|---|
| - Inflammatory myopathies* | Yes | Yes |
| - Systemic lupus erythematosus (SLE)* | Yes | Yes |
| - Autoimmune (idiopathic) thrombocytopenic purpura (ITP)* | Yes | Yes |
| - Juvenile pernicious (megaloblastic) anaemia* | Yes | Yes |
| - Childhood vitiligo* | Yes | |
| *Infections and inflammation:* | | |
| - Neonatal sepsis (early and late onset) | | Yes |
| - Other sepsis* | | Yes |
| - Wheeze | Yes | |
| - Upper respiratory tract infections* | Yes | |
| - Lower respiratory tract infections* | Yes | Yes |
| - Bronchiolitis* | Yes | Yes |
| - Gastroenteritis* | Yes | Yes |
| - Inflammatory bowel disease | Yes | Yes |
| - Urinary tract infections* | Yes | Yes |
| - Antibiotic prescribing* | Yes | |
| *Other immune system-related conditions:* | | |
| - Necrotising enterocolitis | | Yes |
| - Leukaemia* | Yes | Yes |
| *Neurodevelopmental conditions:* | | |
| - Cerebral palsy | Yes | |
| - Autism spectrum disorder* | Yes | |
| - Attention deficit hyperactivity disorder (ADHD)* | Yes | |
| *Less specific measures of child health:* | | |
| - Colic* | Yes | |
| - Failure to thrive* | Yes | |
| **Healthcare utilisation in children** | | |
| - Consultations recorded in primary care (in the first 12 months)* | Yes | |
| - Any hospital admission* | | Yes |
| **Maternal outcomes** (up to six weeks post-partum) | | |
| - Composite infectious morbidity (wound infection, endometritis/endomyometritis, pelvic abscess†, maternal sepsis, maternal death‡) | Yes | Yes |
| - Endometritis/endomyometritis | Yes | Yes |
| - Wound infection | Yes | Yes |
| - Urinary tract infection/cystitis/pyelonephritis | Yes | Yes |
| - Sepsis | Yes | Yes |
| - Pelvic abscess† | Yes | |
| - Maternal death*‡ | | Yes |
| - Antibiotic prescribing* | Yes | |
| - Length of hospital stay* | | Yes |

* Exploratory outcome.

† Pelvic abscess was part of the composite infectious morbidity recorded in primary care; there was no specific ICD-10 code to identify pelvic abscess separately in HES dataset.

‡ Only if there was also a record of a diagnosis of any of the health conditions included in the composite infectious morbidity outcome in the post-partum period.

## Results of sensitivity analyses in THIN-CPRD

### Table S2. Number of children with asthma recorded in primary care (THIN-CPRD) database, overall rate per 1,000 person years and the relative risk of pre-incision compared to post-cord clamping prophylactic antibiotics (sensitivity analyses)

| Outcome | Total N (follow-up time in person years) | Overall rate per 1,000 person years | Pre-incision vs post-cord clamping antibiotics* | |
|---|---|---|---|---|
| | | | IRR | 95% CI |
| **Sensitivity to population changes:** | | | | |
| Asthma (2006-2010 vs 2013-2018)[†,‡] | 13,488 (1,339,371) | 10.07 | 0.91 | 0.82 to 1.00 |
| Asthma (secondary care linked dataset)[†,§] | 9,494 (933,557) | 10.17 | 0.89 | 0.72 to 1.10 |
| **Sensitivity to model changes:** | | | | |
| Asthma (random effect for GP practice)† | 16,540 (1,670,173) | 9.90 | 0.89 | 0.77 to 1.03 |
| **Sensitivity to outcome definition:** | | | | |
| Asthma (age ≥3 years)† | 12,125 (1,687,601) | 7.18 | 0.86 | 0.70 to 1.05 |
| Asthma (QOF Read codes)[#] | 12,512 (1,677,394) | 7.46 | 0.94 | 0.79 to 1.11 |
| Moderate/severe asthma[†,£] | 13,895 (1,676,660) | 8.29 | 0.84 | 0.72 to 0.99 |
| Moderate/severe asthma[#,£] | 10,987 (1,681,617) | 6.53 | 0.89 | 0.74 to 1.07 |

* After adjustment for: child's age, year of delivery, mode of birth.

† Asthma defined based on Read codes used in the main analysis.

‡ Sensitivity to definition of timing of the prophylactic antibiotic policy change comparing years with >50% uptake of pre-incision antibiotics (2013-2018) vs years with very low uptake of pre-incision antibiotics (2006-2010).

§ Sensitivity analysis evaluating the impact of data recording quality.

\# Defined using a conservative list of Read codes for asthma diagnosis adapted from the Quality and Outcomes Framework (QOF).

£ Defined as having a Read code for asthma AND prescription of corticosteroid inhaler AND (prescription for leikotriene receptor antagonists LTRA OR systemic steroid).

Table S3. Number of children with eczema recorded in primary care (THIN-CPRD) database, overall rate per 1,000 person years and the relative risk of pre-incision compared to post-cord clamping prophylactic antibiotics (sensitivity analyses)

| Outcome | Total N (follow-up time in person years) | Overall rate per 1,000 person years | Pre-incision vs post-cord clamping antibiotics* | |
|---|---|---|---|---|
| | | | IRR | 95% CI |
| **Sensitivity to population changes:** | | | | |
| Eczema (2006-2010 vs 2013-2018)[†,‡] | 81,704 (1,149,270) | 71.10 | 0.99 | 0.96 to 1.02 |
| Eczema (secondary care linked primary care dataset)[†, §] | 61,328 (793,746) | 77.26 | 0.96 | 0.90 to 1.02 |
| **Sensitivity to model changes:** | | | | |
| Eczema (random effect for GP practice)[†] | 102,888 (1,430,708) | 71.91 | 0.99 | 0.95 to 1.04 |
| **Sensitivity to outcome definition:** | | | | |
| Eczema (age ≥1 years)[†,¶] | 46,663 (1,597,254) | 29.21 | 1.02 | 0.94 to 1.10 |
| Eczema (based on Read codes only)[#] | 121,938 (1,367,826) | 89.15 | 0.98 | 0.94 to 1.02 |
| Moderate/severe eczema[†,£] | 44,380 (1,565,679) | 28.35 | 0.96 | 0.90 to 1.04 |

* After adjustment for: child's age, year of delivery, mode of birth.

† Eczema defined based on Read codes and two prescriptions on separate dates used in primary analysis.

‡ Sensitivity to definition of timing of the prophylactic antibiotic policy change comparing years with >50% uptake of pre-incision antibiotics (2013-2018) vs years with very low uptake of pre-incision antibiotics (2006-2010).

§ Sensitivity analysis evaluating the impact of data recording quality.

¶ Definition further restricted to children aged ≥1 years AND at least two prescriptions within 90 days before or 365 days after the first eczema Read code record.

# Defined using eczema Read codes only.

£ Defined as having a Read code for eczema AND two prescriptions on separate dates, with at least one prescription for moderate/severe eczema (moderate topical steroid/potent topical steroid/very potent steroid/topical calcineurin/systemic therapy/phototherapy) as per NICE guideline for eczema 2007.

## Table S4. Sensitivity analyses for secondary outcomes recorded in primary care (THIN-CPRD) database with unexpected findings in the main analysis

| Outcome | Total N (follow-up time in person years) | Overall rate per 1,000 person years | Pre-incision vs post-cord clamping antibiotics* | |
|---|---|---|---|---|
| | | | IRR | 95% CI |
| **Antibiotic prescribing:** | | | | |
| 2006-2010 vs 2013-2018† | 268,872 (629,840) | 426.89 | 1.01 | 1.00 to 1.03 |
| Secondary care linked dataset only‡ | 192,224 (443,140) | 433.78 | 1.02 | 0.98 to 1.06 |
| Random effect for GP practice | 336,893 (783,562) | 429.95 | 1.03 | 1.00 to 1.05 |
| **Attention deficit hyperactivity disorder (ADHD)** §**:** | | | | |
| 2006-2010 vs 2013-2018† | 344 (1,364,177) | 0.25 | 1.24 | 0.66 to 2.34 |
| Secondary care linked dataset only‡ | 237 (950,702) | 0.25 | 2.42 | 0.61 to 9.58 |
| **Consultations recorded in primary care (in the first 12 months)** §**:** | | | | |
| 2006-2010 vs 2013-2018† | 5,319,832 (414,034) | 12,848.79 | 1.01 | 1.01 to 1.02 |
| Secondary care linked dataset only‡ | 3,850,551 (295,070) | 13,049.62 | 1.02 | 1.01 to 1.03 |

* After adjustment for: child's age, year of delivery, mode of birth.

† Sensitivity to definition of timing of the prophylactic antibiotic policy change comparing years with >50% uptake of pre-incision antibiotics (2013-2018) vs years with very low uptake of pre-incision antibiotics (2006-2010).

‡ Sensitivity analysis evaluating the impact of data recording quality.

§ Random effect model for GP practice for this outcome did not converge and so no results were obtained.

## Table S5. Impact of pre-incision vs post-cord clamping antibiotics on primary outcomes by emergency and elective caesarean section in primary care (THIN-CPRD) database

| Outcome | CS type | Total N (follow-up time in person years) | Overall rate per 1,000 person years | Pre-incision vs post-cord clamping antibiotics* (based on national policy uptake %) | | | |
|---|---|---|---|---|---|---|---|
| | | | | IRR | 95% CI | p-value | p-value interaction |
| Asthma | Emergency | 2,256 (210,139) | 10.74 | 1.05 | 0.86 to 1.27 | 0.65 | 0.15 |
| | Elective | 1,786 (179,873) | 9.93 | 0.86 | 0.69 to 1.07 | 0.16 | |
| Eczema | Emergency | 14,564 (176,401) | 82.56 | 1.03 | 0.97 to 1.09 | 0.38 | 0.28 |
| | Elective | 10,428 (155,825) | 66.92 | 0.98 | 0.92 to 1.05 | 0.58 | |

* After adjustment for: child's age, year of delivery, mode of birth (vaginal delivery). The model incorporates a probability that each mother received pre-incision antibiotics based on national policy uptake rates in the year of delivery.

## Results of sensitivity analyses in HES

### Table S6. Number of children with hospital admissions for asthma recorded in HES database, overall rate per 1,000 person years and the relative risk of pre-incision vs post-cord clamping prophylactic antibiotics (sensitivity analyses)

| Outcome | Total N (follow-up time in person years) | Overall rate per 1,000 person years | Pre-incision vs post-cord clamping antibiotics* | |
|---|---|---|---|---|
| | | | IRR | 95% CI |
| Asthma (based on national policy uptake % in England)† | 56,748 (33,094,684) | 1.71 | 1.04 | 0.97 to 1.11 |
| Asthma (random effect for hospital) | 30,274 (18,137,690) | 1.67 | 1.03 | 0.97 to 1.10 |
| Asthma (discordant sibling analysis) | 3,756 (2,183,199) | 1.72 | 0.97 | 0.83 to 1.13 |

* After adjustment for: year of delivery, mode of birth.

† Analysis of the full HES dataset using the estimated probability of introduction of pre-incision antibiotics according to calendar year.

### Table S7. Number of children with hospital admissions for eczema recorded in HES database, overall rate per 1,000 person years and the relative risk of pre-incision vs post-cord clamping prophylactic antibiotics (sensitivity analyses)

| Outcome | Total N (follow-up time in person years) | Overall rate per 1,000 person years | Pre-incision vs post-cord clamping antibiotics* | |
|---|---|---|---|---|
| | | | IRR | 95% CI |
| Eczema (based on national policy uptake % in England) † | 2,004 (33,094,684) | 0.06 | 0.78 | 0.58 to 1.06 |
| Eczema (random effect for hospital) | 1,154 (18,137,690) | 0.06 | 0.95 | 0.69 to 1.31 |
| Eczema (discordant sibling analysis)‡ | 124 (2,183,199) | 0.06 | - | - |

* After adjustment for: year of delivery, mode of birth.

† Analysis of the full HES dataset using the estimated probability of introduction of pre-incisional antibiotics according to calendar year.

‡ Too few cases (<200) in HES dataset to fit the model.

### Table S8. Sensitivity analyses for secondary outcomes recorded in HES dataset with unexpected findings in the main analysis

| Outcome | Total N (follow-up time in person years) | Overall rate per 1,000 person years | Pre-incision vs post-cord clamping antibiotics* | |
|---|---|---|---|---|
| | | | IRR | 95% CI |
| **Early onset neonatal sepsis†:** | | | | |
| Based on national policy uptake % in England‡ | 6,075 (7,147,884) | 0.85 | 0.68 | 0.58 to 0.81 |
| Random effect for hospital | 3,336 (3,945,351) | 0.85 | 0.82 | 0.57 to 1.17 |
| **Late onset neonatal sepsis†:** | | | | |
| Based on national policy uptake % in England‡ | 12,629 (7,147,884) | 1.77 | 0.76 | 0.68 to 0.85 |
| Random effect for hospital | 7,226 (3,945,351) | 1.83 | 0.86 | 0.77 to 0.97 |
| **Gastroenteritis§:** | | | | |
| Based on national policy uptake % in England‡ | 249,521 (33,094,684) | 7.54 | 1.08 | 1.05 to 1.11 |
| **Any hospital admission§:** | | | | |
| Based on national policy uptake % in England‡ | 2,572,391 (33,094,684) | 77.73 | 1.02 | 1.01 to 1.02 |

* After adjustment for: year of delivery, mode of birth.
† Due to these conditions occurring in the neonatal period, rates were calculated per 1,000 births.
‡ Analysis of the full HES dataset using the estimated probability of introduction of pre-incision antibiotics according to calendar year.
§ Random effect model for hospital for this outcome did not converge and so no results were obtained.

## Results of exploratory subgroup analyses

### Table S9. Impact of pre-incision vs post-cord clamping antibiotics on primary outcomes by emergency and elective caesarean section in HES database

| Outcome | CS type | Total N (follow-up time in person years) | Overall rate per 1,000 person years | Pre-incision vs post-cord clamping antibiotics* (based on the year of policy change in each hospital) | | | |
|---|---|---|---|---|---|---|---|
| | | | | IRR | 95% CI | p-value | p-value interaction |
| Asthma | Emergency | 4,984 (2,675,442) | 1.86 | 1.05 | 0.98 to 1.13 | 0.17 | 0.90 |
| | Elective | 3,104 (1,897,257) | 1.64 | 1.04 | 0.96 to 1.14 | 0.34 | |
| Eczema | Emergency | 175 (2,675,442) | 0.07 | 0.87 | 0.60 to 1.26 | 0.47 | 0.34 |
| | Elective | 106 (1,897,257) | 0.06 | 1.15 | 0.73 to 1.80 | 0.55 | |

* After adjustment for: year of delivery, mode of birth (vaginal delivery). The model is based on the year of prophylactic antibiotic policy change in each hospital.

## Table S10. Impact of timing of prophylactic antibiotics on primary outcomes and necrotising enterocolitis in HES database in units administering the same antibiotic regiment over time

| Outcome | Total N (follow-up time in person years) | Overall rate per 1,000 person years | Pre-incision vs post-cord clamping antibiotics* (based on the year of policy change in each hospital) | | |
|---|---|---|---|---|---|
| | | | IRR | 95% CI | p-value |
| **Asthma:** | | | | | |
| Cefuroxime alone | 5,697 (3,555,311) | 1.60 | 0.88 | 0.77 to 1.01 | 0.08 |
| Cefuroxime and metronidazole | 5,236 (2,951,897) | 1.77 | 1.04 | 0.91 to 1.19 | 0.59 |
| Co-amoxiclav alone | 10,477 (6,013,240) | 1.74 | 1.08 | 0.98 to 1.19 | 0.13 |
| **Eczema:** | | | | | |
| Cefuroxime alone | 253 (3,555,311) | 0.07 | 1.36 | 0.75 to 2.46 | 0.30 |
| Cefuroxime and metronidazole† | 171 (2,951,897) | 0.06 | - | - | - |
| Co-amoxiclav alone | 447 (6,013,240) | 0.07 | 0.55 | 0.32 to 0.97 | 0.04 |
| **Necrotising enterocolitis‡:** | | | | | |
| Cefuroxime alone | 213 (771,945) | 0.28 | 1.38 | 0.83 to 2.28 | 0.22 |
| Cefuroxime and metronidazole | 232 (639,624) | 0.36 | 1.22 | 0.76 to 1.95 | 0.41 |
| Co-amoxiclav alone | 347 (1,310,208) | 0.26 | 1.32 | 0.92 to 1.89 | 0.13 |

* After adjustment for: year of delivery, mode of birth.

† Too few cases (<200) in HES data set to fit the model.

‡ Due to this occurring in the neonatal period, rates were calculated per 1,000 births.

## Covariate graphs (Figures S4-24)

### Maternal and household characteristics

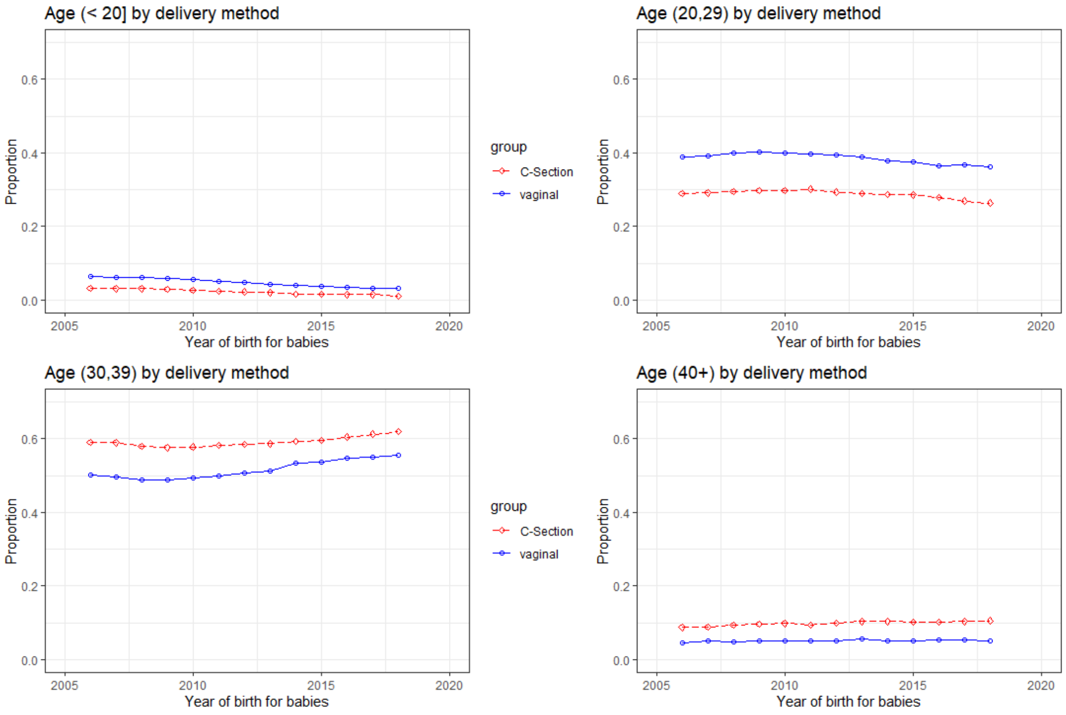


Figure S4 Proportion of delivery mode over time in THIN-CPRD dataset across different maternal age groups at childbirth

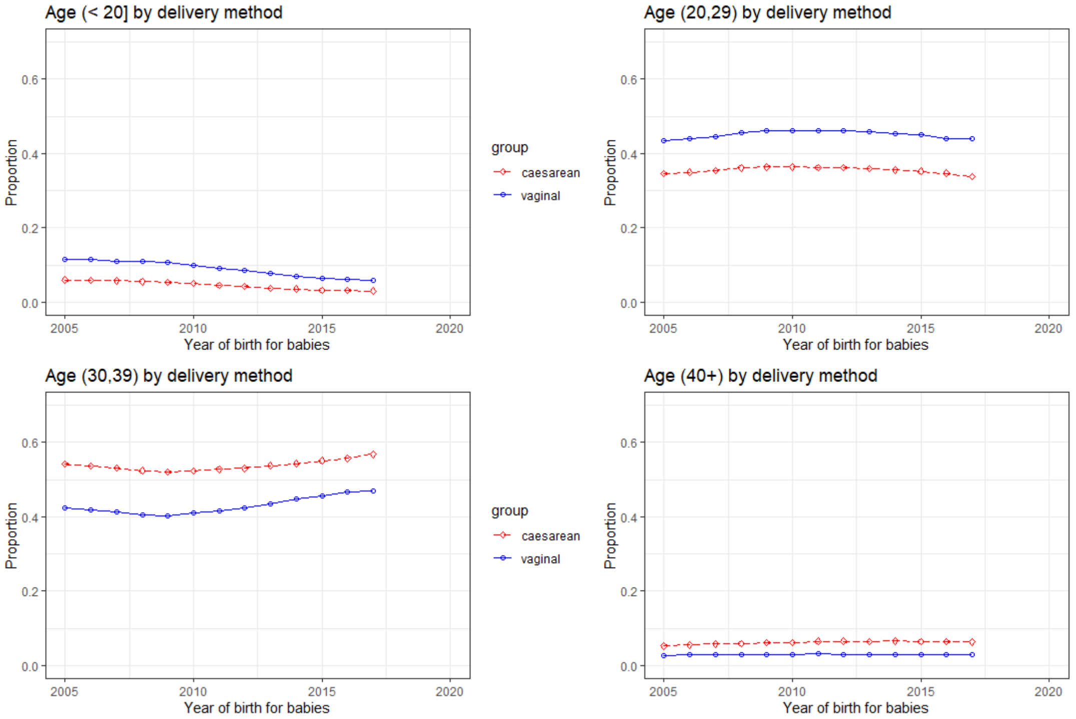


Figure S5 Proportion of delivery mode over time in HES dataset by maternal age groups at childbirth

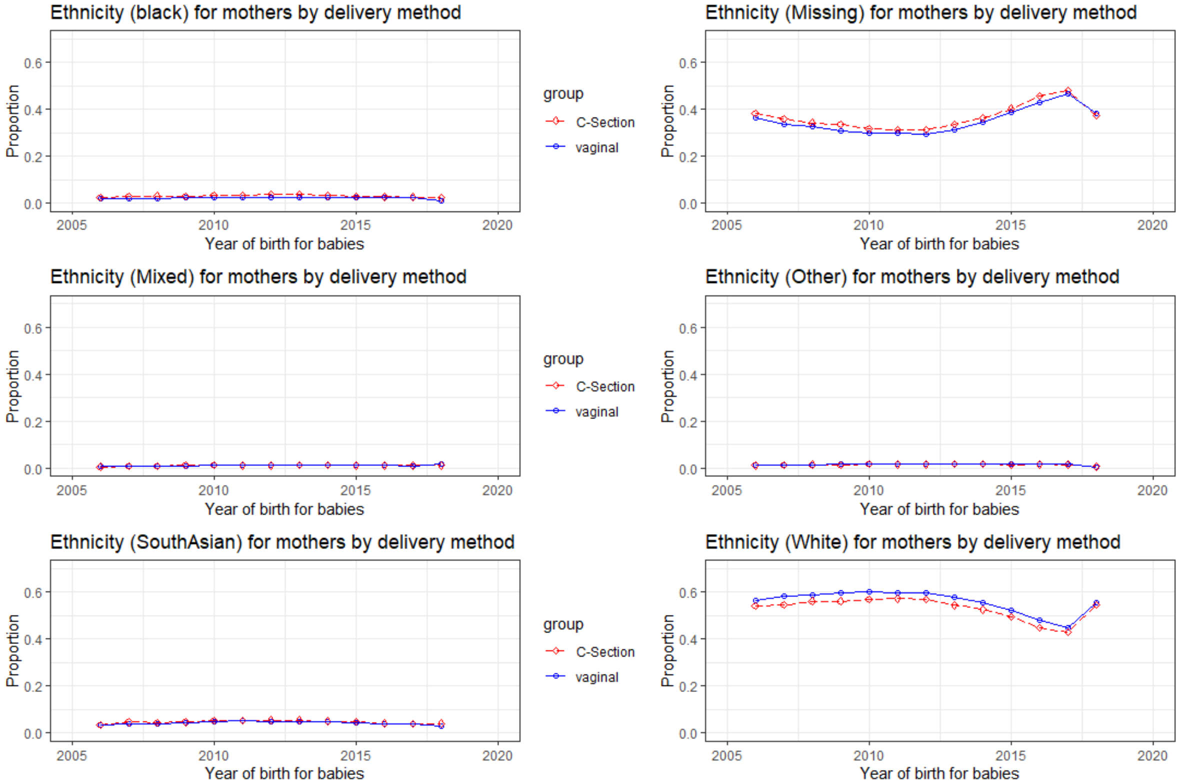


Figure S6 Proportion of delivery mode over time in THIN-CPRD dataset by maternal ethnicity

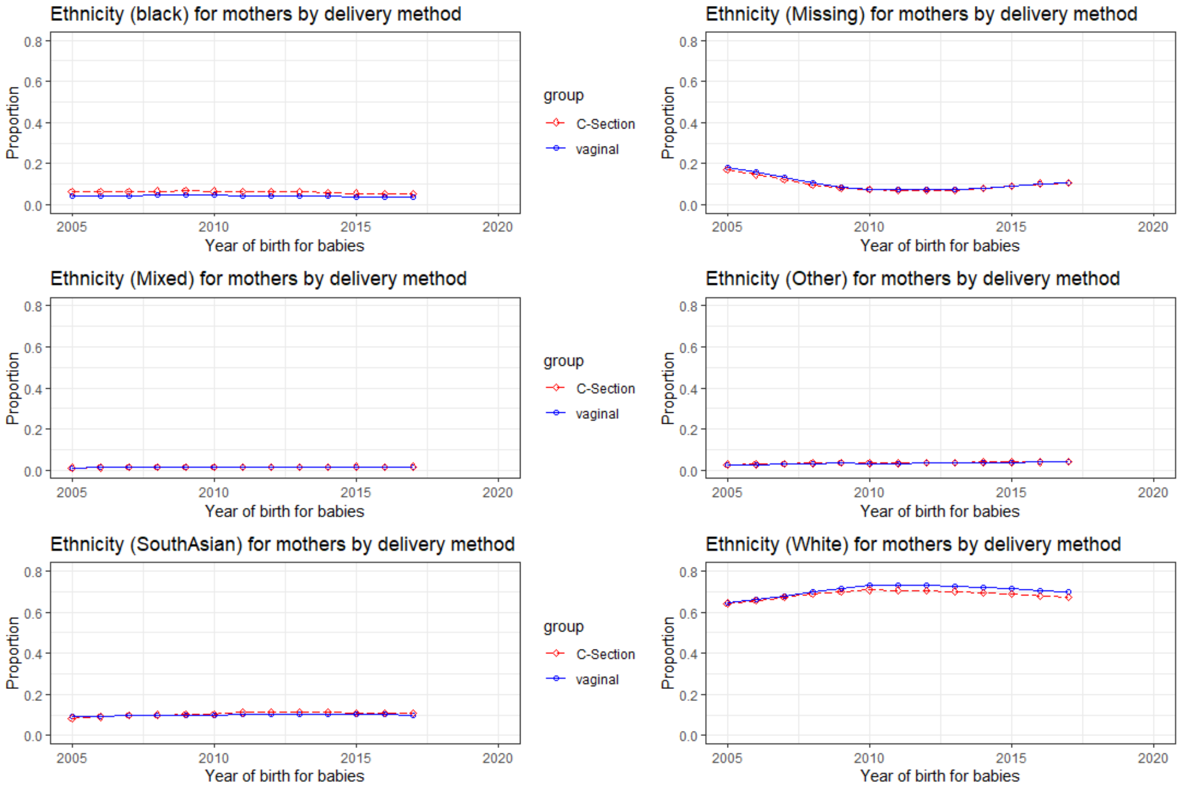


Figure S7 Proportion of delivery mode over time in HES dataset by maternal ethnicity

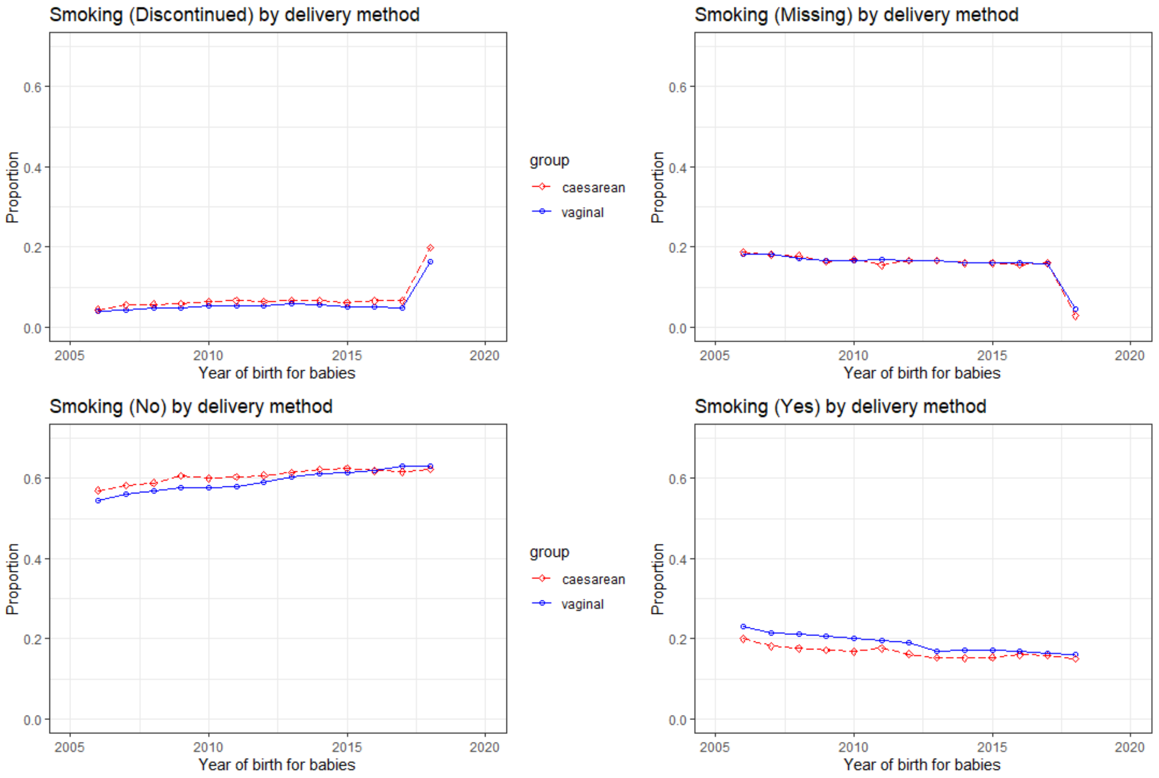


Figure S8 Proportion of delivery mode over time in THIN-CPRD dataset by maternal smoking status before delivery

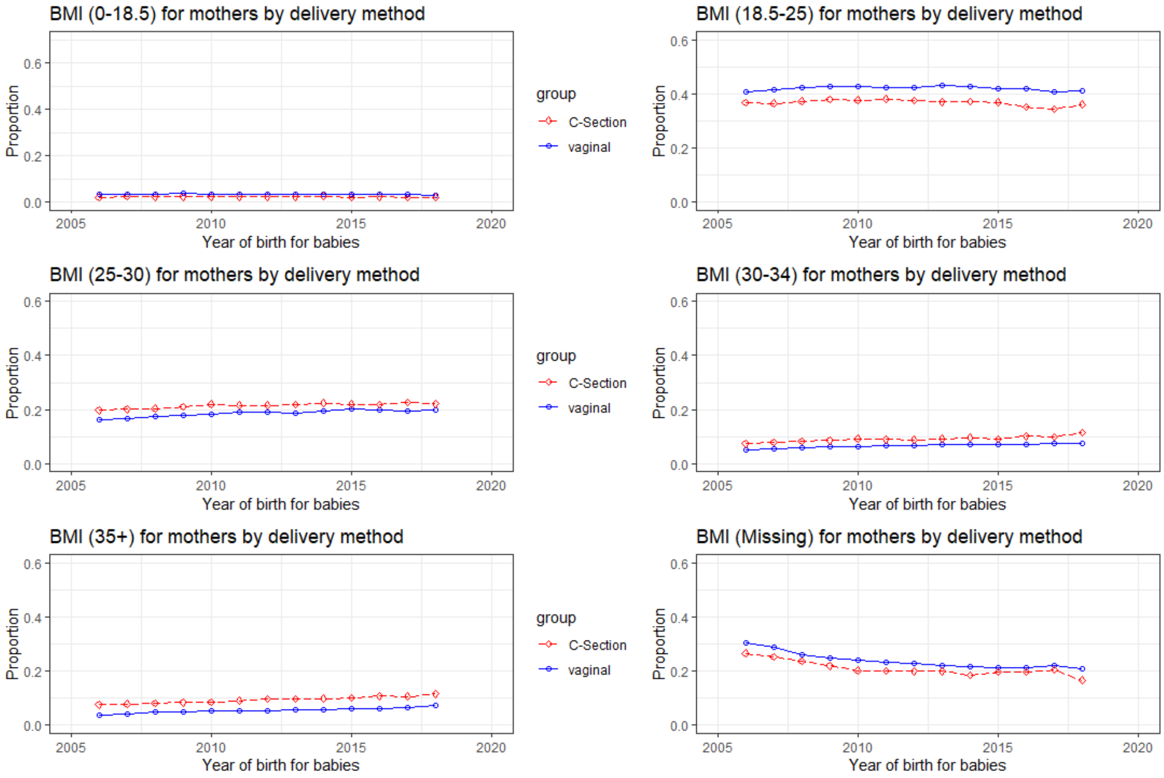


Figure S9 Proportion of delivery mode over time in THIN-CPRD dataset by different maternal BMI groups (latest measurement nine months before delivery)

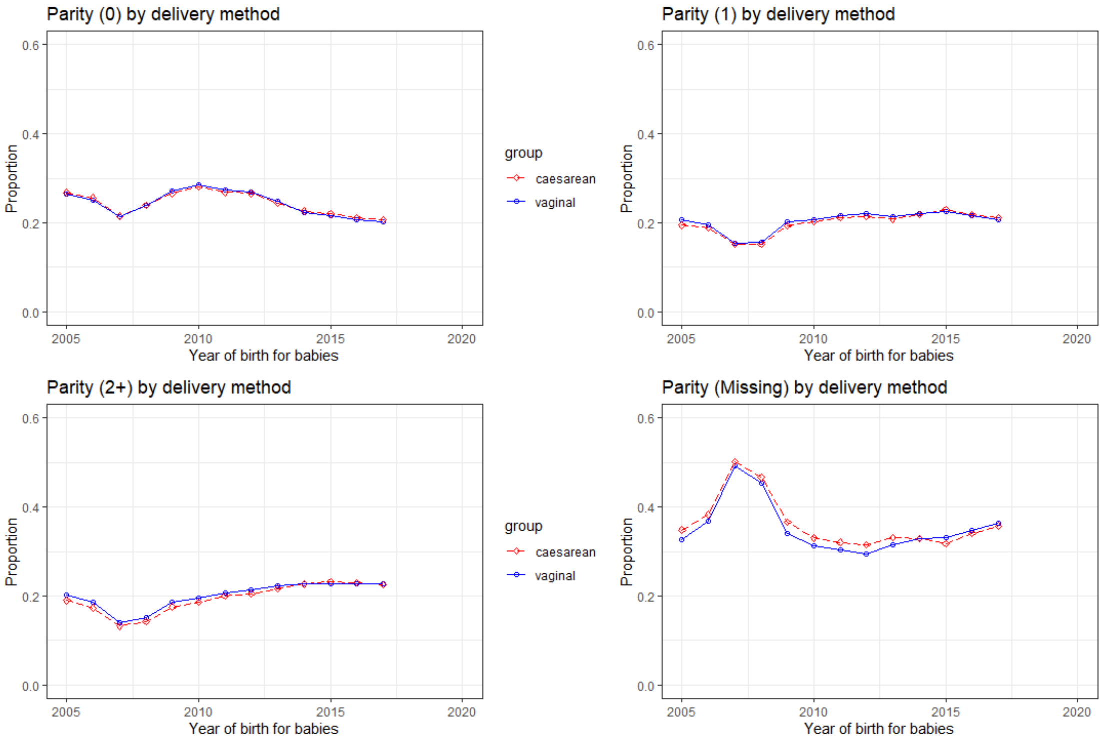


Figure S10 Proportion of delivery mode over time in HES dataset by parity

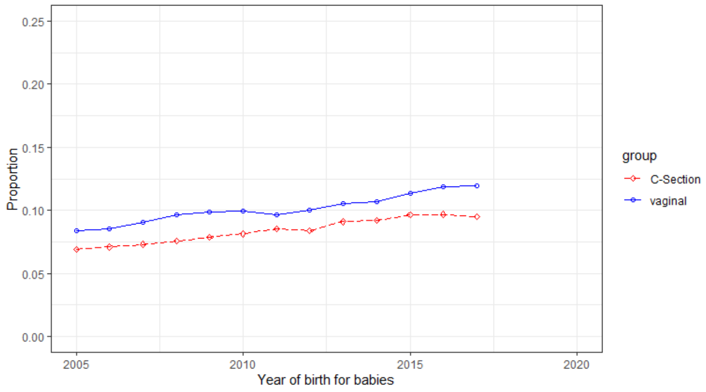


Figure S11 Proportion of delivery mode over time in HES dataset with premature rupture of membranes

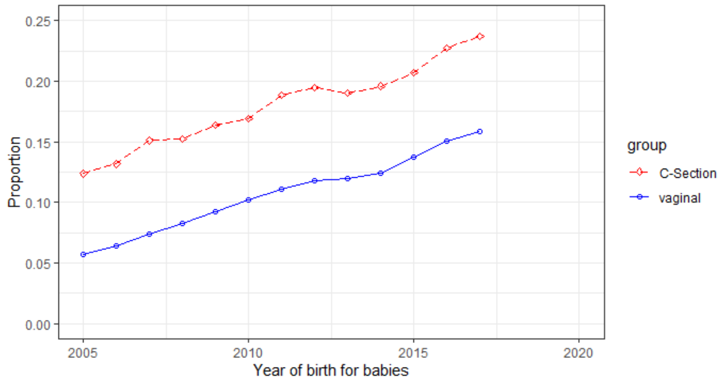


Figure S12 Proportion of delivery mode over time in HES dataset with post-partum haemorrhage

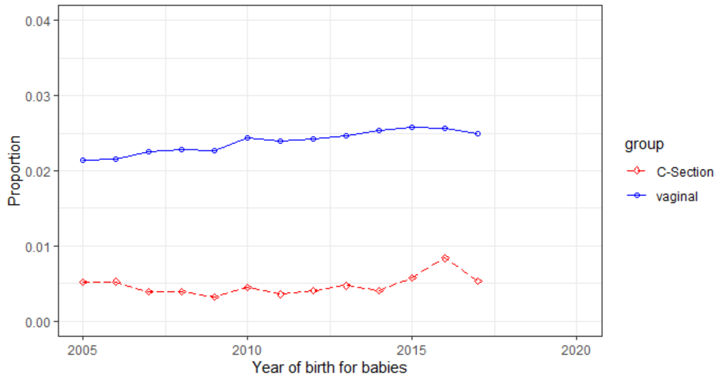


Figure S13 Proportion of delivery mode over time in HES dataset with manual placental removal/ retained products of conception

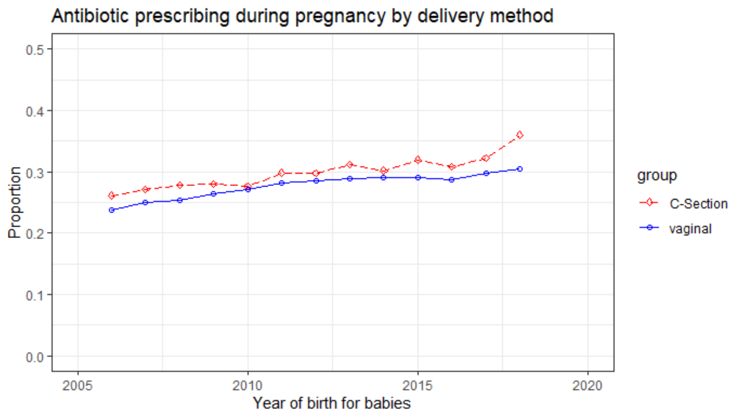


Figure S14 Proportion of women by delivery mode over time in THIN-CPRD dataset who were prescribed antibiotics during pregnancy

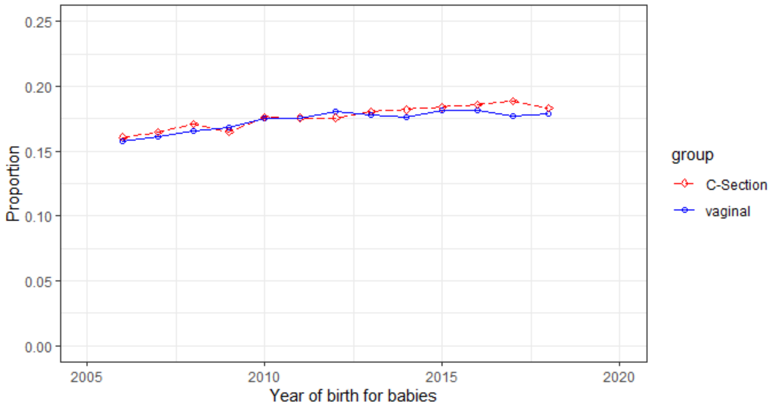


(a)

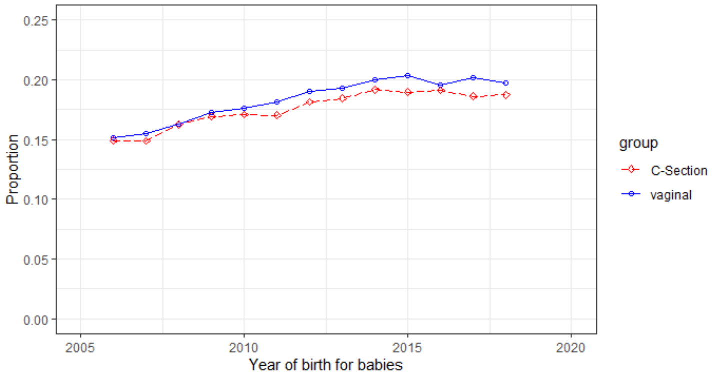


(b)

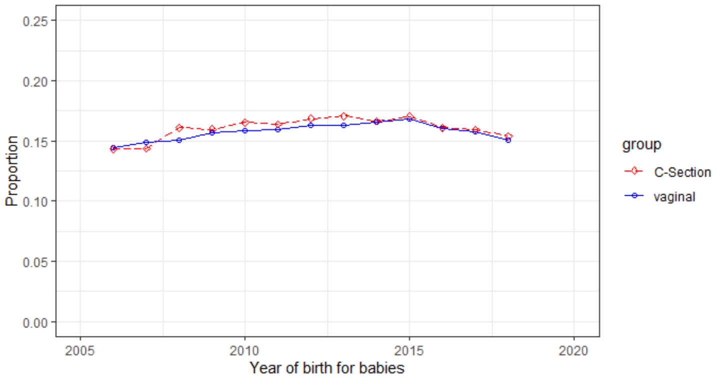


(c)

Figure S15 Proportion of women by delivery mode over time in THIN-CPRD dataset with a record of (a) asthma, (b) eczema and (c) allergic rhinitis and conjunctivitis

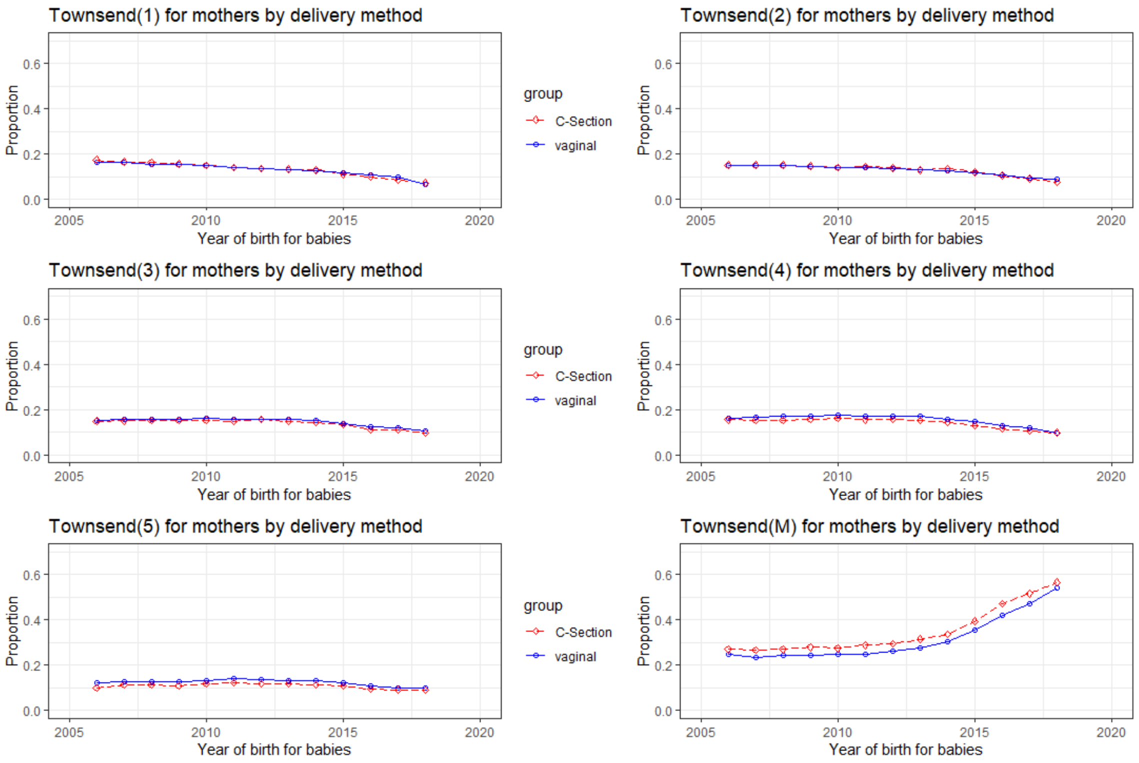


Figure S16 Proportion of delivery mode over time in THIN-CPRD dataset and living in different areas of deprivation (as measured by Townsend score with 1 being most affluent, 5 being most deprived, M missing)

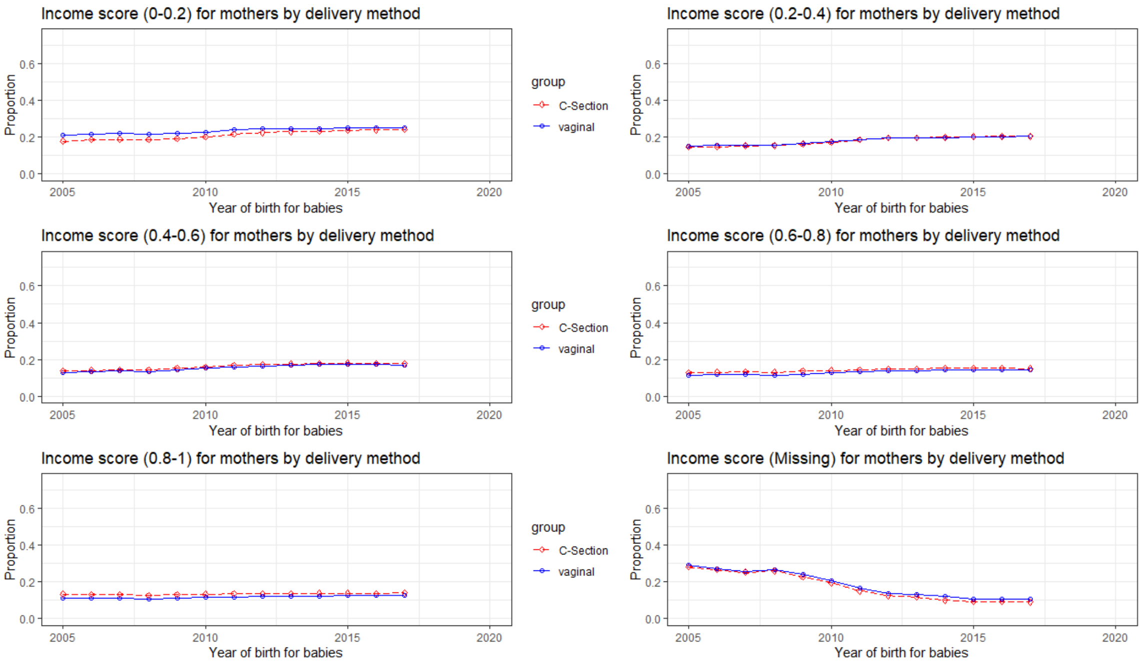


Figure S17 Proportion of delivery mode over time in HES dataset and living in different areas of deprivation (as measured by Index of Multiple Deprivation 2015 score with lowest score being most affluent and the highest score being most deprived)

## Child characteristics

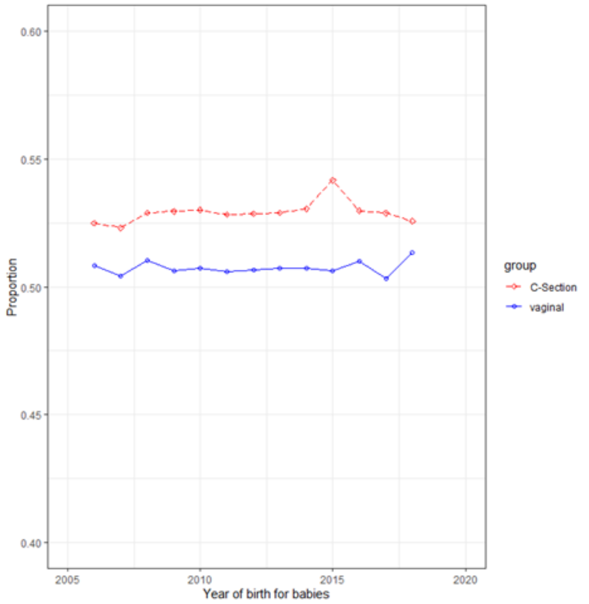


(a)

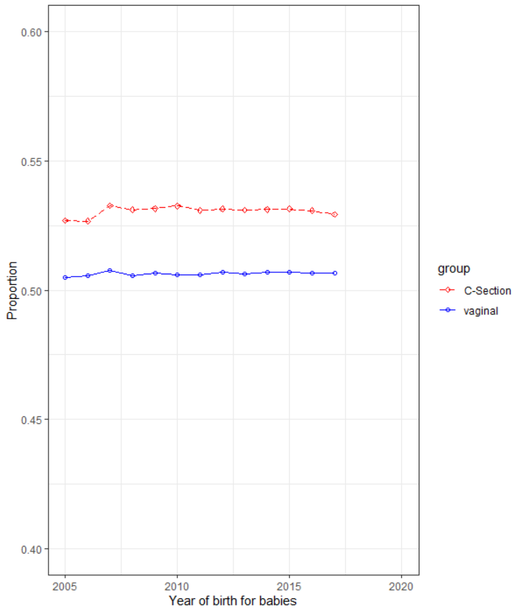


(b)

Figure S18 Proportion of boys in CS and VD births over time in (a) THIN-CPRD dataset, (b) HES dataset

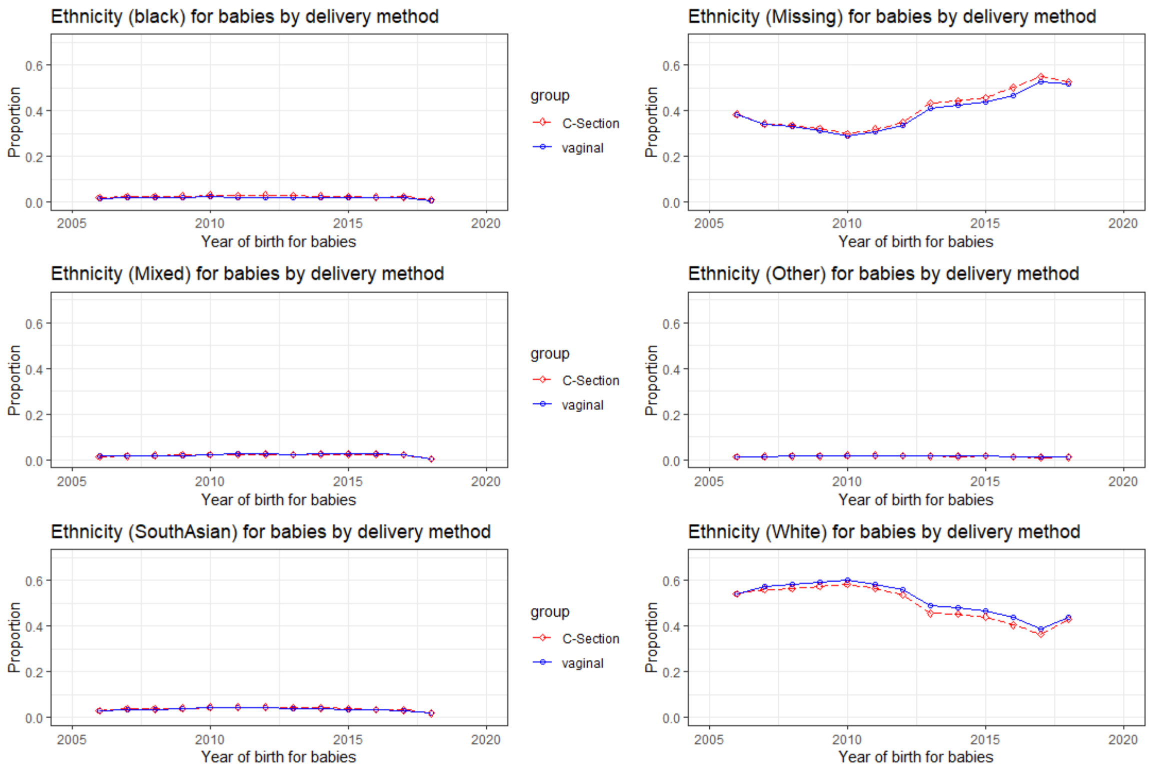


Figure S19 Proportion of delivery mode over time in THIN-CPRD dataset and child’s ethnicity

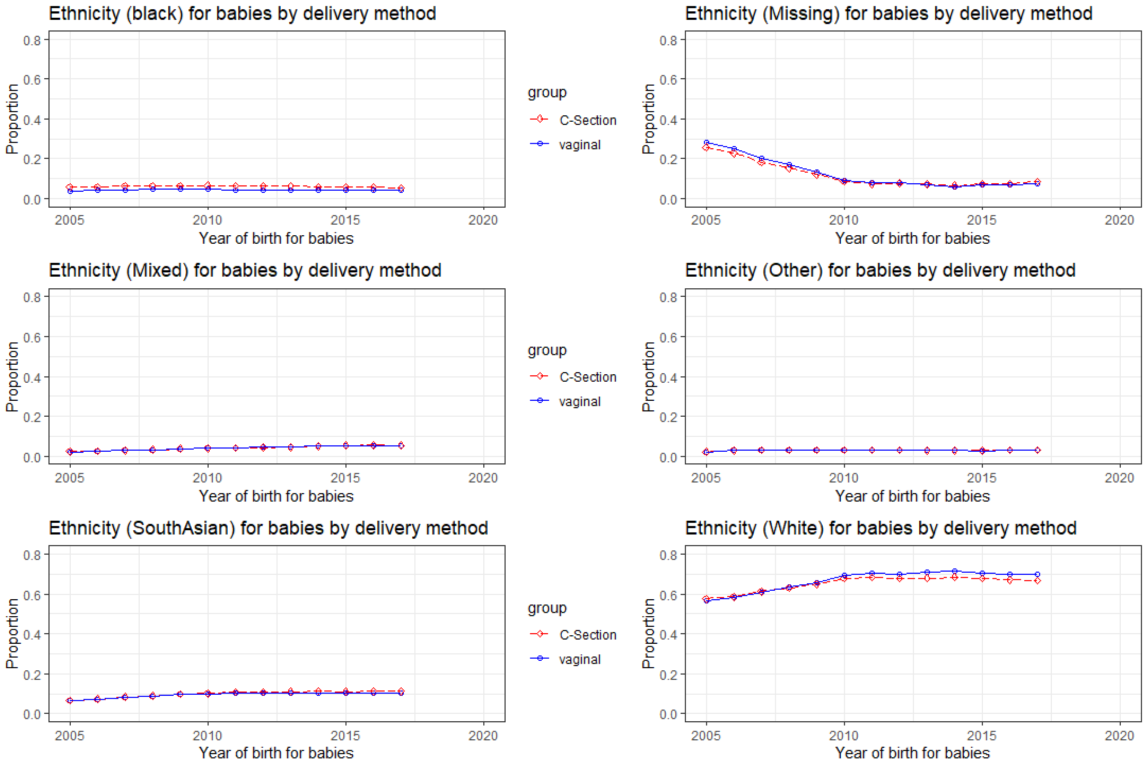


Figure S20 Proportion of delivery mode over time in HES dataset and child's ethnicity

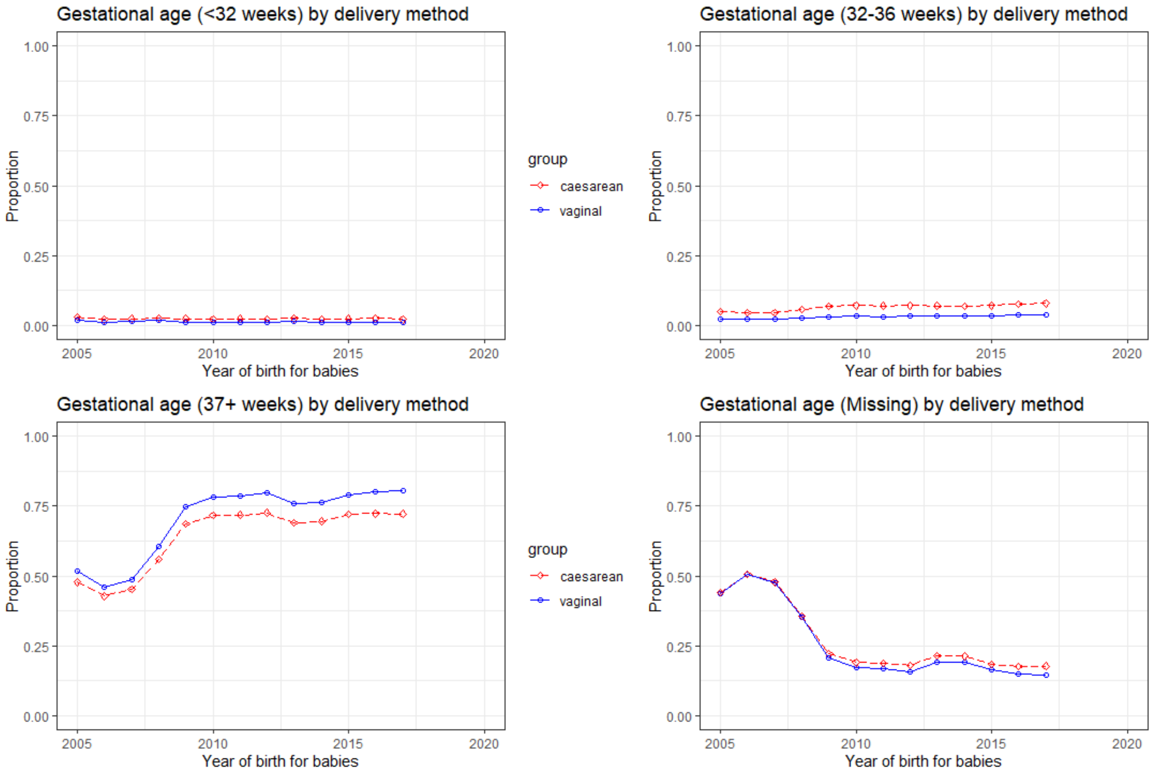


Figure S21 Proportion of delivery mode over time in HES dataset by gestational age

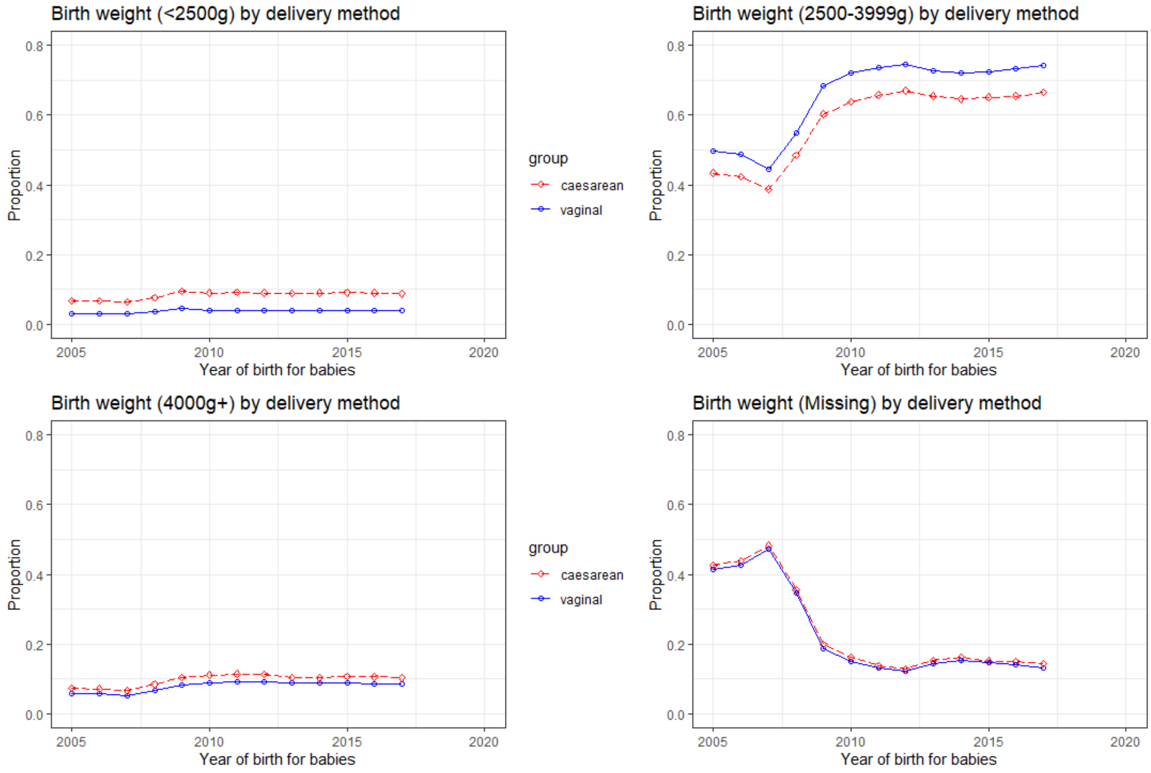


Figure S22 Proportion of delivery mode over time in HES dataset by child's birthweight

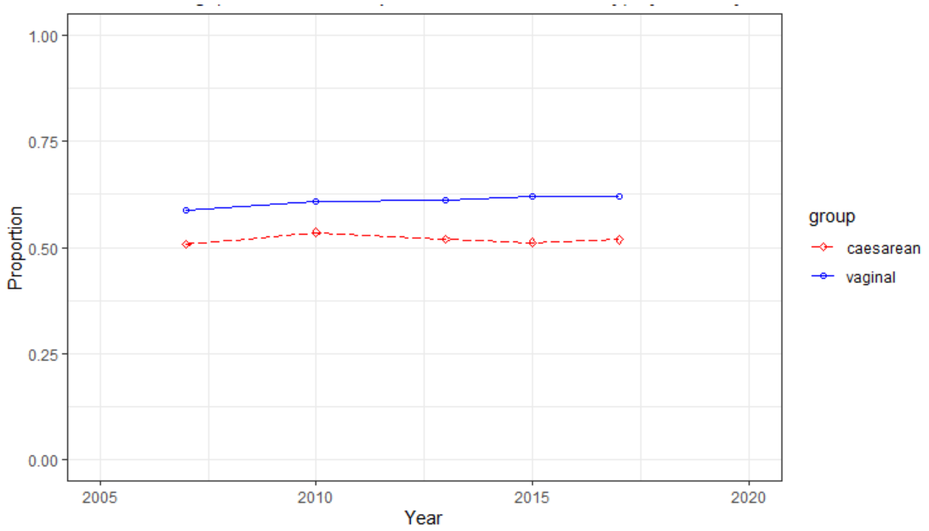


Figure S23 Proportion of women feeding their babies with breast milk only in the few days after birth by delivery mode (data source: Care Quality Commission's Maternity Surveys in 2007, 2010, 2013, 2017)

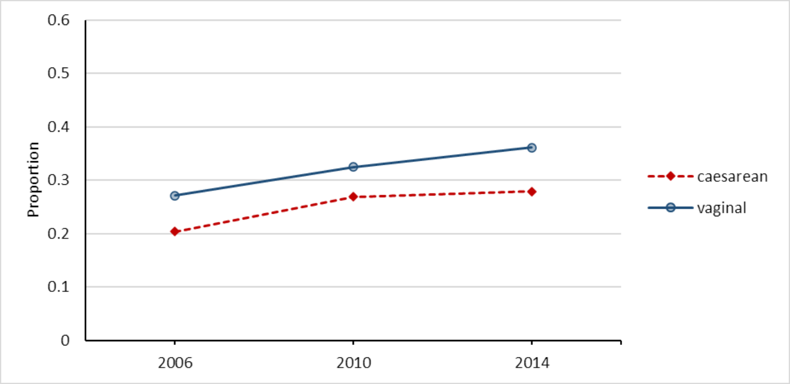


Figure S24 Proportion of women feeding their babies with breast milk only at three months of age by delivery mode (data source: NPEU Maternity Surveys in 2006, 2010, 2014)

## Rates of study outcomes over time by mode of delivery (Tables S11-12 and Figures S25-74)

The graphs below display the rates of different outcomes over the study period. In each graph, the rates are presented separately for CS and VD. For outcomes investigated in the THIN-CPRD dataset, we present graphs for each year of life individually. No confidence intervals have been included in the graphs, but to aid the interpretation of the precision of these rates, we provide some example confidence intervals for the rate of the outcome (per 1,000 person-years) for a series of event rates based on the numbers of events and follow-up time found in THIN-CPRD data (*Table S11*) and HES data (*Table S12*).

Table S11 Example confidence intervals for the outcome rates found in THIN-CPRD data

| Number of events | Follow-up time (person years) | Rate per 1,000 person-years | Confidence interval for rate per 1,000 person years |
|---|---|---|---|
| 2 | 11,868 | 0.17 | 0.02 to 0.61 |
| 71 | 11,850 | 5.99 | 4.68 to 7.56 |
| 1,189 | 11,329 | 104.95 | 99.07 to 111.09 |
| 5,583 | 9,254 | 603.34 | 587.61 to 619.37 |

Table S12 Example confidence intervals for the outcome rates found in HES data

| Number of events | Follow-up time (person years) | Rate per 1,000 person-years | Confidence interval for rate per 1,000 person years |
|---|---|---|---|
| 235 | 764,195 | 0.31 | 0.27 to 0.35 |
| 1,865 | 764,195 | 2.44 | 2.33 to 2.55 |
| 4,864 | 149,522 | 32.53 | 31.62 to 33.46 |
| 53,595 | 764,195 | 70.1 | 69.54 to 70.72 |

## Child outcomes in THIN-CPRD dataset

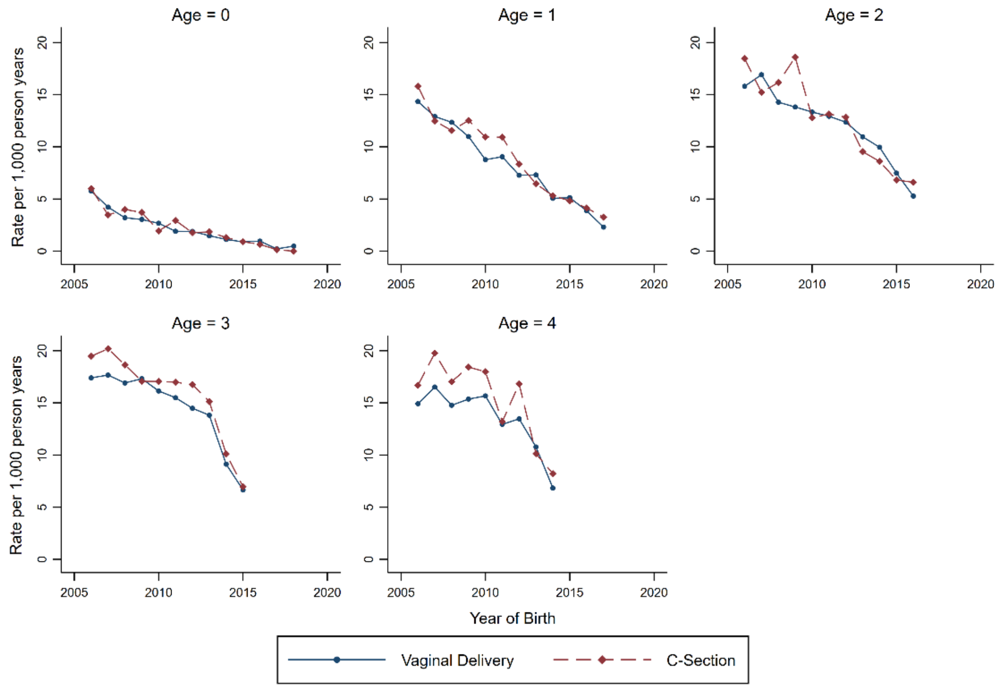


Figure S25 Incidence rate of asthma per 1,000 person years in children born by CS and VD by age and year of birth in THIN-CPRD dataset

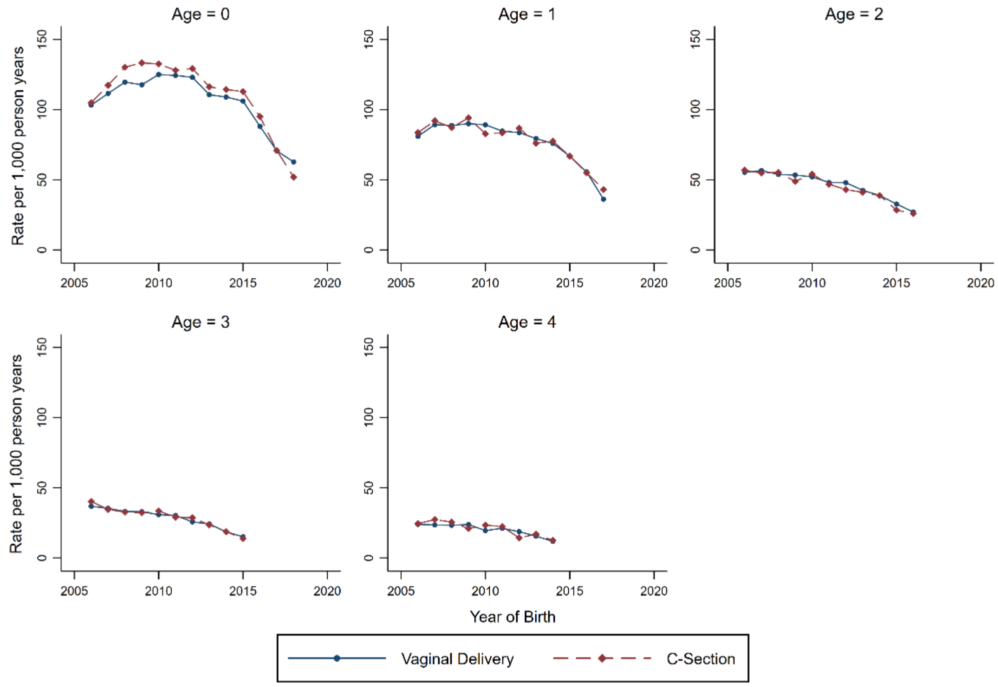


Figure S26 Incidence rate of eczema per 1,000 person years in children born by CS and VD by age and year of birth in THIN-CPRD dataset

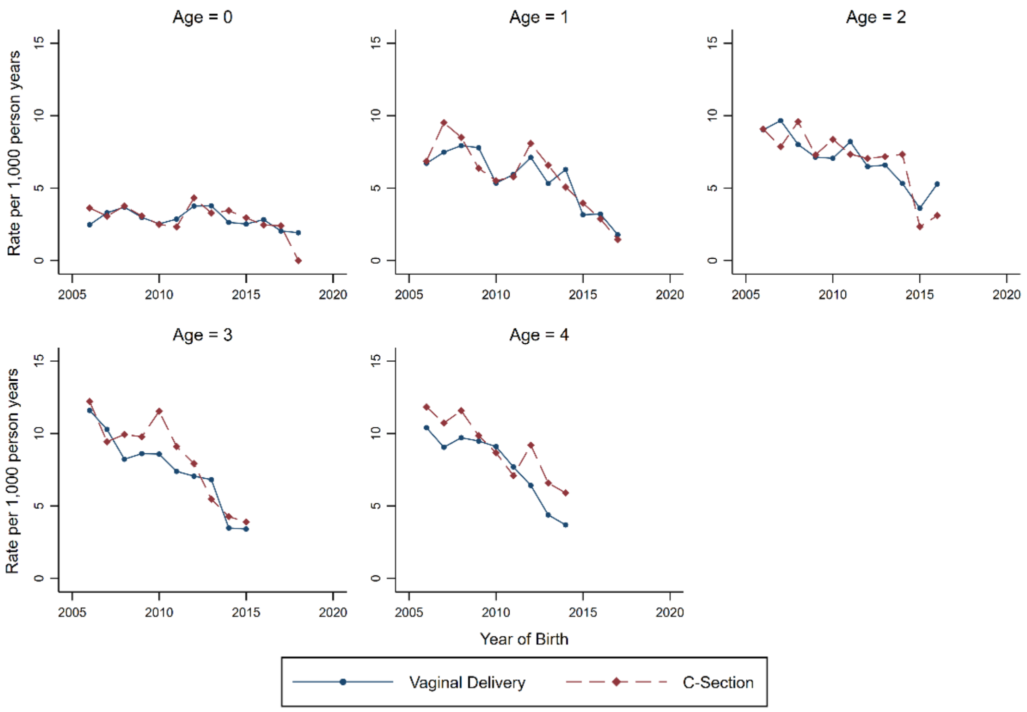


Figure S27 Incidence rate of allergic rhinitis and conjunctivitis per 1,000 person years in children born by CS and VD by age and year of birth in THIN-CPRD dataset

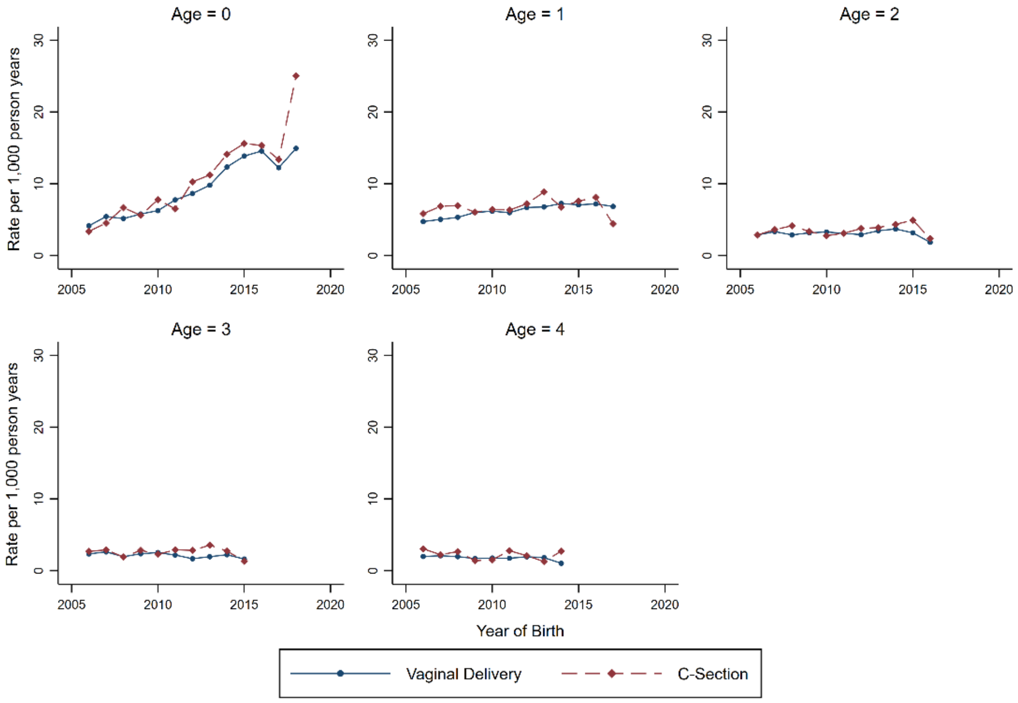


Figure S28 Incidence rate of food allergy and intolerance per 1,000 person years in children born by CS and VD by age and year of birth in THIN-CPRD dataset

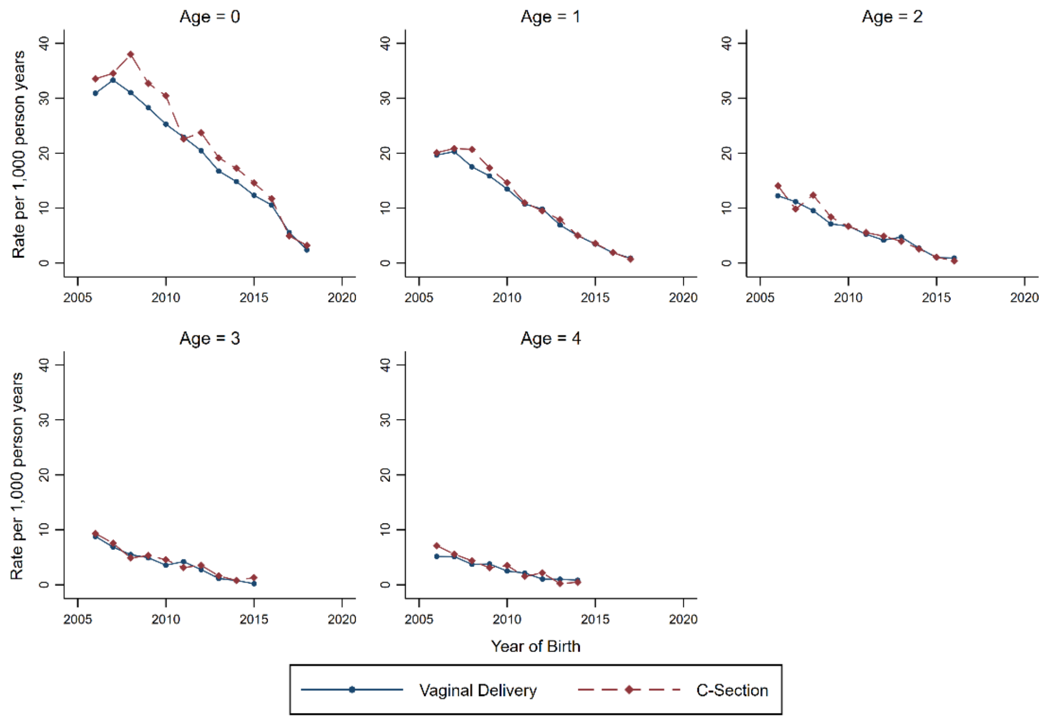


Figure S29 Incidence rate of having two or more allergy related conditions (asthma, eczema, food allergy/intolerance, allergic rhinitis and conjunctivitis) per 1,000 person years in children born by CS and VD by age and year of birth in THIN-CPRD dataset

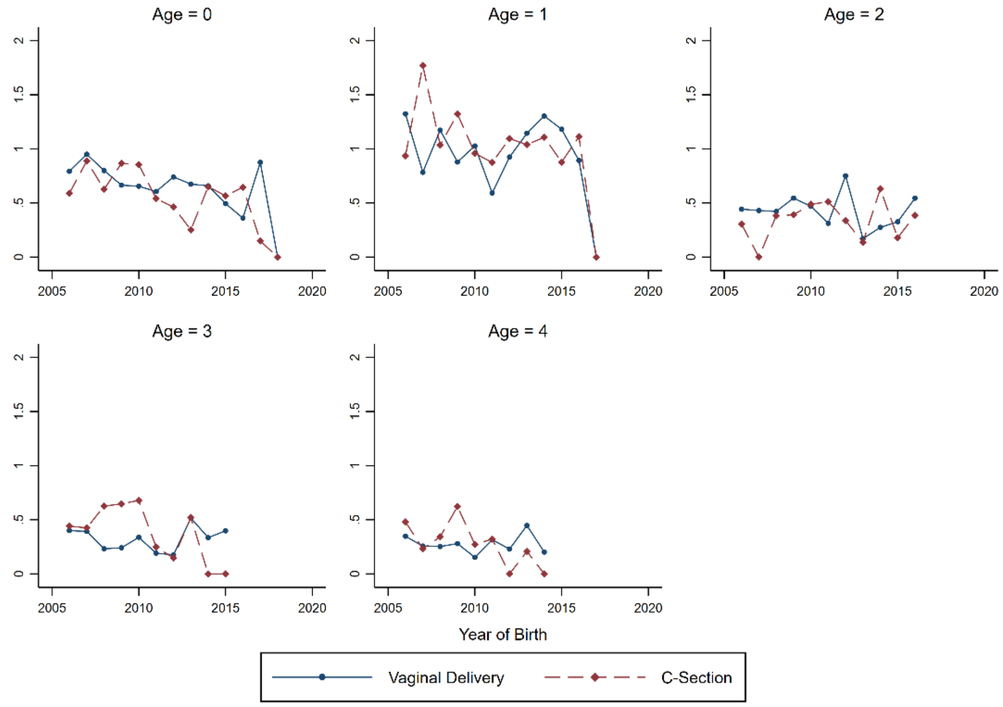


Figure S30 Incidence rate of penicillin allergy per 1,000 person years in children born by CS and VD by age and year of birth in THIN-CPRD dataset

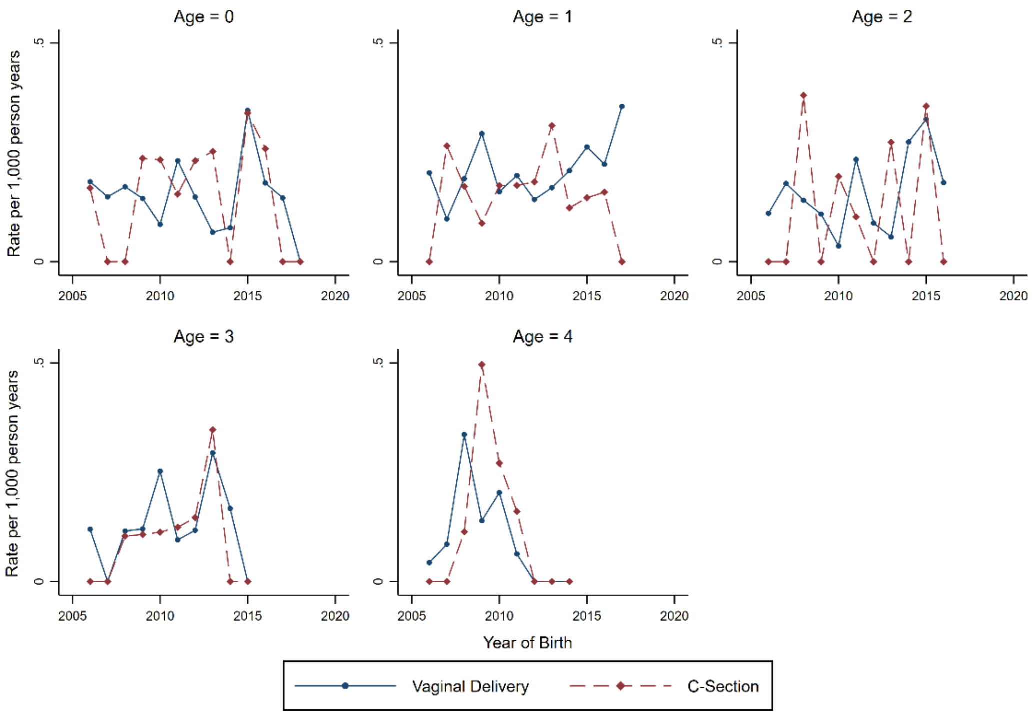


Figure S31 Incidence rate of anaphylaxis per 1,000 person years in children born by CS and VD by age and year of birth in THIN-CPRD dataset

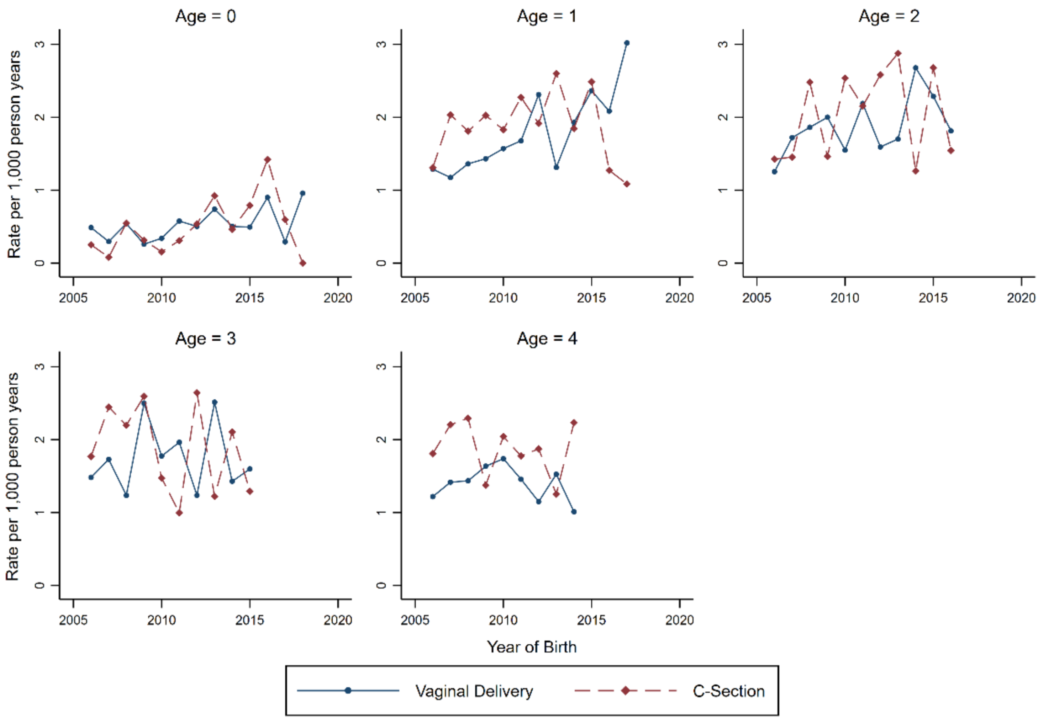


Figure S32 Incidence rate of high risk of anaphylactic reaction (prescribing of automatic injection devices containing adrenaline) per 1,000 person years in children born by CS and VD by age and year of birth in THIN-CPRD dataset

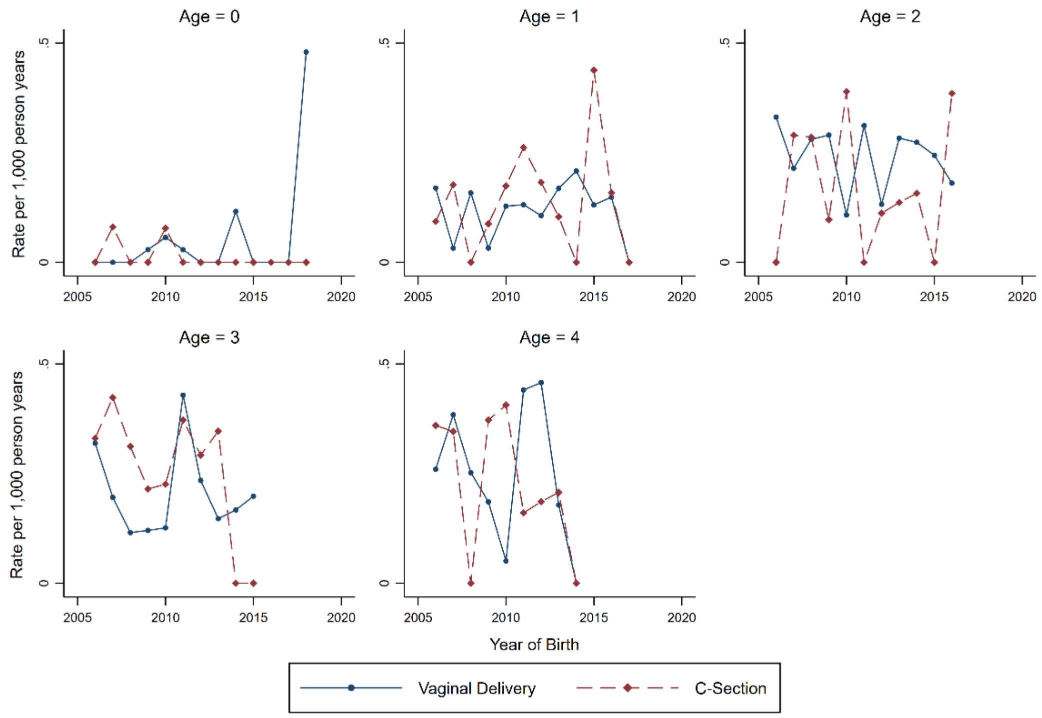


Figure S33 Incidence rate of type 1 diabetes per 1,000 person years in children born by CS and VD by age and year of birth in THIN-CPRD dataset

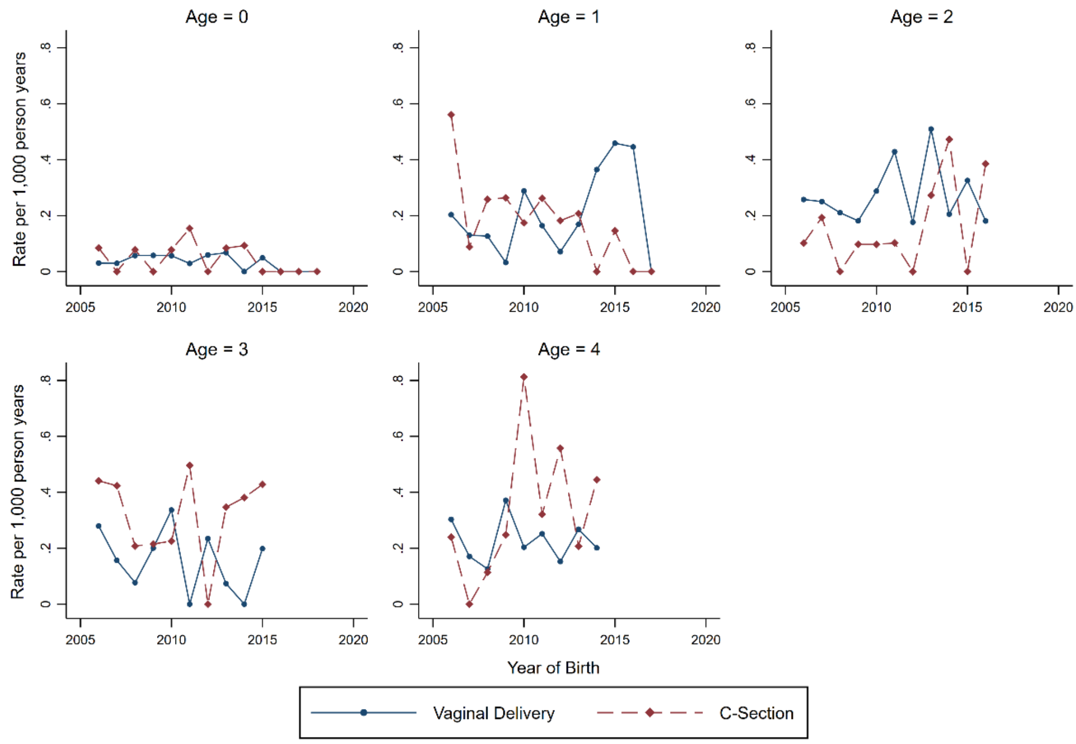


Figure S34 Incidence rate of coeliac disease per 1,000 person years in children born by CS and VD by age and year of birth in THIN-CPRD dataset

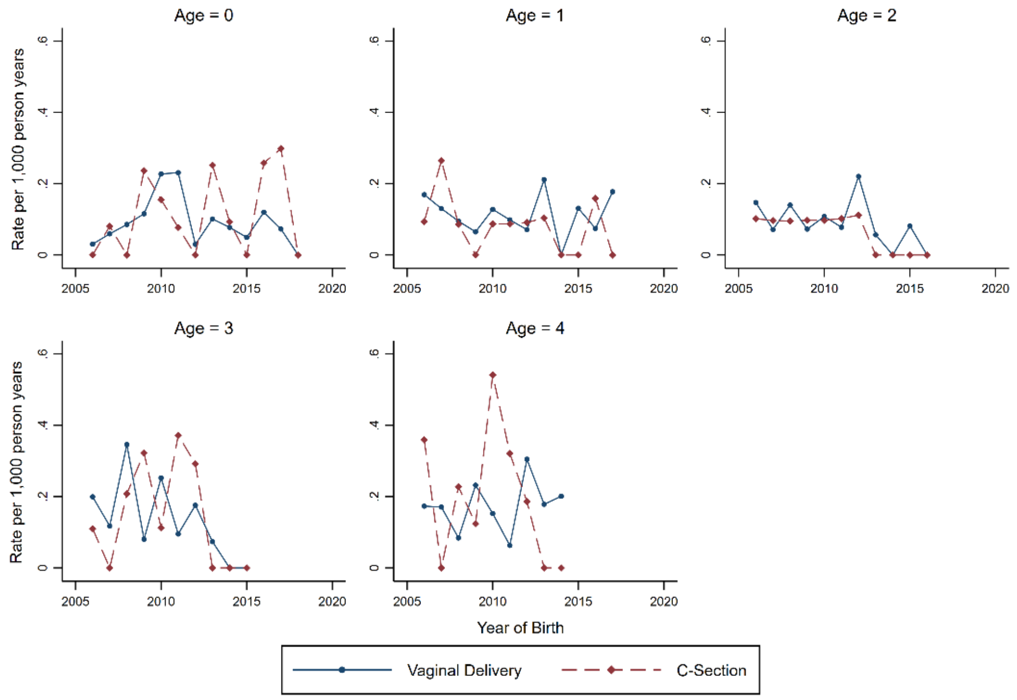


Figure S35 Incidence rate of childhood vitiligo per 1,000 person years in children born by CS and VD by age and year of birth in THIN-CPRD dataset

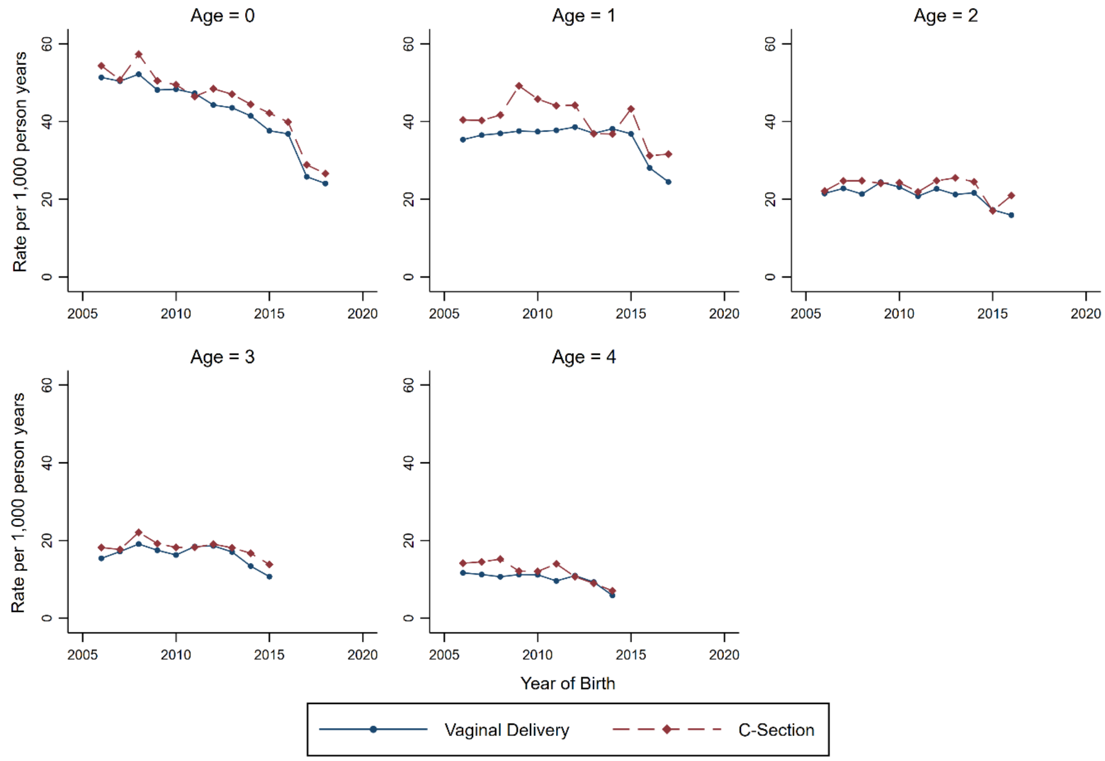


Figure S36 Incidence rate of wheeze per 1,000 person years in children born by CS and VD by age and year of birth in THIN-CPRD dataset

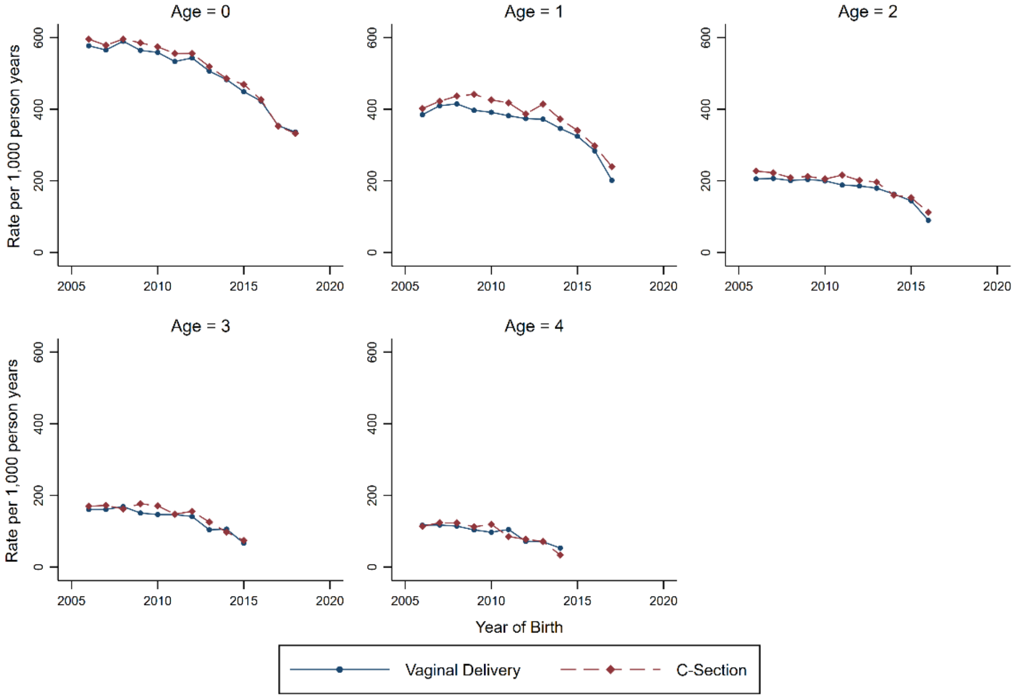


Figure S37 Incidence rate of upper respiratory tract infections per 1,000 person years in children born by CS and VD by age and year of birth in THIN-CPRD dataset

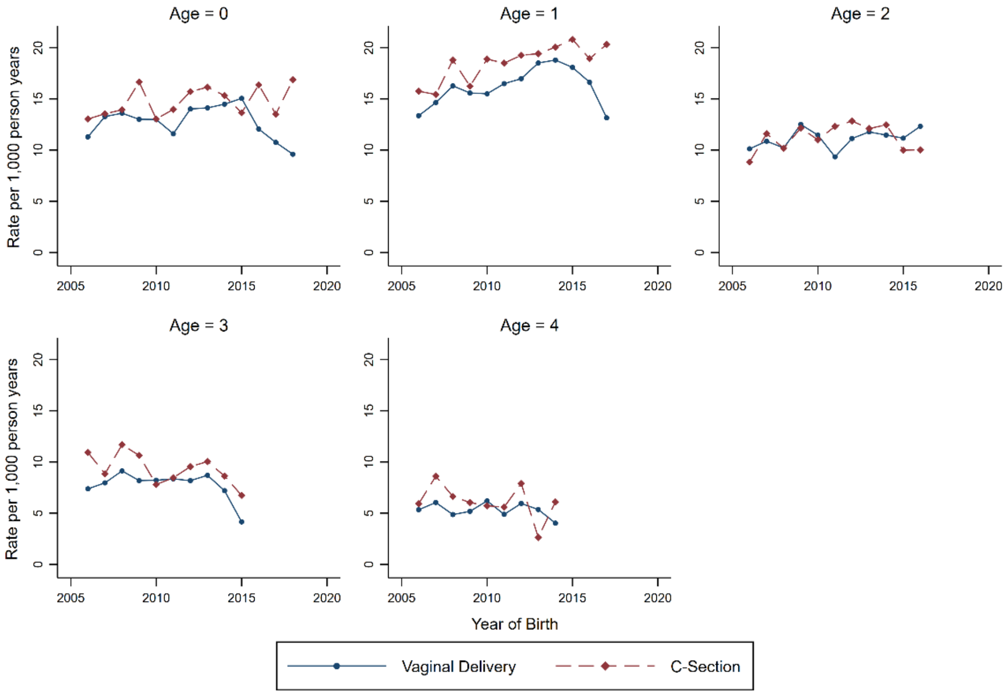


Figure S38 Incidence rate of lower respiratory tract infections per 1,000 person years in children born by CS and VD by age and year of birth in THIN-CPRD dataset

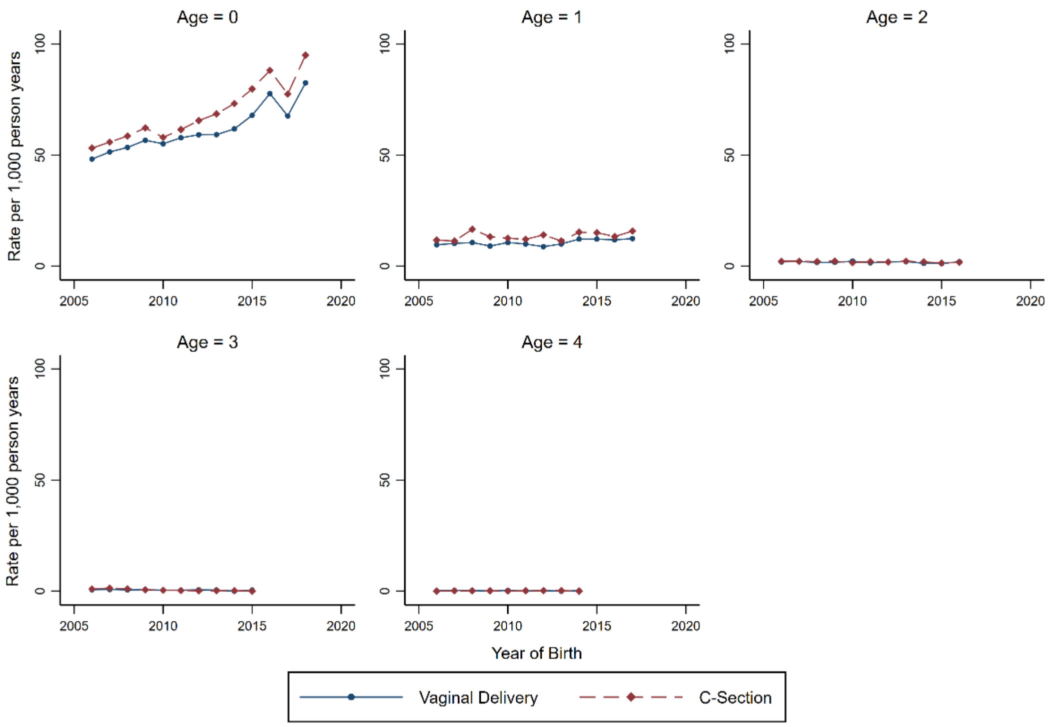


Figure S39 Incidence rate of bronchiolitis per 1,000 person years in children born by CS and VD by age and year of birth in THIN-CPRD dataset

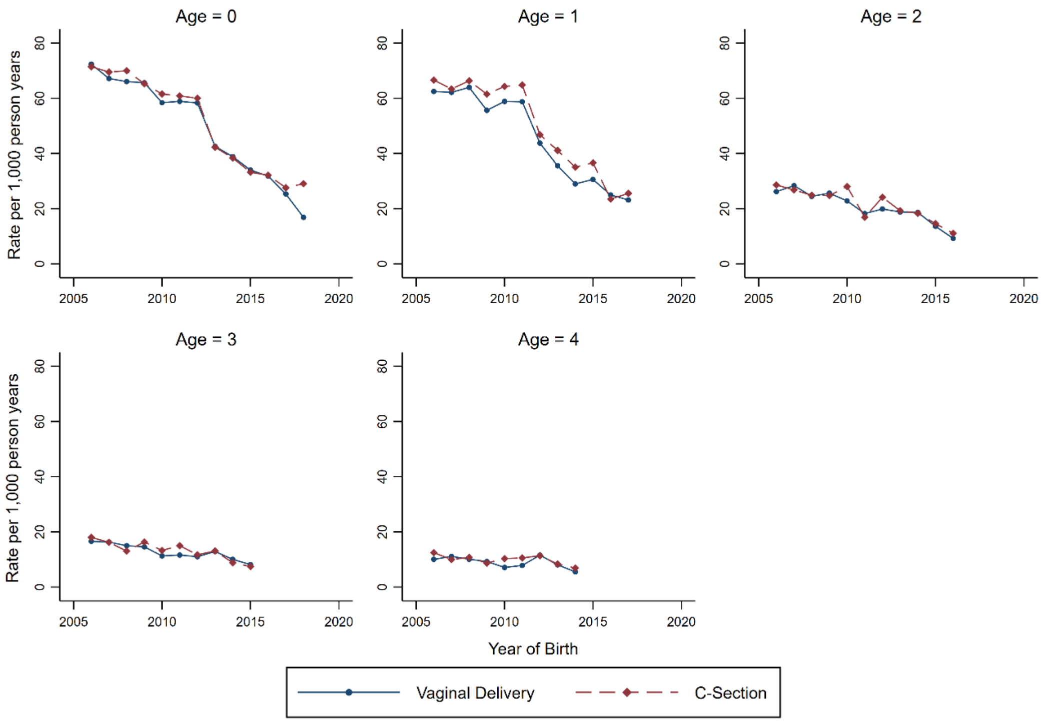


Figure S40 Incidence rate of gastroenteritis per 1,000 person years in children born by CS and VD by age and year of birth in THIN-CPRD dataset

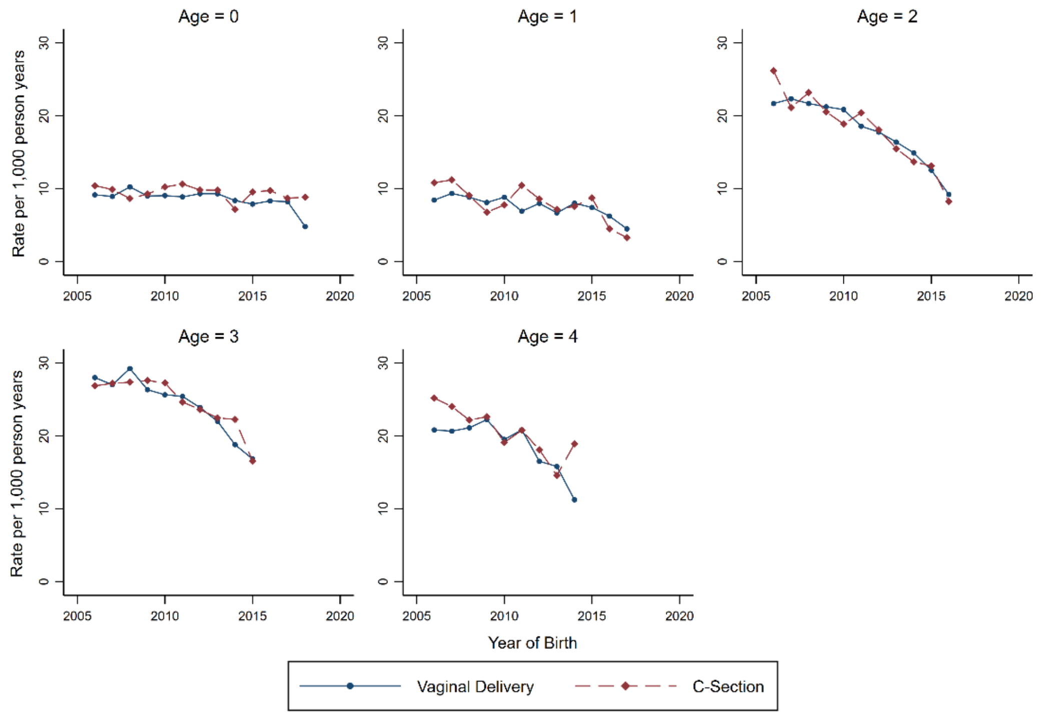


Figure S41 Incidence rate of urinary tract infection per 1,000 person years in children born by CS and VD by age and year of birth in THIN-CPRD dataset

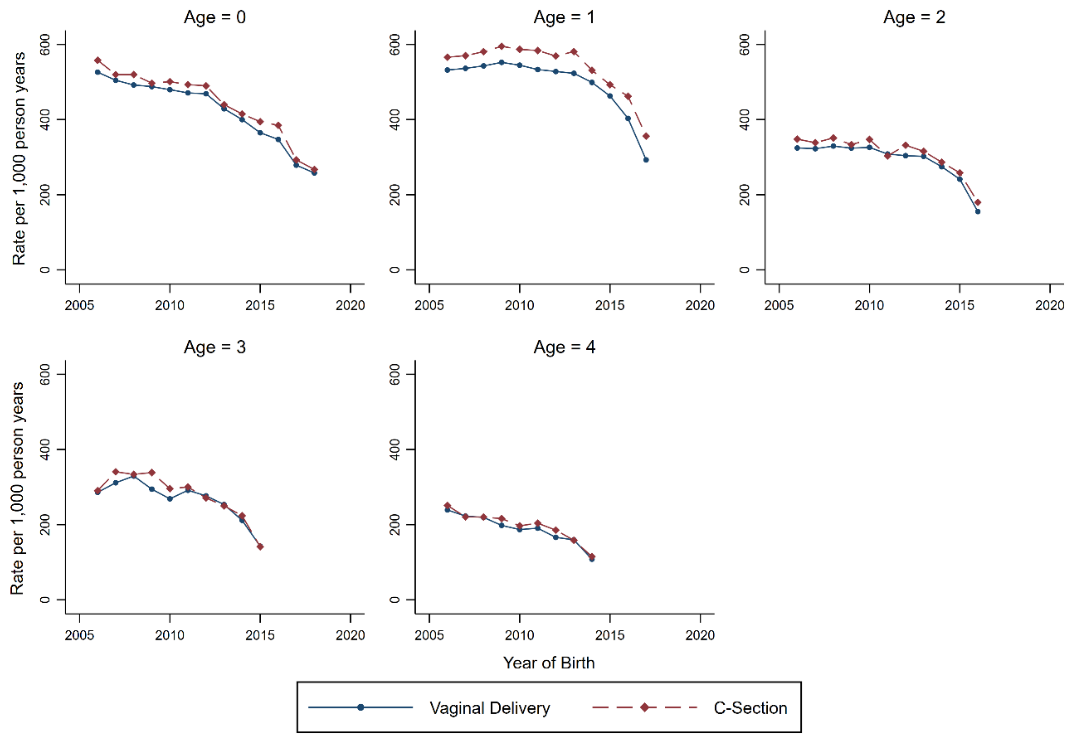


Figure S42 Incidence rate of first-time antibiotic prescription per 1,000 person years in children born by CS and VD by age and year of birth in THIN-CPRD dataset

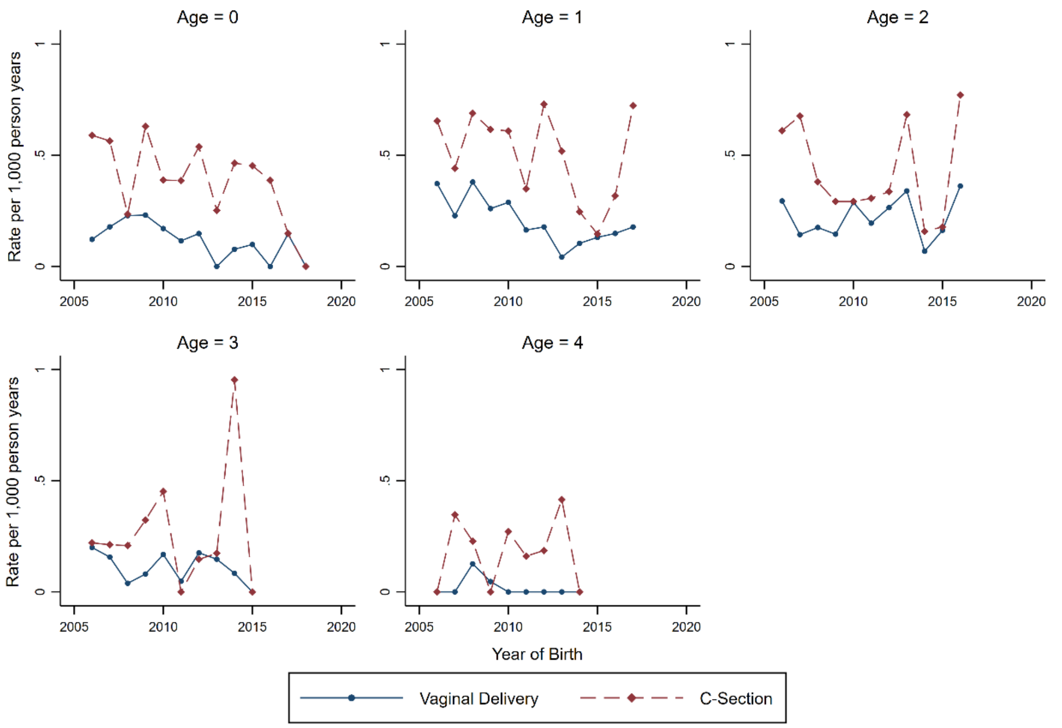


Figure S43 Incidence rate of cerebral palsy per 1,000 person years in children born by CS and VD by age and year of birth in THIN-CPRD dataset

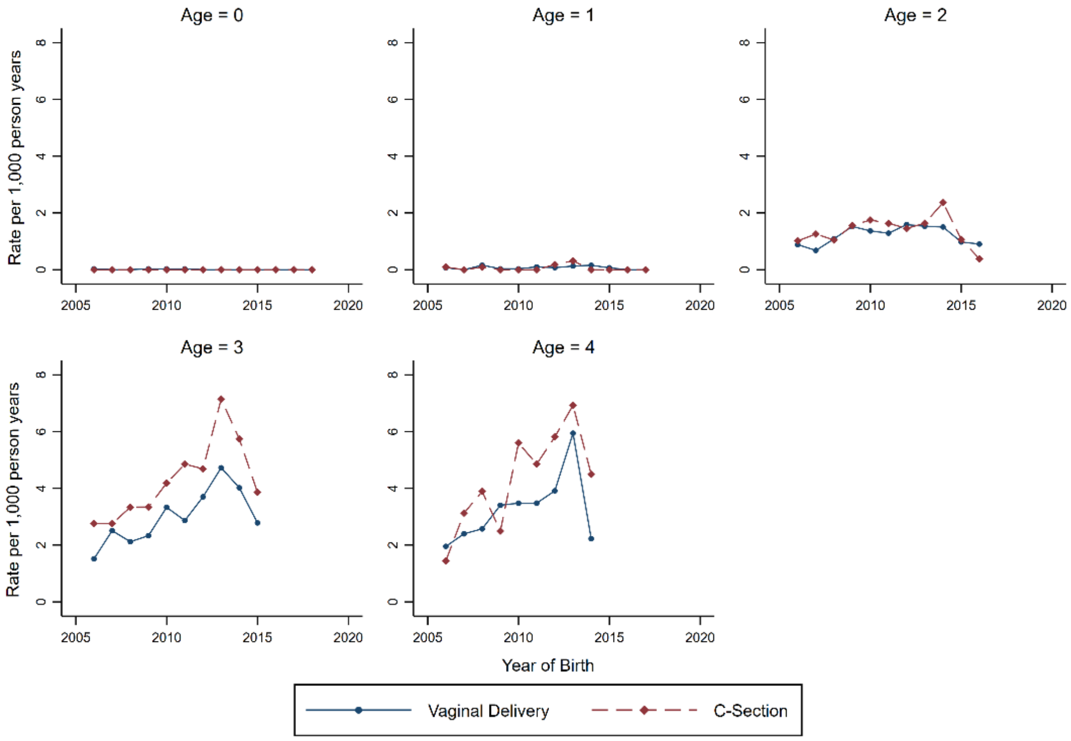


Figure S44 Incidence rate of autism spectrum disorder per 1,000 person years in children born by CS and VD by age and year of birth in THIN-CPRD dataset

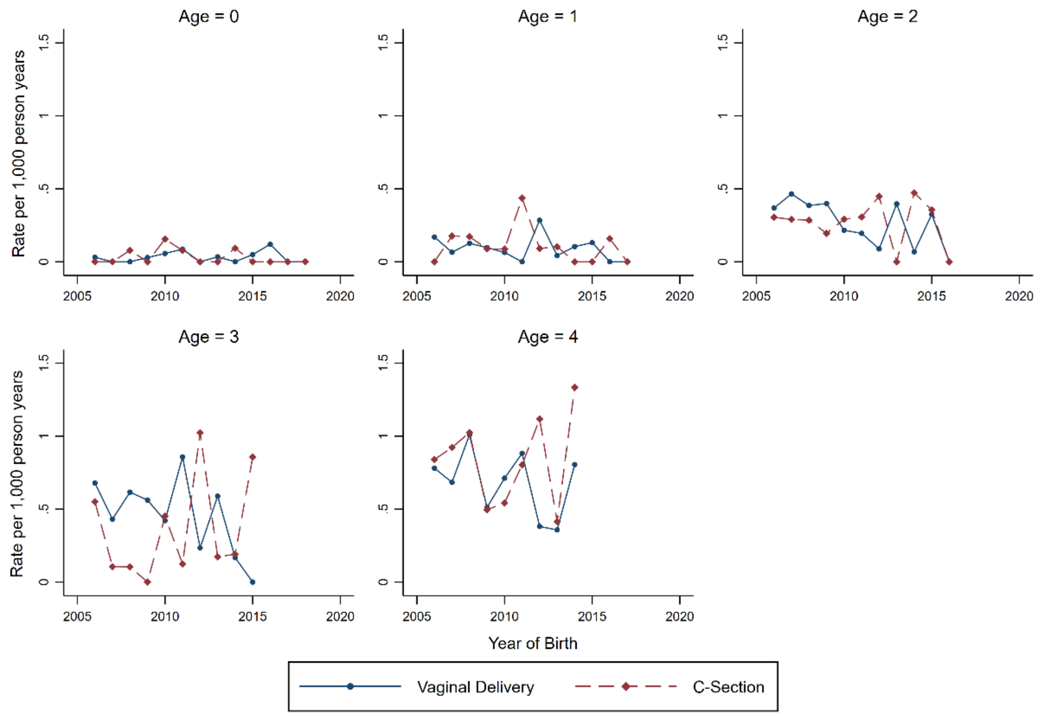


Figure S45 Incidence rate of ADHD per 1,000 person years in children born by CS and VD by age and year of birth in THIN-CPRD dataset

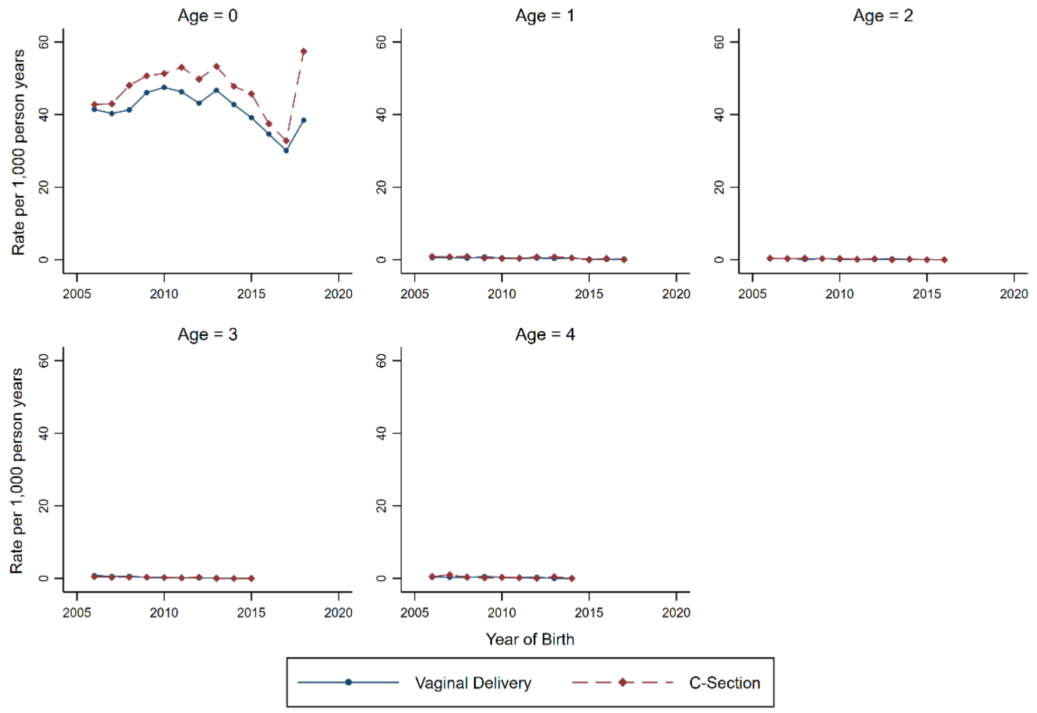


Figure S46 Incidence rate of colic per 1,000 person years in children born by CS and VD by age and year of birth in THIN-CPRD dataset

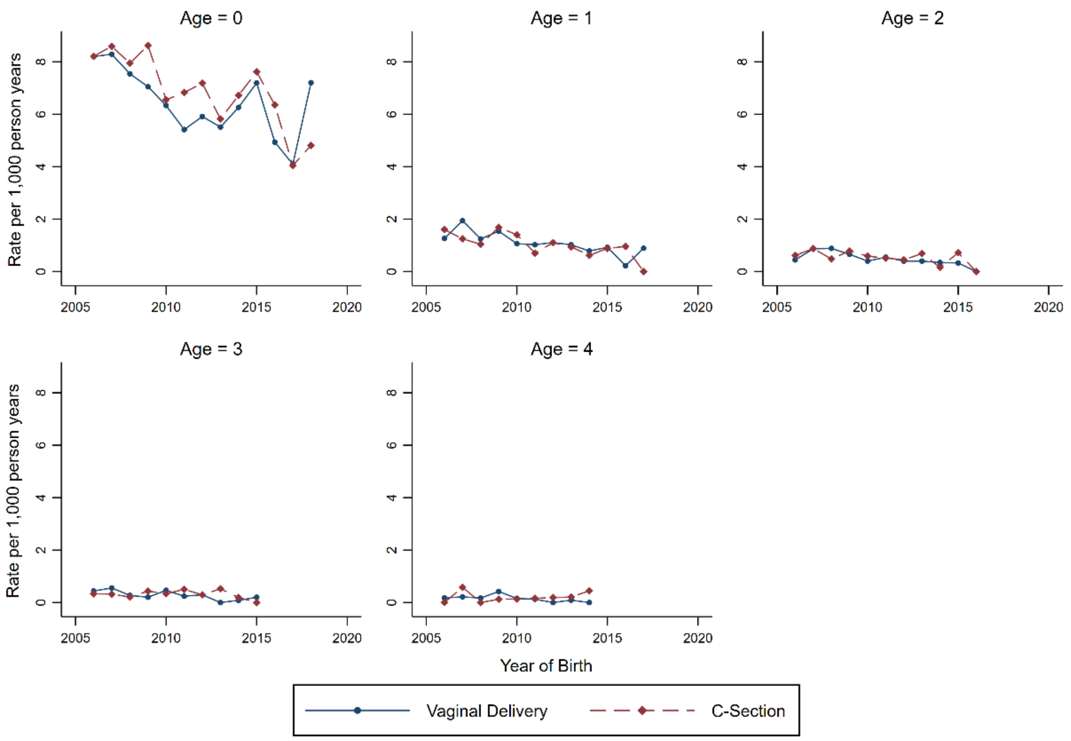


Figure S47 Incidence rate of failure to thrive per 1,000 person years in children born by CS and VD by age and year of birth in THIN-CPRD dataset

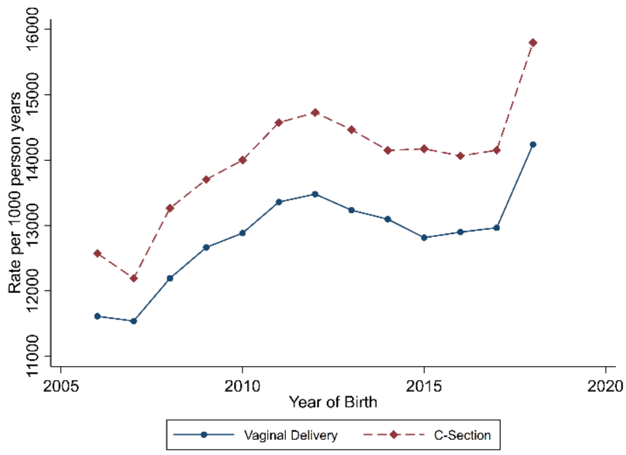


Figure S48 Incidence rate of primary care consultations in the first 12 months of life per 1,000 person years in children born by CS and VD by age and year of birth in THIN-CPRD dataset

## Maternal outcomes in THIN-CPRD dataset

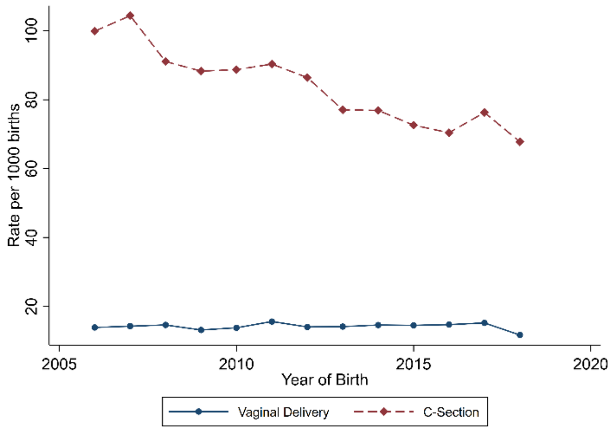


Figure S49 Incidence of maternal composite infectious morbidity (wound infection, endometritis/ endomyometritis, pelvic abscess, maternal sepsis) per 1,000 births by CS and VD by year of delivery in THIN-CPRD dataset

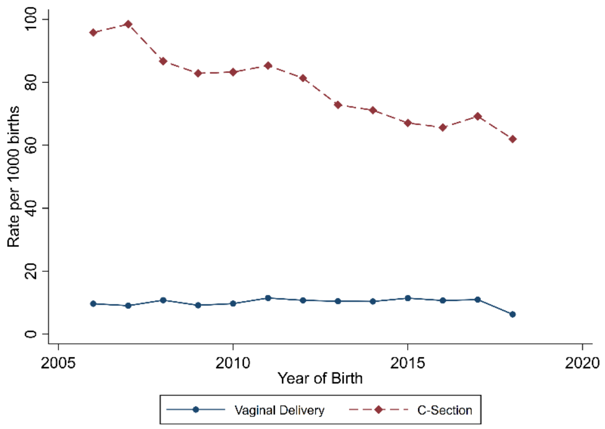


Figure S50 Incidence of wound infection per 1,000 births by CS and VD by year of delivery in THIN-CPRD dataset

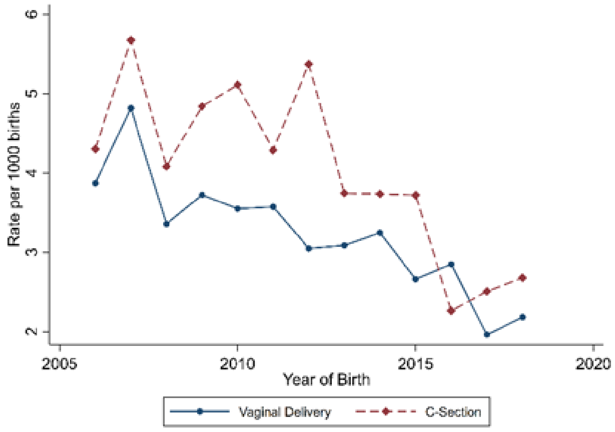


Figure S51 Incidence of endometritis/ endomyometritis per 1,000 births by CS and VD by year of delivery in THIN-CPRD dataset

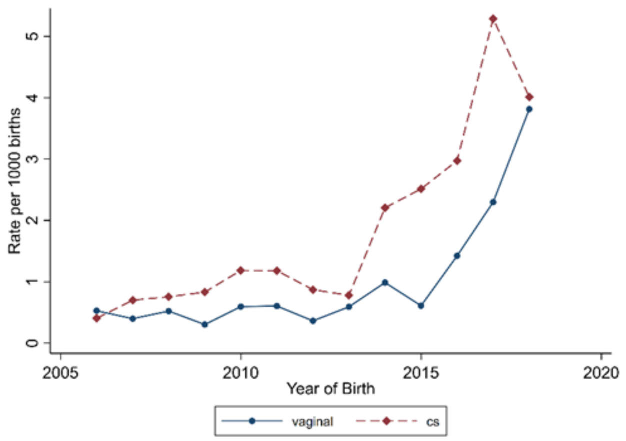


Figure S52 Incidence rate of maternal sepsis per 1,000 births by CS and VD by year of delivery in THIN-CPRD dataset

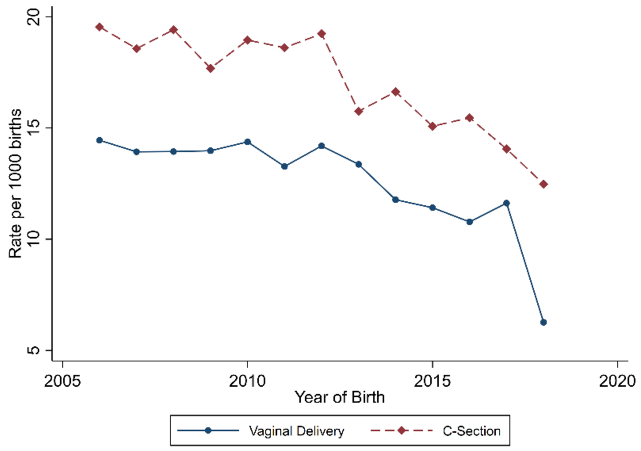


Figure S53 Incidence rate of maternal urinary tract infection per 1,000 births by CS and VD by year of delivery in THIN-CPRD dataset

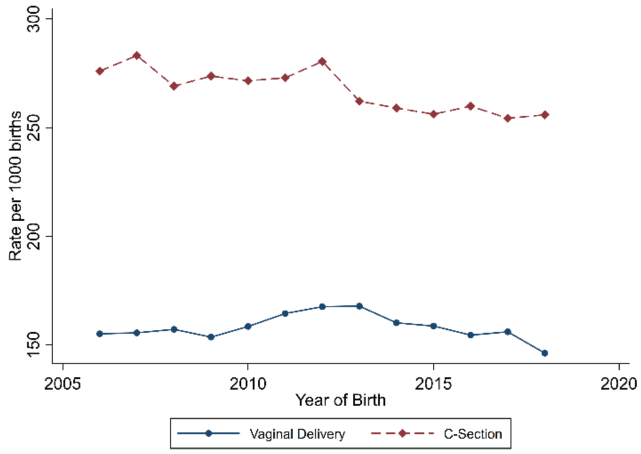


Figure S54 Incidence of antibiotic prescribing in post-partum period per 1,000 births by CS and VD by year of delivery in THIN-CPRD dataset

## Child outcomes in HES dataset

As HES captures all hospital admissions in England, all children were assumed to be followed up until the age of five years if they were born before 2015. For the birth years of 2015, 2016 and 2017, children were followed up only up to the age of four, three and two years respectively, therefore for these years the incidence rate reflects the incidence of the outcome of interest in these age groups.

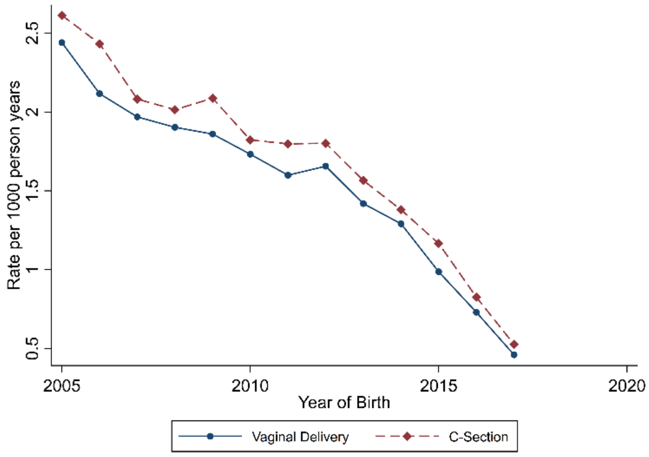


Figure S55 Incidence rate of asthma per 1,000 person years in children born by CS and VD by year of birth in HES dataset

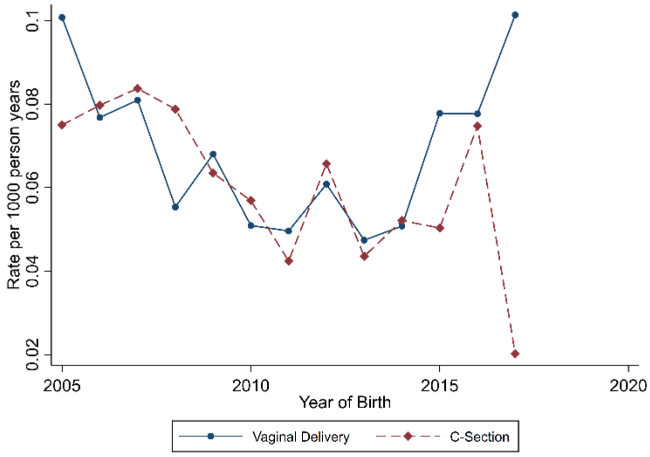


Figure S56 Incidence rate of eczema per 1,000 person years in children born by CS and VD by year of birth in HES dataset

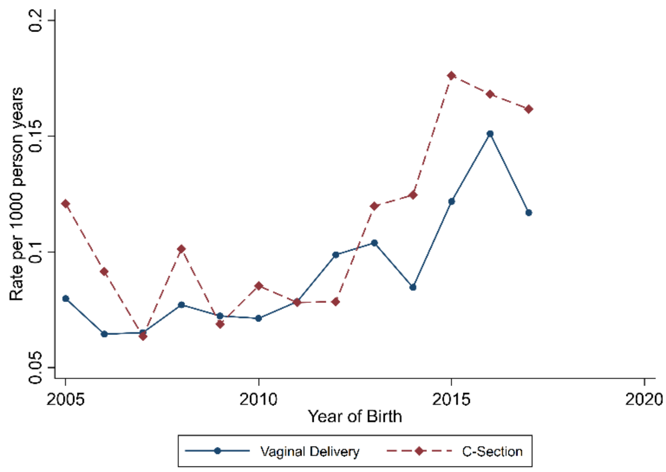


Figure S57 Incidence rate of anaphylaxis per 1,000 person years in children born by CS and VD by year of birth in HES dataset

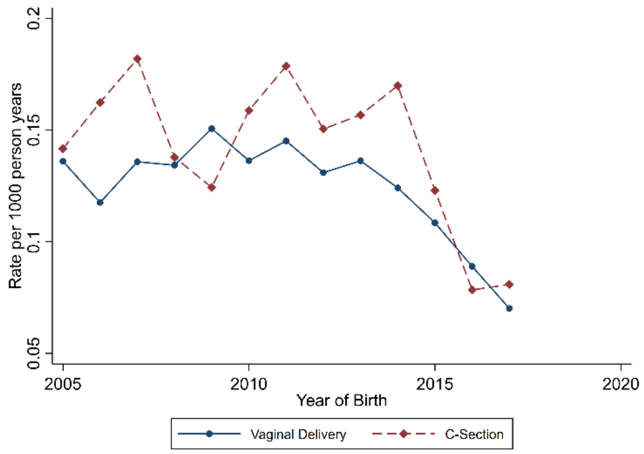


Figure S58 Incidence rate of type 1 diabetes per 1,000 person years in children born by CS and VD by year of birth in HES dataset

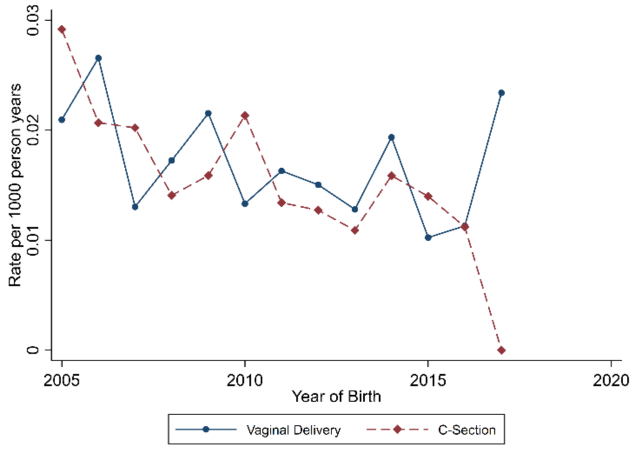


Figure S59 Incidence rate of juvenile idiopathic arthritis per 1,000 person years in children born by CS and VD by year of birth in HES dataset

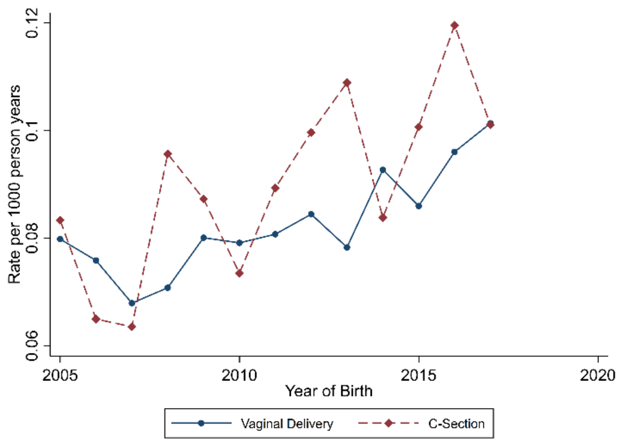


Figure S60 Incidence rate of autoimmune (idiopathic) thrombocytopenic purpura per 1,000 person years in children born by CS and VD by year of birth in HES dataset

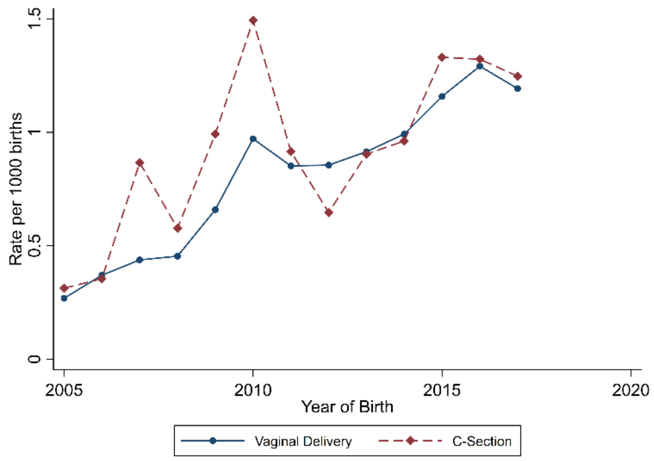


Figure S61 Incidence of early onset neonatal sepsis per 1,000 births in children born by CS and VD by year of birth in HES dataset

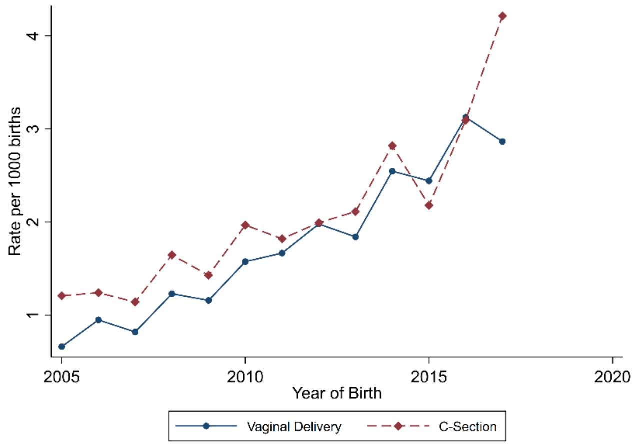


Figure S62 Incidence of late onset neonatal sepsis per 1,000 births in children born by CS and VD by year of birth in HES dataset

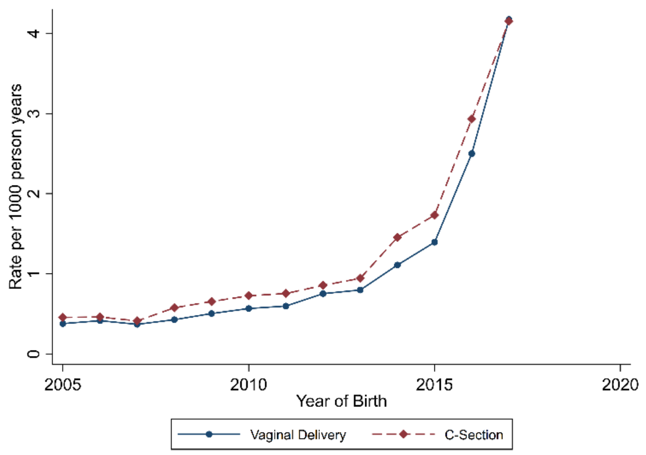


Figure S63 Incidence rate of other sepsis in children (developed after the neonatal period of 28 days after birth) per 1,000 person years in children born by CS and VD by year of birth in HES dataset

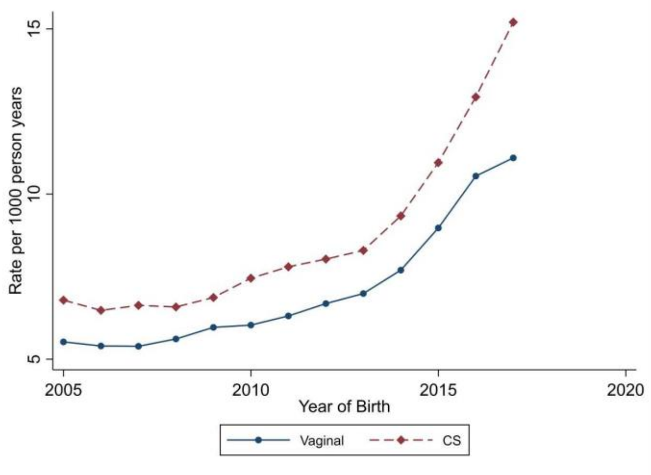


Figure S64 Incidence rate of lower respiratory tract infections per 1,000 person years in children born by CS and VD by year of birth in HES dataset

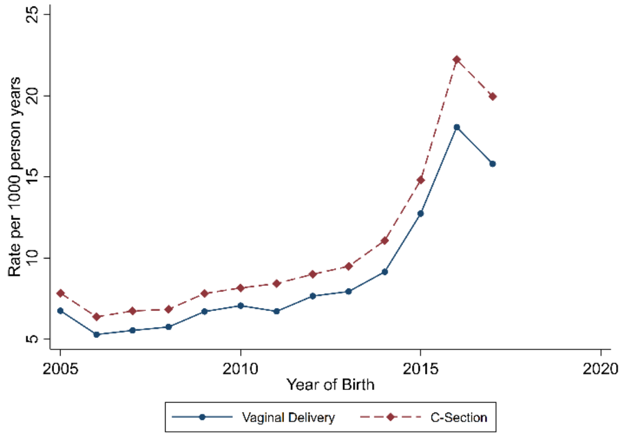


Figure S65 Incidence rate of bronchiolitis per 1,000 person years in children born by CS and VD by year of birth in HES dataset

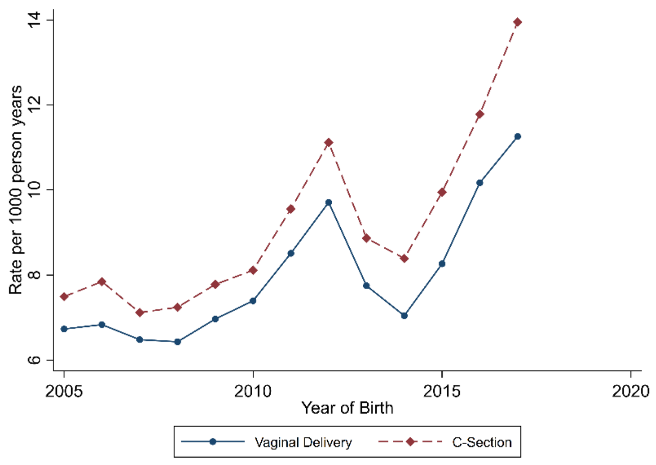


Figure S66 Incidence rate of gastroenteritis per 1,000 person years in children born by CS and VD by year of birth in HES dataset

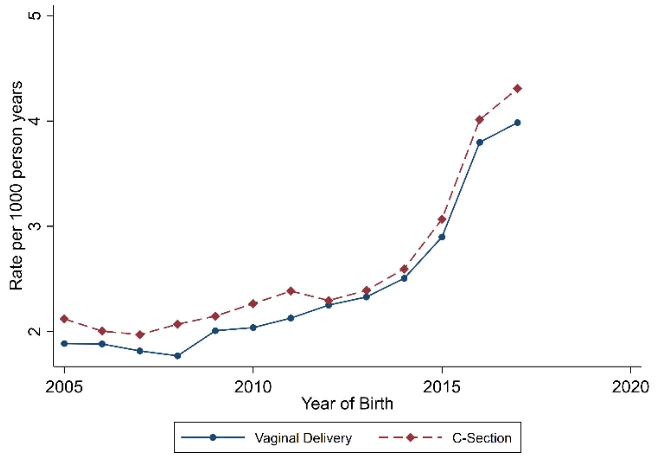


Figure S67 Incidence rate of urinary tract infections per 1,000 person years in children born by CS and VD by year of birth in HES dataset

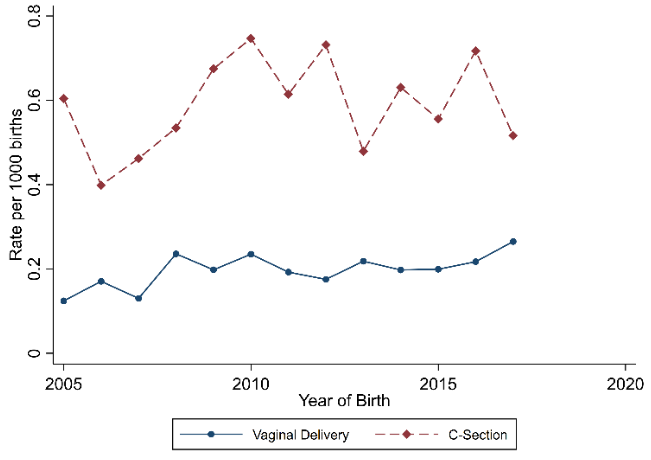


Figure S68 Incidence of necrotising enterocolitis per 1,000 births in children born by CS and VD by year of birth in HES dataset

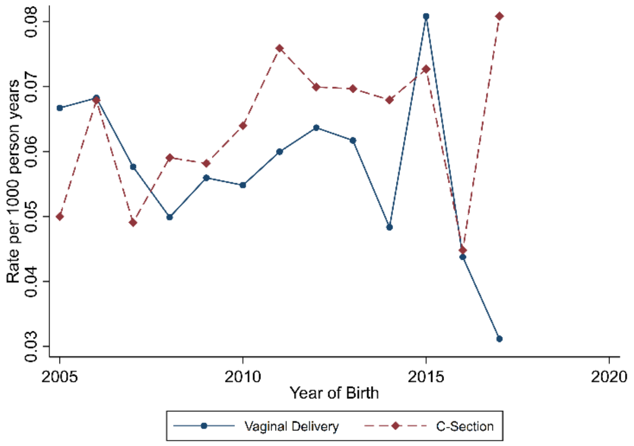


Figure S69 Incidence rate of leukaemia per 1,000 person years in children born by CS and VD by year of birth in HES dataset

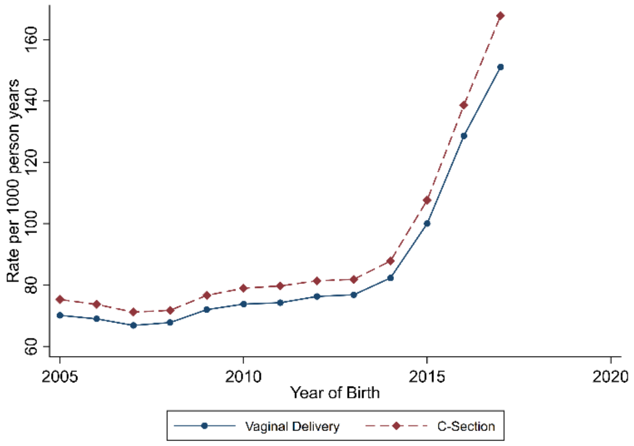


Figure S70 Incidence rate of first-time hospital admission per 1,000 person years in children born by CS and VD by year of birth in HES dataset

## Maternal outcomes in HES dataset

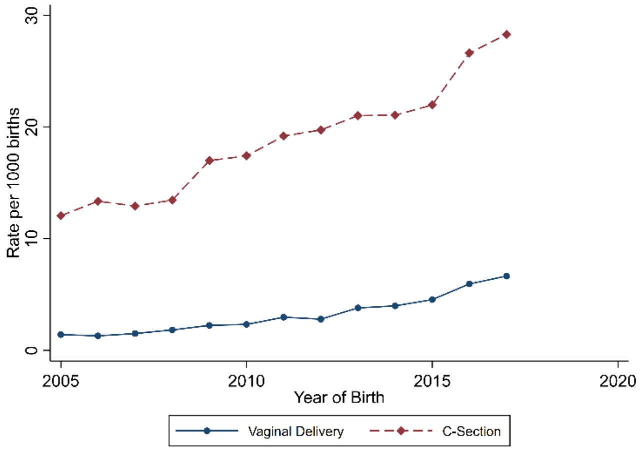


Figure S71 Incidence of maternal composite infectious morbidity (wound infection, endometritis/endomyometritis, maternal sepsis) per 1,000 births by CS and VD by year of delivery in HES dataset

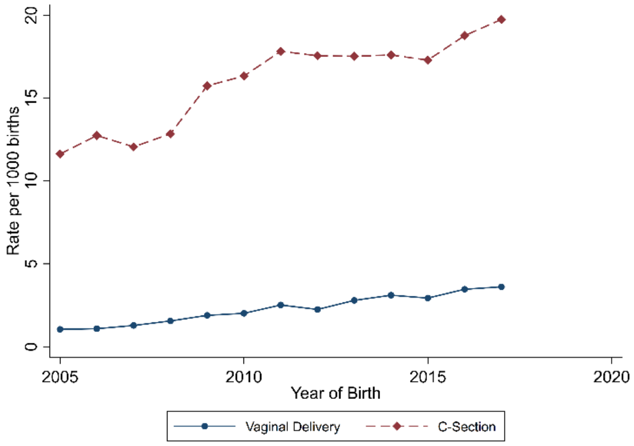


Figure S72 Incidence of wound infection, per 1,000 births by CS and VD by year of delivery in HES dataset

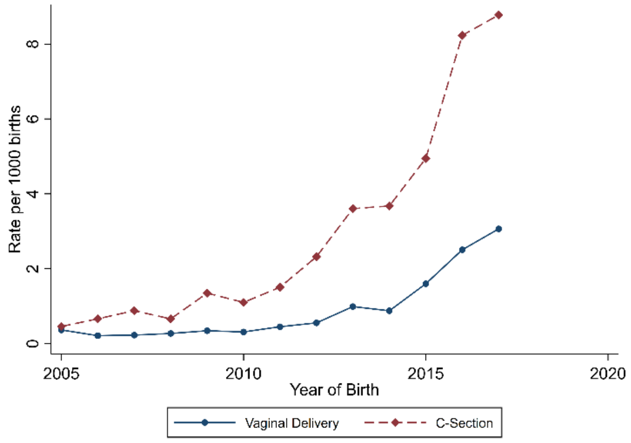


Figure S73 Incidence of maternal sepsis, per 1,000 births by CS and VD by year of delivery in HES dataset

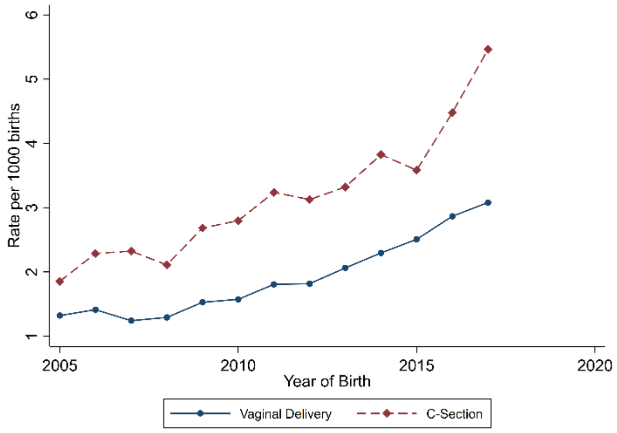


Figure S74 Incidence of maternal urinary tract infection per 1,000 births by CS and VD by year of delivery in HES dataset
